# Mycotoxin profiling of 1000 beer samples with a special focus on craft beer

**DOI:** 10.1371/journal.pone.0185887

**Published:** 2017-10-05

**Authors:** Jeroen Peters, Ruud van Dam, Ronald van Doorn, David Katerere, Franz Berthiller, Willem Haasnoot, Michel W. F. Nielen

**Affiliations:** 1 RIKILT Wageningen University & Research (Institute of Food Safety), Wageningen, the Netherlands; 2 Innosieve Diagnostics BV, Wageningen, the Netherlands; 3 Tshwane University of Technology, Department of Pharmaceutical Sciences, Pretoria, Republic of South Africa; 4 Christian Doppler Laboratory for Mycotoxin Metabolism and Center for Analytical Chemistry, Department of Agrobiotechnology (IFA-Tulln), University of Natural Resources and Life Sciences, Vienna, Tulln, Austria; 5 Wageningen University, Laboratory of Organic Chemistry, Wageningen, the Netherlands; Tallinn University of Technology, ESTONIA

## Abstract

Currently beer is booming, mainly due to the steady rise of craft breweries worldwide. Previous surveys for occurrence of mycotoxins in beer, were mainly focussed on industrial produced beer. The present survey reports the presence of mycotoxins in craft beer and how this compares to industrial produced beer. More than 1000 beers were collected from 47 countries, of which 60% were craft beers. A selection of 1000 samples were screened for the presence of aflatoxin B_1_, ochratoxin A (OTA), zearalenone (ZEN), fumonisins (FBs), T-2 and HT-2 toxins (T-2 and HT-2) and deoxynivalenol (DON) using a mycotoxin 6-plex immunoassay. For confirmatory analysis, a liquid chromatography tandem mass spectrometry (LC-MS/MS) method was developed and applied. The 6-plex screening showed discrepancies with the LC-MS/MS analysis, possibly due to matrix interference and/or the presence of unknown mycotoxin metabolites. The major mycotoxins detected were DON and its plant metabolite deoxynivalenol-3-β-D-glucopyranoside (D3G). The 6-plex immunoassay reported the sum of DON and D3G (DON+D3G) contaminations ranging from 10 to 475 μg/L in 406 beers, of which 73% were craft beers. The popular craft beer style imperial stout, had the highest percentage of samples suspected positive (83%) with 29% of all imperial stout beers having DON+D3G contaminations above 100 μg/L. LC-MS/MS analysis showed that industrial pale lagers from Italy and Spain, predominantly contained FBs (3–69 μg/L). Besides FBs, African traditional beers also contained aflatoxins (0.1–1.2 μg/L). The presence of OTA, T-2, HT-2, ZEN, β-zearalenol, 3/15-acetyl-DON, nivalenol and the conjugated mycotoxin zearalenone 14-sulfate were confirmed in some beers. This study shows that in 27 craft beers, DON+D3G concentrations occurred above (or at) the Tolerable Daily Intake (TDI). Exceeding the TDI, may have a health impact. A better control of brewing malts for craft beer, should be put in place to circumvent this potential problem.

## Introduction

Beer production and consumption is booming like never before, mainly due to the increasing popularity of craft beer. Craft beer is produced by small, independent and traditional breweries according to the definition of the Brewers Association. The number of craft breweries continues to grow, claiming a larger market share every year. In the US alone, a few hundred new craft breweries emerge annually. The total amount of craft breweries in 2014 saw an increase of 19.4% compared to 2013. Of the 3,464 breweries operating in the US, 3,418 were classified as craft breweries [1]. The same phenomenon is seen in other parts of the world. In the Netherlands, 108 new breweries emerged just in 2015, bringing the total brewery count to 382 [2]. The reason for the popularity of craft brewers is that they tend to focus on flavour and tradition, combined with innovation rather than on large-scale and low-cost production. This development already started with the Campaign for Real Ale (CAMRA) in Britain, 43 years ago [3]. Some craft breweries also produce similar beer styles as industrial brewers (e.g. pilsner beers). The vast majority of the craft breweries however, produce ancient beer styles, adjusted classic styles or even newly invented styles. Whereas “regular” beers are brewed almost exclusively with water, malted barley, hop and yeast, craft brewers add a wide range of different ingredients to the brewing process. Some examples are coffee, cacao, tobacco, liquorice, nuts, tomatoes, chili peppers, fruit and a range of spices [4, 5]. A quick count on one of the most popular websites for craft beer [6], shows that there are currently 83 unique beer styles. Because new styles are regularly being invented, this number will likely increase in coming years.

Mycotoxins are fungal metabolites with acute and/or chronic health effects on animals and humans. These effects include diarrhoea, reduced fertility, immunosuppression, cancer and even death [7–9]. Mycotoxins contaminate a wide range of cereals, including wheat [10], maize [11] and oats [12]. Barley is one of the key ingredients in beer and is prone to mycotoxin contamination [13–15]. Occurrence of mycotoxins in beer has been extensively surveyed, utilizing both instrumental analysis as well as immunoassays. A selected overview [16–45] is presented in Table 1. The most reported mycotoxins in beers, at relevant levels, are the type B trichothecene deoxynivalenol (DON), its plant metabolite deoxynivalenol-3-β-D-glucopyranoside (D3G) and fumonisins (FBs). DON and D3G were mainly reported in European beers, while FBs were mainly reported in beers from Africa and Southern Europe. In general, very high contaminations for all mycotoxins, besides T-2 toxin (T-2) and HT-2 toxin (HT-2), were reported previously in African beers. Ochratoxin A (OTA) was mainly reported in European beers, while aflatoxins (AFs) were mainly reported in African and Asian beers. T-2 toxin (T-2), HT-2 toxin (HT-2) and zearalenone (ZEN) were rarely reported. Most beer surveys in Table 1 lack detailed information about specific beer styles, country of origin or alcohol content. The presence of mycotoxins in hops, a key ingredient in beer, has rarely been investigated [46]. Furthermore, mycotoxins may also be introduced in beer upon the addition of commodities other than cereals. The risk of mycotoxin contamination may therefore be higher in craft brewing, where a wide range of commodities are added at various stages of the brewing process [47]. The question therefore arises, whether these new and revived craft beer styles contain more, or different, mycotoxins compared to regular commercial beers. Additionally, the changing climate may contribute to altered levels of mycotoxins in field crops [48, 49] which eventually will lead to altered levels of mycotoxins in beer.

**Table 1:** Overview of mycotoxin surveys in beer.

Mycotoxin	No. beers analysed	No. samples positive	Mycotoxin concentration range (μg/L)	Beer style	Highest contamination(Country)	Alcohol content (% ABV)	Author	Year	Ref
AFB_1_	116	13	0.0005–0.083	-	India	-	Nakajima et al.	1999	[16]
	304	12	0.0012–0.23	-	India	-	Mably et al.	2005	[17]
	422	271	0.00007–0.038	-	Ghana	-	Burdaspal and Legarda	2013	[18]
AFB_2_	116	5	0.0012–0.0086	-	India	-	Nakajima et al.	1999	[16]
	304	4	0.0156–0.032	-	India	-	Mably et al.	2005	[17]
AFs	422	273	0.00007–0.04518	-	France	-	Burdaspal and Legarda	2013	[18]
	5	5	0.0088–0.0345	African traditional	Malawi	-	Matumba et al.	2011	[19]
	35	3	12–400	African traditional	South Africa	-	Odhav and Naicker	2002	[20]
T-2	30	17	0.2*	-	-	-	Yoshinari et al.	2014	[21]
HT-2	30	2	0.6*	-	-	-	Yoshinari et al.	2014	[21]
	154	14	25.1–38.2	Wheat	Germany	-	Rodríguez-Carrasco et al.	2015	[22]
ZEN	23	1	<LOQ	Adjunct lager	USA	4.7	Zöllner et al.	2000	[23]
	91	10	0.46–0.55	-	Ireland	-	Kuzdralinski et al.	2013	[24]
	44	44	0.35–2.0	-	-	-	Bauer et al.	2016	[25]
	46	28	12.5–200	African traditional	Nigeria	-	Okoye	1985	[26]
	44	21	20–201	African traditional	Botswana	-	Nkwe et al.	2005	[27]
	35	7	2.6–426	African traditional	South Africa	-	Odhav and Naicker	2002	[20]
β-ZEL	23	1	0.264	Adjunct lager	USA	4.7	Zöllner et al.	2000	[23]
OTA	116	107	0.0017–0.066	-	Belgium	-	Nakajima et al.	1999	[16]
	61	30	0.010–0.135	-	Belgium	>6%	Visconti et al.	2000	[28]
	106	72	0.005–0.189	-	Denmark	-	Bertuzzi et al.	2011	[29]
	150	42	0.1–8.1	-	-	-	Gumus et al.	2004	[30]
	19	10	1.5–2340	African traditional	South Africa	-	Odhav and Naicker	2002	[20]
	88	73	0.007–0.204	-	Germany	-	Medina et al.	2005	[31]
Mycotoxin	No. beers analysed	No. samples positive	Mycotoxin concentration range (μg/L)	Beer style	Highest contamination(Country)	Alcohol content (% ABV)	Author	Year	Ref
OTA	20	0	<LOQ	-	-	-	Wu et al.	2011	[32]
	35	17	0.04–0.350	-	Tunisia	-	Lasram et al.	2013	[33]
FB_1_	106	32	0.1–30.3	-	Italy	-	Bertuzzi et al.	2011	[29]
	120	105	0.5–340	African traditional	Cameroon	-	Roger	2011	[34]
	18	18	38–1066	African traditional	South Africa	-	Shephard et al.	2005	[35]
	9	9	1522*	African traditional	Malawi	-	Matumba et al.	2014	[19]
	53	8	29–285	-	Brazil	-	Piacentini et al.	2015	[36]
FB_2_	106	19	0.2–3.9	-	Italy	-	Bertuzzi et al.	2011	[29]
	18	17	8–135	African traditional	South Africa	-	Shephard et al.	2005	[35]
	9	8	251*	African traditional	Malawi	-	Matumba et al.	2014	[19]
FB_3_	18	12	8–128	African traditional	South Africa	-	Shephard et al.	2005	[35]
	9	6	229*	African traditional	Malawi	-	Matumba et al.	2014	[19]
FB_1_ + FB_2_	72	64	157.2*	-	Spain	-	Cano-Sancho et al.	2012	[37]
	29	12	0.3–12.7	-	USA	-	Hlywka and Bullerman	1999	[38]
Total FBs	32	14	4.8–85.5	-	Spain	-	Torres et al.	1998	[39]
DON	313	272	4.0–56.7	-	Belgium	-	Papadopoulou et al.	2004	[40]
	20	18	5.1–35.9	Strong Pale Lager	-	9.0	Zachariasova et al.	2008	[41]
	15	15	5.6–62.2	-	-	-	Zachariasova et al.	2012	[42]
	176	113	1.0–35.9	Light beer	-	-	Kostelanska et al.	2009	[43]
			1.0–16.0	Dark beer	-	-	Kostelanska et al.	2009	[43]
	106	70	0.7–18.6	-	Croatia	-	Bertuzzi et al.	2011	[29]
	120	107	140–730	African traditional	Cameroon	-	Roger	2011	[34]
	91	91	6.0–70.2	-	Poland	-	Kuzdralinski et al.	2013	[24]
	217	118	5.4–89.3	Pale beer	Austria	4.9	Varga et al.	2013	[44]
	46	36	5.2–49.6	Wheat	Germany	4.9	Varga et al.	2013	[44]
	47	14	11.1–45.0	Dark beer	Germany	5.3	Varga et al.	2013	[44]
	20	18	6.5–27.1	Bock beer	Germany	11.0	Varga et al.	2013	[44]
	19	5	3.2–26.1	Non-alcoholic	Serbia	0.5	Varga et al.	2013	[44]
	25	13	4.2–12.7	Shandy	Serbia	2.0	Varga et al.	2013	[44]
	61	14	200–360	Busaa	Kenya	-	Kirui et al.	2014	[45]
	154	92	24.5–47.7	-	Spain	-	Rodríguez-Carrasco et al.	2015	[22]
	53	17	127–501	-	Brazil	-	Piacentini et al.	2015	[36]
	44	33	2.2–20	-	-	-	Bauer et al.	2016	[25]
ADONs	20	15	5.1–27.6	Strong Pale Lager	-	9.0	Zachariasova et al.	2008	[41]
Mycotoxin	No. beers analysed	No. samples positive	Mycotoxin concentration range (μg/L)	Beer style	Highest contamination(Country)	Alcohol content (% ABV)	Author	Year	Ref
ADONs	176	88	1.0–25.0	Light beer	-	-	Kostelanska et al.	2009	[43]
	176	88	1.0–24.0	Dark beer	-	-	Kostelanska et al.	2009	[43]
D3G	20	19	4.0–25.8	Pale Lager	-	5.0	Zachariasova et al.	2008	[41]
	15	15	6.0–82.1	-	-	-	Zachariasova et al.	2012	[42]
	176	130	1.4–37.0	Light beer	-	-	Kostelanska et al.	2009	[43]
			1.5–26.0	Dark beer	-	-	Kostelanska et al.	2009	[43]
	217	142	3.6–81.3	Pale beer	Austria	4.9	Varga et al.	2013	[44]
	46	32	3.5–28.4	Wheat	Germany	4.9	Varga et al.	2013	[44]
	47	28	4.2–26.2	Dark beer	Germany	5.3	Varga et al.	2013	[44]
	20	20	2.4–33.3	Bock beer	Germany	11.0	Varga et al.	2013	[44]
	19	9	1.6–6.6	Non-alcoholic	Serbia	0.5	Varga et al.	2013	[44]
	25	20	1.8–7.9	Shandy	Austria	2.2	Varga et al.	2013	[44]

**Mycotoxin abbreviations**: AFB_1_ (aflatoxin B_1_), AFB_2_ (aflatoxin B_2_), AFM_1_ (aflatoxin M_1_), AFs (aflatoxins), T-2 (T-2 toxin), HT-2 (HT-2 toxin), ZEN (zearalenone), β-ZEL (β-zearalenol), OTA (ochratoxin A), FB_1_ (fumonisin B_1_), FB_2_ (fumonisin B_2_), FB_3_ (fumonisin B_3_), FBs (fumonisins), DON (deoxynivalenol), D3G (deoxynivalenol-3-β-D-glucopyranoside) and ADONs (sum of 3-acetyl-DON and 15-acetyl-DON).

%ABV = percentage alcohol by volume,—Information not available;

* Maximum contamination reported in article (range not reported in article),

LOQ = limit of quantification

In this work we present a large-scale survey for mycotoxin occurrence in 1000 beer samples with a unique outlook on the upcoming and strongly expanding craft beer market. Beer samples of many different beer styles (representing 60% craft beers) were collected throughout the world, but with a detailed focus on European countries. This selection of 1000 samples was investigated for mycotoxin contamination and to elucidate possible differences between industrial beers and craft beers. Furthermore this survey aimed for a detailed look into the possible occurrence of conjugated (masked) mycotoxins in beer. To facilitate the fast mycotoxin multiplex screening of 1000 beer samples, a previously developed 6-plex microsphere immunoassay method [50, 51] for the detection of DON and D3G, aflatoxin B_1_ (AFB_1_), OTA, the sum of T-2 and HT-2, fumonisins (sum of fumonisin B_1_ (FB_1_), B_2_ (FB_2_) and B_3_ (FB_3_) and ZEN in barley, was modified and adapted for beer samples. To confirm the presence of mycotoxin contaminations in a subset of the screened beer samples, a dedicated multi-mycotoxin liquid chromatography tandem mass spectrometry (LC-MS/MS) method for beer was developed. This method includes several conjugated mycotoxins, as well as mycotoxin metabolites, such as aflatoxin M_1_ (AFM_1_), ochratoxin B (OTB), nivalenol (NIV) and zearalenone 14-sulfate (Z14S).

This unprecedented survey reveals the discovery of the conjugated mycotoxin Z14S in beer and confirms that high DON and D3G contaminations can specifically occur in craft beer. The sum of these DON and D3G contaminations (DON+D3G) contribute to surpassing the tolerable daily intake (TDI) of DON upon moderate beer consumption.

## Materials and methods

### Instrumentation

The 6-plex microsphere immunoassay was performed on a flow cytometer platform (FM3D) or on a planar bead array analyzer (MAGPIX), both running on XPONENT software (all from Luminex, Austin, USA). Mycotoxin concentrations were calculated using the xMAP dedicated Bio-Plex manager software 6.0, combined with Bio-Plex results generator 3.0 (Bio-Rad Laboratories, Veenendaal, the Netherlands). A Bio-Plex II Wash Station (Bio-Rad) with magnetic plate support was used for all washing steps. Incubation of the 6-plex assays was done on a Bühler TiMix 2 shaker (Salm en Kipp, Breukelen, the Netherlands) at room temperature (RT). Beer samples were degassed at RT using the Ultrasonic Cleaner (VWR International, Amsterdam, the Netherlands) at maximum power and centrifuged in an Eppendorf 5810R centrifuge (VWR) equipped with an A-4-62 swinging bucket rotor. All confirmatory analyses of mycotoxins in selected beer samples were done on an AB Sciex (Nieuwerkerk a/d IJssel, the Netherlands) QTRAP 5500 tandem mass spectrometer (MS/MS) equipped with an electrospray ionization (ESI) source, operated in positive and negative multiple reaction monitoring (MRM) mode. The MS system was coupled to a Shimadzu (‘s Hertogenbosch, the Netherlands) Prominence Liquid Chromatography (LC) system, equipped with a Restek (Interscience, Breda, the Netherlands) Ultra Aqueous C18 (100×2.1 mm) column (see Supporting Information (S.I.)). Integration of reconstructed MRM chromatograms was done with MultiQuant V2.0 software using the Signal Finder integration algorithm (AB Sciex). Monoclonal antibodies (mAbs) against AFB_1_, ZEN, T-2 and DON were purchased from Aokin AG (Berlin, Germany), while the FB_1_ and OTA mAbs were purchased from Soft Flow Biotechnology Ltd. (Gödöllő, Hungary). The R-Phycoerythrin (RPE)-FB_1_ and RPE-OTA conjugates were produced in-house using RPE from Moss (Pasadena, MD, USA). The remaining RPE-mycotoxin conjugates were synthesized by Aokin.

### Chemicals

Cellstar 96-well culture microtiter plates, 10 and 50 mL tubes were from Greiner (Alphen a/d Rijn, the Netherlands). Sheath fluid (FM3D) and drive fluid (MAGPIX) were both purchased from Luminex (Austin, USA). The following mycotoxins and metabolites were purchased from Biopure (Tulln, Austria): FB_1_, FB_2_, FB_3_, OTA, OTB, AFB_1_, aflatoxin B_2_ (AFB_2_), aflatoxin G_1_ (AFG_1_), aflatoxin G_2_ (AFG_2_), aflatoxin M1 (AFM_1_), T-2, HT-2, DON, D3G, 3-acetyl-DON (3ADON), 15-acetyl-DON (15ADON), NIV, ZEN, α-zearalenol (α-ZEL) and β-zearalenol (β-ZEL). Zearalenone-14-β-D-glucopyranoside (Z14G), α-zearalenol-14-β-D-glucopyranoside (α-ZELG), β-zearalenol-14-β-D-glucopyranoside (β-ZELG) and zearalenone 14-sulfate (Z14S) were produced according to [52] or isolated from *Fusarium* inoculated rice. Syringeless filter devices (Mini-UniPrep, PTFE) for sample clean-up were purchased from GE Healthcare (Rotterdam, the Netherlands). Acetonitrile (ACN) and methanol (MeOH) were purchased from Biosolve (Valkenswaard, the Netherlands), formic acid (FA) from Merck (Amsterdam, the Netherlands) and ammonium formate (AMF) from Fluka Analytical (Steinheim, Germany). All other chemicals were purchased from VWR International (Amsterdam, the Netherlands).

### Beer samples

A total of 1000 beer samples, from 42 different countries (Table 2), were selected. Sample collection was mainly random and depending on access and availability, i.e., sampling was not intended to be statistically representative. Primary goal was to collect as much as possible craft beer samples to be able to make a comparison to industrial produced beer and have a more detailed look on craft beer itself. Secondary goal was to cover many different beer styles. At the start of this research, it was still difficult to collect a representative number of international craft beers due to poor availability. Craft beers were mainly collected from bars, restaurants, supermarkets, specialized craft-beer shops and during craft-beer festivals between 2011 and 2014, while industrial beers were mainly collected in supermarkets. Craft beer samples also included vintage beers (beers produced before 2011, often cellared for conditioning). Based on local contacts, additional samples from the USA, China and several African countries were sent to the authors. African beer samples were both commercial (bought in South African supermarkets) and traditional opaque home-brews (collected on site in town villages in Northern South Africa). From each beer, 10 mL was collected in a 50 mL tube and degassed by sonication at maximum power for 10 minutes at RT. After sonication, the beer samples were transferred to a 10 ml tube, centrifuged at 3200g and stored at -20°C. The designated beer styles of these samples were grouped into 20 main beer styles (Table 3).

**Table 2:** Origins of the 1000 beer samples investigated.

Country	Region	Number	Country	Region	Number	Country	Region	Number
Australia	Oceania	5	India	Asia	1	Poland	Europe	27
Austria	Europe	4	Ireland	Europe	3	Portugal	Europe	2
Belarus	Europe	1	Italy	Europe	28	Russia	Asia	1
Belgium	Europe	203	Jamaica	North-America	1	Scotland	Europe	12
Canada	North-America	6	Japan	Asia	9	Slovakia	Europe	1
China	Asia	5	Kenya	Africa	2	South Africa	Africa	46
Czech Republic	Europe	24	Latvia	Europe	1	Spain	Europe	48
Denmark	Europe	55	Malaysia	Asia	1	Sweden	Europe	5
England	Europe	38	Mexico	South America	4	Switzerland	Europe	3
Finland	Europe	1	Namibia	Africa	3	Trinidad & Tobago	North-America	1
France	Europe	16	Netherlands	Europe	209	Turkey	Europe	1
Germany	Europe	87	Nigeria	Africa	1	Ukraine	Europe	1
Greece	Europe	2	Norway	Europe	14	USA	North-America	124
Iceland	Europe	1	Peru	South America	1	Zimbabwe	Africa	2
**Total**	**1000**							
**Total Countries**	**42**							

**Table 3:** Grouping of individual beer samples into beer style groups and fraction of craft beers.

Group	%ABV*	Styles	Number analysed	Number craft beers	Percentage Craft beers
Non/low alcohol	< 3	Pale lager, Low alcohol, Non alcoholic	36	3	8
Pale Lager	3–5	Pilsener, helles lager, Adjunct lager, premium, Zwickel, California Common	166	11	7
Strong Pale Lager	6–14	Strong Pale lager, Imperial Pils	8	-	0
Pale Ale	4–9	Blond, Belgian Pale Ale, American Pale Ale, Amber Ale, Irish Ale, English Pale Ale, Mild Ale, Kölsch	94	63	67
Strong Pale Ale	9–15	Tripel (Abbey, Trappist), Barley Wine (American, English), Strong Ale (Belgian, American, English)	69	56	81
India Pale Ale	≤ 7.5	Bitter, Premium Bitter (ESB), India Pale Ale (Black, White)	42	37	88
Double India Pale Ale	≥ 7.5	India Pale Ale (Imperial, Triple, Double)	29	29	100
Dark lager	3–5	Schwarzbier, Dunkel	28	1	4
Dark Ale	6–9	Old Ale, Scotch, Dubbel (abbey, Trappist)	36	22	61
Strong Dark Ale	9–13	Quadrupel, Abt	44	28	64
Stout	< 8	Stout (Milk, Foreign, Oatmeal, Sweet), Porter	54	40	74
Imperial Stout	≥ 8	Stout (Imperial, Export), Porter (Imperial, Baltic)	126	123	98
Sour ales	4–13	Geuze, Lambic (Fruit, Faro, Unblended), Sour Ale, Gose, Wild Ale, Flanders Red, Flanders Oud Bruin, Berliner Weisse	82	72	88
Fruit/Vegetable/Spice	5–16	Various styles	37	25	68
Saison	4–11	Saison, Bière de Garde	13	10	77
Smoked	5–11	Various styles	16	12	75
Wheat	5–8	Weizen, Weizen (Dunkel, Bock), Wit, Belgian White, Wheat Ale	42	14	33
Bock	5–12	Bock (Helles, Doppel, Dunkel, Lente)	38	19	50
Eisbock	9–40	Eisbock	6	5	83
African traditional	< 3	Mqombothi, Sorghum	34	33	97
		**TOTAL**	1000	589	59

*Percentage alcohol by volume

### Screening of beer samples

**Fig 1:**
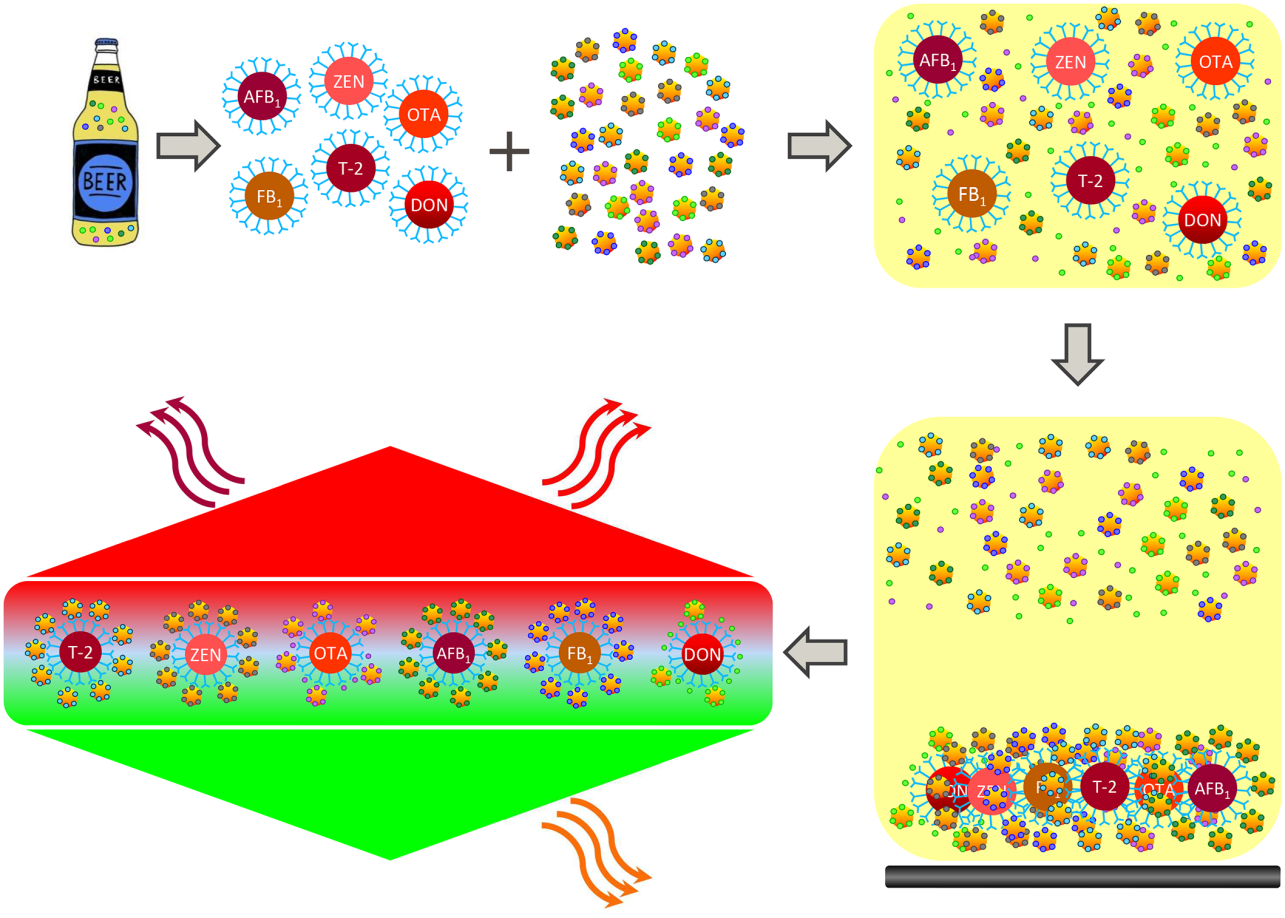
Overview of the 6-plex mycotoxin immunoassay: Mycotoxin mAb coupled paramagnetic beads and a mixture of mycotoxin specific reporter molecules (mycotoxins coupled to R-phycoerythrin) are added to a diluted beer sample. Competition occurs between the free mycotoxins present in the beer and the added mycotoxin-reporter molecules for antibody interaction on the beads. Next, the beads are captured at the bottom of the well by magnetic force. Remaining, non-interacting, assay components are removed by washing. Beads are then measured using a red light source for mycotoxin assay classification and a green light source for quantification of the reporter signal.

The mycotoxin 6-plex paramagnetic microsphere immunoassay method used, was a new adaptation of a previously described method [51]. The adapted mycotoxin 6-plex immunoassay involved a simplified extraction method and its performance in beer samples was validated on the planar array imaging platform. The stored beer samples were defrosted, mixed by inversion, and centrifuged for 10 minutes at 3200 g to pellet yeast or other insoluble matter. The supernatant was then diluted 8 fold using methanol-water (1:9 v/v) (10% MeOH). To 40 μL of the diluted beer samples a 10 μL mixture of microspheres, previously coupled with mycotoxin specific monoclonal antibodies, was added followed by the addition of 10 μL of a mixture of mycotoxin specific reporter molecules (mycotoxins coupled to RPE). The final buffer composition in the assay was phosphate buffered saline, 0.02% Tween 20, pH 7.4. Sample and assay components were incubated for 15 minutes, to allow competition between the mycotoxin-RPE conjugates and the free mycotoxins in the samples for antibody interaction. The microspheres were then trapped by a magnet and washed followed by analysis on one of the microsphere dedicated platforms (Fig 1). Since the beer styles investigated are very diverse in composition, it is rather impossible to find a suitable common blank beer. As a practical solution, we selected a dark ale as blank beer for all screening assays, following confirmation of the absence of mycotoxins by LC-MS/MS. This blank beer was used to prepare beer-based multi-mycotoxin calibration curves for AFB_1_, OTA, ZEN, DON, T-2 and FB_1_. First, the blank beer was diluted 4-fold with 10% MeOH and subsequently mixed (1:1, v/v) with each standard of the mycotoxin used for the construction of calibration curves, resulting in a final 8-fold dilution of the matrix content and a 2-fold dilution of each standard. Using these multi-mycotoxin calibration curves (S1 Fig), the mycotoxin concentrations in 1000 beer samples were calculated. To this end, the dedicated Bio Plex manager software (Bio-Rad Laboratories, Veenendaal, the Netherlands) built for automated curve and data fitting, was used. The results of the triplicate sample measurements are displayed as a concentration range combined with a standard deviation to give a better idea about the performance of the multiplex method. The mycotoxin monoclonal antibodies (mAbs) in the 6-plex immunoassay were previously tested for cross-reactivity [50, 51]. These results showed, amongst others, cross-reactivity of D3G in the DON immunoassay.

### Confirmation of screening results by LC-MS/MS

From the 1000 beer samples that were screened with the mycotoxin 6-plex immunoassay, 100 beer samples were selected for confirmatory LC-MS/MS analysis. The first set of samples submitted for confirmatory analysis, was based on mycotoxin contamination results revealed by the 6-plex screening assay. It included mostly high contaminations, as well as some blanks, revealed by the screening. Furthermore, the first selection was also based on covering a wide range of beer styles. Based on the confirmatory results of the first selection, a more detailed selection of new (previously screened) beers was made. The focus was on observed contamination trends in certain beer styles. Since craft beer was the main focus of this survey, we aimed for a total of 70% of craft beers in the final LC-MS/MS selection. For confirmatory analysis we adapted an existing ISO 17025 accredited LC-MS/MS method for feed. This adapted LC-MS/MS method (S1 Text) contained all the relevant mycotoxins, as well as a selection of available mycotoxin metabolites and conjugated forms, relevant to the 6-plex screening method targets (S1 Table). Since the variation in beer matrices is very diverse, especially in craft beer, we chose to use a single point standard addition method for quantification. To this end, 100 μl of degassed beer sample was diluted with 100 μl of the standard solution. The diluted sample was filtered through a syringeless filter device and 5 μl was injected. The limit of quantification (LOQ) was set at 10 times below the standard addition level. The upper quantification limit was set arbitrarily at 2 times above the standard addition level. The concentrations for standard additions can be found in the S1 Table. The limit of detection (LOD) was set at a signal-to-noise ratio of 3:1 based on the peak-to-peak noise around the retention times of the analytes in the reconstructed MRM chromatograms. To this end, we utilized MultiQuant V2.0 software (AB Sciex) using the Signal Finder integration algorithm.

## Results and discussion

### Scope of the survey

**Fig 2:**
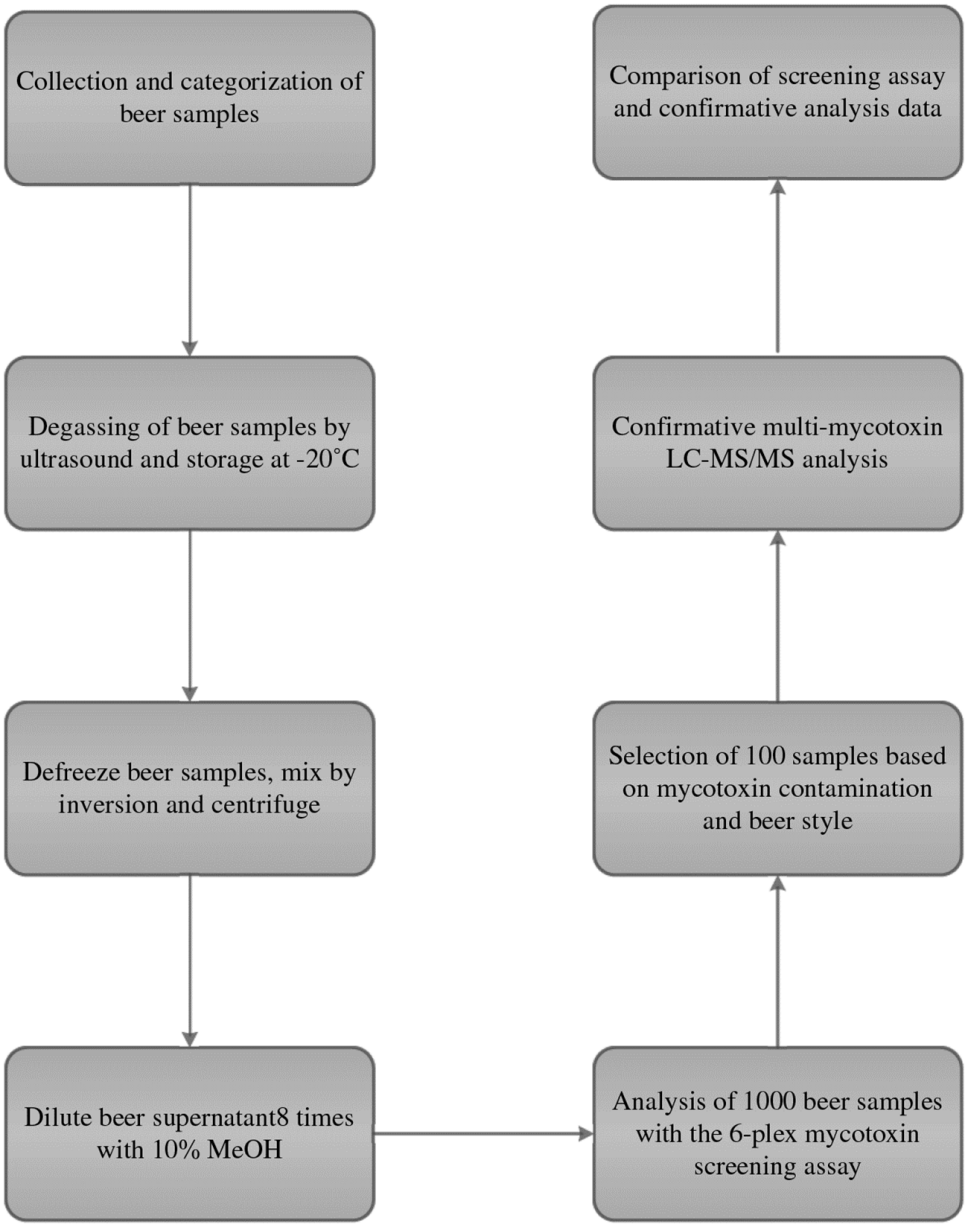
Schematic overview of the general approach.

Until now, most published beer surveys for mycotoxins are lacking relevant information on the beer styles (Table 1). Occasionally, information about the country of origin is supplied [29] and sometimes beers are grouped on the basis of their alcohol content [43]. More recently, Varga et al [44] provided detailed information about alcohol content, country of origin and beer style categories. Because of the serious style expansion by craft brewers, we chose to elaborate even further on the beer style categories while also focusing on alcohol content and origin. In our large-scale survey of 1000 beer samples from all over the world (S2 Fig), there was a strong focus on Europe with a total of 787 beers screened. Within Europe the emphasis was on beers from the Netherlands (209), Belgium (203) and Germany (87) (S2 Fig). Furthermore, nearly 60% of all the beers analysed were craft beers. A flow chart overview of the general survey approach is given in Fig 2.

### Performance of the 6-plex immunoassay as a screening method

A mycotoxin 6-plex immunoassay, previously applied as a qualitative screening assay for barley [51], was adapted for beer (Fig 1). The extraction protocol was simplified and the method was made suitable for the fast and semi-quantitative detection of mycotoxins in beer. The performance of the adapted 6-plex immunoassay was bench-marked against the previously developed, and in-house validated screening assay for barley [51], by determining the intra- and interday precision for beer samples. The intra- and interday relative standard deviation (%RSD) for multi-mycotoxin fortified samples in dark ale, based on the B/B_0_ values, were determined for each fortified sample and compared to the previous method (S2 Table). Also in beer, for most mycotoxins, the %RSD values were well below 10%. For T-2 the %RSD values were below 20%. This may indicate that the beer matrix interferes somewhat more with the T-2 assay than barley did. For quantification we prepared multi-mycotoxin dose-response curves in dark ale and checked the variation by comparing intra- and interday IC50s (S3 Table). Based on these dose-response curves, the mycotoxin concentrations in the multi-mycotoxin fortified samples were determined by the Bio Plex manager software. The intra- and interday quantitative precision is displayed in S4 Table. Besides the FB_1_ assay, which showed overestimations up to a factor 3.9, the other assays performed satisfactory with only slight overestimations with a factor ranging from 1.1 to 1.7. The overestimations in the FB_1_ assay were not unexpected and reported previously [50]. Moreover, the mycotoxin mAbs used in the 6-plex, in most cases, show cross-reactivity with other metabolites as was reported in detail previously [51]. Therefore, we chose to confirm selected results (see Method section for selection criteria) of the screening assay by an LC-MS/MS based method. The complete mycotoxin 6-plex screening data for all 1000 beers, grouped by beer style groups, can be found in the S5 Table.

### Performance of the LC-MS/MS confirmative method

A multi-toxin ISO 17025 accredited LC-MS/MS method for feed was successfully adapted for beer. In order to make the LC-MS/MS method fit for purpose, 23 irrelevant mycotoxins and metabolites were removed from the original 40 targets and 8 new mycotoxin metabolites and conjugated forms of interest were added. The standard addition quantification method was found fit for purpose, following fortification of 5 diverse beer styles (pale lager, adjunct lager, dark ale, sour ale and imperial stout) with all mycotoxins and metabolites. This resulted in adequate detection of precursor and product ions in the respective reconstructed MRM chromatograms. Average variation of LC retention times were well below 0.2 minutes and the deviations in MS/MS product ion intensity ratios, when comparing beer samples to non-matrix-matched standards (in 10% MeOH), were typically below 30% (occasionally below 50%). For DON, higher deviations occurred mainly at low concentrations (< 10 μg/L) and were not linked to any specific beer style. For both methods, the 6-plex immunoassay and the LC-MS/MS, we chose to simply dilute the beer samples rather than to perform concentration or clean-up steps. Therefore, potential matrix effects were only reduced by dilution in both the 6-plex immunoassay and LC-MS/MS. Since the beer styles analysed vary strongly in composition and gravity, variable matrix interference and signal suppression (or enhancement) were to be expected. As a consequence, some mycotoxin standard additions (mainly in imperial stouts) were hardly visible in the reconstructed MRM chromatograms for some of the beer samples. These samples were not considered nor reported. Please note that the sample dilution used for LC-MS/MS is smaller than for the 6-plex immunoassay, since the standard addition method allows correction for matrix effects. The actual volume of the beer sample analysed in the LC-MS/MS is 2.5 μl while the 6-plex screening uses 5 μl.

### Occurrence and discussion of specific mycotoxins

#### Aflatoxins

The 6-plex immunoassay data suggests the presence of AFB_1_ in several beer samples with concentrations ranging from 0.1 to 3.7 μg/L (S5 Table). In particular imperial stouts (Table H in S5 Table), as well as some other dark beers, showed AFB_1_ contaminations. However, when analysed by LC-MS/MS, none out of the 28 selected AFB_1_ suspected beers could be confirmed. Since the AFB_1_ immunoassay showed almost no cross-reaction with AFB_2_, AFG_1_ or AFG_2_ [51], contamination with these metabolites was highly unlikely. A possible explanation for the screening results could be the presence of the structurally related sterigmatocystin (STC), whose presence in beer has been reported [53]. However, additional testing showed that STC had no cross-reactivity in the AFB_1_ assay. Further research is needed to elucidate the origin of the observed suspect screening results, but it seems plausible that matrix effects yielded the false positive results in the immunoassay for these beers. On the other hand, LC-MS/MS analysis revealed 5 beers positive for AFs (Table 4) that were not screened suspect with the 6-plex immunoassay. The 8 times dilution of the sample in the immunoassay may be the reason for this, compared to a 2 times dilution in the LC-MS/MS analysis. From those 5 beers, 4 were African traditional beers and one a pale lager from Zimbabwe. All positive beers were contaminated with AFB_1_ (0.1–1.2 μg/L) and three of them also contained AFB_2_ (0.1–0.2 μg/L). In one traditional beer also AFM_1_ was detected. This may indicate the use of milk or milk derived products in this particular beer. Milk products can be used in certain beer styles (e.g. whey in milk stouts) and therefore can be a source of AFM_1_ contamination. However, nowadays milk stouts are often produced by the addition of lactose to beer [5]. In this particular sample, being home-brewed, the chance of cross-contamination from other sources (which is common in African domestic brewing) may provide a plausible explanation. AFG_1_ or AFG_2_ were not detected in any beer sample. Contaminations of AFs in African traditional beer have been previously reported [19, 20], while AF contaminations in European beers is rarely reported [18]. Occurrence of AFs in beer is of the highest toxicological concern and therefore consumption should be avoided at any time, considering that the IARC classified aflatoxins as carcinogenic to humans (Group 1) [54].

**Table 4:** Beer samples with confirmed aflatoxin contaminations (μg/L).

Combined Style	Sample Number	Craft	Country	%ABV	Screening 6-plex immunoassay (n = 3)			Confirmatory analysis LC-MS/MS (n = 1)		
					average	range	SD	AFB_1_	AFB_2_	AFM_1_
African Traditional	421	yes	South Africa	?	nd	nd	nd	0.1	<LOD	<LOD
African Traditional	423	yes	South Africa	?	nd	nd	nd	1.2^1^	0.2	0.1
African Traditional	429	yes	South Africa	?	nd	nd	nd	1.0	0.1	<LOD
African Traditional	452	yes	South Africa	?	nd	nd	nd	0.2	0.1	<LOD
Pale Lager	280	no	Zimbabwe	5	nd	nd	nd	0.2	<LOD	<LOD

n = number of replicates, %ABV = percentage alcohol by volume, SD = standard deviation, nd = not detected,

^1^ Value is above the upper quantification limit,

^?^ = %ABV unknown, LOD = limit of detection, AFB_1_ (aflatoxin B_1_), AFB_2_ (aflatoxin B_2_), AFM_1_ (aflatoxin M1)

#### T-2 and HT-2 toxins

We obtained signals for T-2/HT-2 in many beer samples in the 6-plex immunoassay (S5 Table). However, only in 3 out of the 31 T-2/HT-2 suspected beer samples (and 4 out of all 100 beers submitted for confirmatory analysis) the presence of T-2 or HT-2 toxins was confirmed by LC-MS/MS (Table 5). The highest values being 2.3 μg/L and 3.4 μg/L respectively. These values are lower than those recently found by Rodríguez-Carrasco et al [22]. In their survey 14 (out of 154) samples contained HT-2, all with levels between 24.2–38.2 μg/L. All those samples, from 2013, came from Germany and were from the wheat beer style. In our case, the presence of T-2 and HT-2 (as determined by LC-MS/MS) seemed not style nor country/region dependent. Our results suggest that several suspect immunoassay screening results may indicate either false positives or possible modified forms of T-2 and/or HT-2. Regular consumption of these beers will not lead to exceedance of the TDI of 0.1 μg/ kg BW for the sum of T-2 and HT-2 [55] (14 μg/L for a person of 70 kg BW drinking one 0.5 L bottle of beer per day). Beer sample 356, an imperial stout, had the highest T-2/HT-2 contamination (57 μg/L) in the 6-plex immunoassay, but this was not confirmed by LC-MS/MS. In preliminary follow-up research, we analysed this sample using high resolution LC-MS/MS analysis and found indications that two forms of HT-2 glycosides may be present in this beer sample (results not shown). It has been reported previously [56] that HT-2 glycosides were present in wheat. Due to the lack of proper standards, quantification of these HT-2 glycosides was not possible. For the same reason the cross-reaction of HT-2 glycosides in the 6-plex immunoassay could not be determined, making it impossible to verify if the T-2/HT-2 suspect results are based on the presence of glycosides or other possible conjugated forms. The metabolites T-2 triol and T-2 tetraol were not detected in any beer sample and therefore not the cause of the suspect screening results.

**Table 5:** Beer samples with confirmed T-2 and HT-2 contaminations (μg/L).

Combined Style	Sample Number	Craft	Country	%ABV	Screening 6-plex immunoassay (n = 3)			Confirmatory analysis LC-MS/MS (n = 1)	
					average	range	SD	T-2	HT-2
Stout	138	no	Czech Republic	10.5	nd	nd	nd	<LOQ	<LOD
Sour Ale	291	yes	Belgium	5	12.9	12.7–13.1	0,2	2.3	<LOD
Strong Pale Ale	382	yes	Belgium	10	1.2	0.9–1.6	0,4	<LOD	3.4
Pale Lager	698	no	Poland	5.2	0.3	0.1–0.4	0,1	0.7	<LOD

n = number of replicates, %ABV = percentage alcohol by volume, SD = standard deviation, nd = not detected, LOD = limit of detection, LOQ = limit of quantification, T-2 (T-2 toxin), HT-2 (HT-2 toxin)

#### Zearalenone

For ZEN, concentrations up to 5.6 μg/L were found in the 6-plex immunoassay (S5 Table). From the 3 selected ZEN suspect beers, 1 was confirmed as positive when analysed further by the LC-MS/MS method. LC-MS/MS analysis of selected negative immunoassay samples revealed 6 additional ZEN contaminations, although 5 out of 6 results were below the LOQ of 0.3 μg/L (Table 6). Also β-ZEL was found in 4 samples, in all cases below the LOQ of 2 μg/L and in two cases co-occurring with ZEN. Z14S was detected in 12 beer samples in Table 6. From those samples, 3 were above the LOQ (0.5 μg/L) while 9 samples had concentrations between the LOQ and LOD. Z14S was not found in African traditional beers. Z14S co-occurred with ZEN four times and in 2 beer samples ZEN, β-ZEL and Z14S co-occurred. In a previous survey, including conjugated mycotoxins, Z14S was not detected in beer [57]. α-ZEL, α-ZELG, β-ZELG and Z14G were not detected in any beer sample. ZEN was previously detected in high concentrations (up to 426 μg/L) in African traditional beers [20]. Using immunoassays, Bauer et al [25] and Kuzdraliński et al [24] both detected ZEN in beers (concentrations up to 2.0 μg/L), but these were not confirmed by instrumental analysis. Therefore it remains unclear whether ZEN metabolites were contributing to the ZEN values reported. The ZEN mAb in our 6-plex screening assay showed no cross-reactions to Z14S. None of the contaminated samples in our survey would lead to exceedance of the TDI for ZEN, set by EFSA at 0.25 μg/kg BW [58], under normal circumstances (35 μg/L for a person of 70 kg BW drinking one 0.5 L bottle of beer per day). This also counts if we add up the sum of all ZEN metabolites in a single sample. Therefore ZEN is not a major risk factor in the confirmed beers.

**Table 6:** Beer samples with confirmed ZEN and ZEN metabolite contaminations (μg/L).

Combined Style	Sample Number	Craft	Country	%ABV	Screening 6-plex immunoassay (n = 3)			Confirmatory analysis LC-MS/MS (n = 1)		
					average	range	SD	ZEN	β-ZEL	Z14S
African traditional	407	yes	South Africa	4	nd	nd	nd	<LOD	<LOQ	<LOD
African traditional	416	yes	South Africa	?	nd	nd	nd	<LOD	<LOQ	<LOD
African traditional	417	yes	South Africa	?	nd	nd	nd	<LOQ	<LOD	<LOD
African traditional	430	yes	South Africa	?	nd	nd	nd	<LOQ	<LOD	<LOD
Bock	238	no	Poland	10	nd	nd	nd	<LOQ	<LOD	0.7
Dark Lager	124	no	Czech Republic	3.8	nd	nd	nd	<LOQ	<LOQ	0.5
Dark Lager	132	no	Czech Republic	4.7	nd	nd	nd	<LOD	<LOD	<LOQ
Imperial Stout	183	yes	USA	15	nd	nd	nd	0.3	<LOQ	0.5
Imperial Stout	631	yes	Norway	14	nd	nd	nd	<LOQ	<LOD	<LOQ
Imperial Stout	644	yes	Netherlands	11	nd	nd	nd	<LOD	<LOD	<LOQ
Imperial Stout	771	no	Poland	8	nd	nd	nd	<LOD	<LOD	<LOQ
Non/Low Alcohol	121	no	Czech Republic	0	0.5	0.4–0.6	0.1	<LOD	<LOD	<LOQ
Pale Ale	97	yes	Netherlands	5.5	nd	nd	nd	<LOD	<LOD	<LOQ
Pale Lager	698	no	Poland	5.2	nd	nd	nd	<LOD	<LOD	<LOQ
Stout	707	yes	Denmark	7	nd	nd	nd	<LOD	<LOD	<LOQ
Strong Pale Ale	768	yes	Norway	10	nd	nd	nd	<LOD	<LOD	<LOQ

n = number of replicates, %ABV = percentage alcohol by volume, nd = not detected,

^?^ = %ABV unknown, LOD = limit of detection, LOQ = limit of quantification, ZEN (zearalenone), β-ZEL (β-zearalenol), zearalenone 14-sulfate (Z14S)

#### Ochratoxin A

The 6-plex immunoassay assay showed several OTA suspect samples with indicative levels ranging from 0.1–1.6 μg/L (S5 Table). From the 25 selected OTA suspect samples, 6 samples were confirmed positive by LC-MS/MS and 5 samples had OTA concentrations ranging from 0.3–0.6 μg/L (Table 7). Remarkably, from these confirmed OTA positives, 4 beers were from the same Norwegian brewery. The other beers confirmed positive were from England, but originated from different breweries. Note that sampling of all these positive craft beers had occurred in the same year (2011) and at the same craft beer festival. The OTA contaminations found in our survey were in beers from European origin. They were slightly higher than those previously found (in European beers) by Visconti et al [28] and Bertuzzi et al [29] but considerably lower than the contaminations found by Odhav en Naicker [20] in African traditional beers. OTB was not detected in any beer sample. OTA is of high toxicological concern, since it is possibly carcinogenic to humans (Group 2B) [59]. In 2006, EFSA established a Tolerable Weekly Intake (TWI) for OTA of 120 ng/kg BW per week which can be translated to an average of 17 ng/kg BW per day [60]. The beers in our survey confirmed positive for OTA do not surpass this derived TDI under normal circumstances (2.4 μg/L for a person of 70 kg BW drinking one 0.5 L bottle of beer per day).

**Table 7:** Beer samples with confirmed OTA contaminations (μg/L).

Combined Style	Sample Number	Craft	Country	%ABV	Screening 6-plex immunoassay (n = 3)			Confirmatory analysis LC-MS/MS (n = 1)
					average	range	SD	OTA
Bock	325	yes	Norway	8.5	0.8	0.8	<0,1	0.6
Dark Ale	361	yes	Norway	4.5	0.4	0.3–0.4	0,1	0.3
Double India Pale Ale	330	yes	Norway	10	0.7	0.7–0.8	0,1	0.5
India Pale Ale	300	yes	England	6	0.3	0.3–0.4	<0,1	<LOQ
Pale Ale	380	yes	Norway	6	0.4	0.3–0.4	0,1	0.4
Strong Pale Ale	353	yes	England	11	0.6	0.4–0.7	0,1	0.4

n = number of replicates, %ABV = percentage alcohol by volume, LOQ = limit of quantification, OTA (ochratoxin A)

#### Fumonisins

From previous research it is known that the 6-plex immunoassay overestimates the fumonisin content [51]. As a result, several false suspects became apparent when we compared the FBs immunoassay data (S5 Table) with the LC-MS/MS data. Higher false suspect concentrations were typically found in darker style beers (e.g. imperial stouts, dark lagers and ales). Confirmatory analysis showed that pale lagers contaminated with FB_1_ were mainly from Spain and Italy (Table 8). In some countries, mostly for economic reasons, pale lagers often contain other cereals besides barley. The declared information on the label of some of these supermarket beers purchased in Spain and Italy, revealed that they contain maize as an adjunct. In fact, Italian and Spanish beers brewed with higher amounts of barley (or 100% barley) are often considered specialty beers in these countries. The use of maize in pale lagers (or any other beer) increases the risk of contamination with FBs. These data may suggest a Mediterranean trend. However, in a preliminary screening of available Greek commercial and craft beers in 2015, we did not find any pale lager that contained FBs (results not published). The highest LC-MS/MS FB_1_ contamination detected in our survey was for an Italian pale lager (51 μg/L), followed by a Spanish non-alcoholic beer that contained (28 μg/L). In only 4 beer samples we were able to detect FB_3_ and in 2 beer samples this was above the LOQ (1 μg/L). FB_2_ was not detected in any beer sample. Fumonisin contaminations of Italian beers (30 μg/L) and Spanish beers (85 μg/L) were reported previously [29, 39]. Besides pale lagers, mainly African traditional home-brews were prone to FB_1_ contamination. The highest contamination in that category was 36 μg/L with another two beers close to this contamination level (30 and 28 μg/L respectively) (Table 8). There are agro-ecological and cultural reasons for this. First, most of Africa is hot and humid thus ideal for *Fusarium* infection and growth. Further, Shepard et al [35] have shown that in the Eastern Cape, the best maize is selected for cooking while the mouldy maize is then used for brewing beer. It is believed that infected maize adds a desirable taste to the final beer. Beers from two Spanish breweries were sampled again approximately 2 years later, and analysed only for FBs using LC-MS/MS (S6 Table). Like the previous results, the cheapest pale lagers (from brewery #2) had the highest FB_1_ (56 μg/L) and total FB contamination (69 μg/L). Beers from brewery #1, showed lower FB contaminations this time (14 and 17 μg/L respectively) at the sampling two years later. Besides FB_1_, all beers contained FB_2_ and FB_3_ in this reassessment. With the FB concentrations found in our survey, the TDI is not easily exceeded. EFSA has set a group TDI of 2 μg/kg BW per day (sum of FB_1_, FB_2_ and FB_3_) [61]. If we take into account an average body weight of 70 kilogram for an adult [62], then a person would need to drink more than 2 litres per day of the highest contaminated beer (69 μg/L) before reaching the TDI [61]. At that consumption level, alcohol intake is definitely a more serious risk. Nevertheless, daily exposure to FB_1_ through beer should be avoided as much as possible, since consumers may be exposed to other dietary sources of FBs as well. FBs are of high toxicological concern, since they are possibly carcinogenic to humans (Group 2B) [59]. The incidence of human oesophageal cancer and the occurrence of *Fusarium verticillioides* (and its mycotoxins FBs), has been associated with regions where corn is produced and consumed as staple food [63]. Franceschi et al [64] reported significant associations, in males, between maize consumption and oral cancer in northern Italy. In a case-control study, 80% of the patients diagnosed with oesophageal cancer indicated to be regular consumers of African traditional beers. Based on these findings, Segal et al [65] concluded that the consumption of these African traditional beers was a major risk factor. However, the African traditional beers analysed in our survey, did not have that extreme FBs contaminations compared to those previously reported in literature [19, 35].

**Table 8:** Beer samples with confirmed FB contaminations (μg/L).

Combined Style	Sample Number	Craft	Country	%ABV	Screening 6-plex immunoassay (n = 3)			Confirmatory analysis LC-MS/MS (n = 1)	
					average	range	SD	FB_1_	FB_3_
African Traditional	272	yes	South Africa	?	27	24–31	4	3	<LOD
African Traditional	278	yes	Zimbabwe	6	376	357–393	18	28^1^	<LOQ
African Traditional	407	yes	South Africa	4	30	28–31	1	11	<LOD
African Traditional	416	yes	South Africa	?	5	3–7	2	4	<LOD
African Traditional	417	yes	South Africa	?	11	10–12	1	4	<LOD
African Traditional	420	yes	South Africa	?	10	8–11	1	5	<LOD
African Traditional	421	yes	South Africa	?	24	20–27	4	7	<LOD
African Traditional	423	yes	South Africa	?	84	82–88	3	36^1^	<LOD
African Traditional	427	yes	South Africa	?	15	13–16	2	3	<LOD
African Traditional	428	yes	South Africa	?	15	14–16	1	2	<LOD
African Traditional	429	yes	South Africa	?	25	25–26	1	30^1^	<LOD
African Traditional	430	yes	South Africa	?	16	16–17	1	3	<LOD
African Traditional	451	yes	South Africa	?	28	27–29	1	27^1^	<LOD
African Traditional	452	yes	South Africa	?	33	31–35	2	13	<LOD
Non/Low Alcohol	398	no	Spain	0	34	32–35	1	28^1^	<LOD
Pale Ale	217	no	Poland	5.7	7	3–9	3	12	<LOD
Pale Lager	48	no	Germany	5	20	19–21	1	20	<LOD
Pale Lager	280	no	Zimbabwe	5	11	10–13	2	11	<LOQ
Pale Lager	386	no	Spain	4.8	7	6–8	1	25^1^	<LOQ
Pale Lager	388	no	Spain	5.4	22	20–24	2	16	<LOQ
Pale Lager	399	no	Spain	5.5	36	32–39	4	9	<LOD
Pale Lager	488	no	Italy	4.7	71	64–76	6	51^1^	<LOD
Pale Lager	493	no	Italy	4.7	59	58–63	3	15	<LOD
Pale Lager	498	no	Italy	4.5	56	54–59	2	11	<LOD

n = number of replicates, %ABV = percentage alcohol by volume, nd = not detected,

^1^ Value is above the upper quantification limit,

^?^ = %ABV unknown, LOD = limit of detection, LOQ = limit of quantification, FB_1_ (fumonisin B_1_), FB_3_ (fumonisin B_3_)

#### Type B trichothecenes: DON, D3G, ADONs and NIV

**Fig 3:**
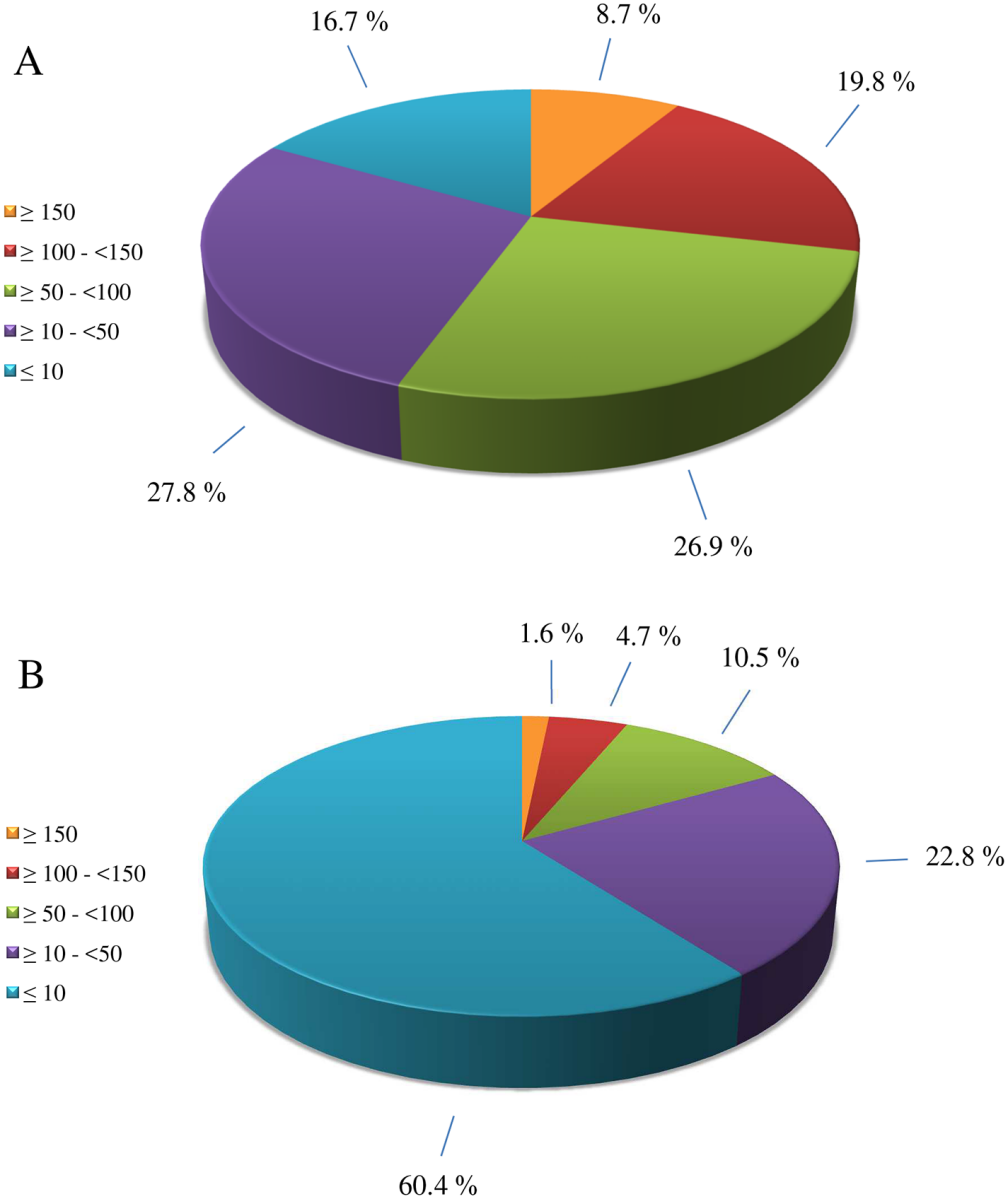
Occurrence of different DON+D3G contamination levels (μg/L) in all beer styles (A) and in the imperial stout beer style (B), based on the 6-plex screening results and the percentage of total beers.

**Fig 4:**
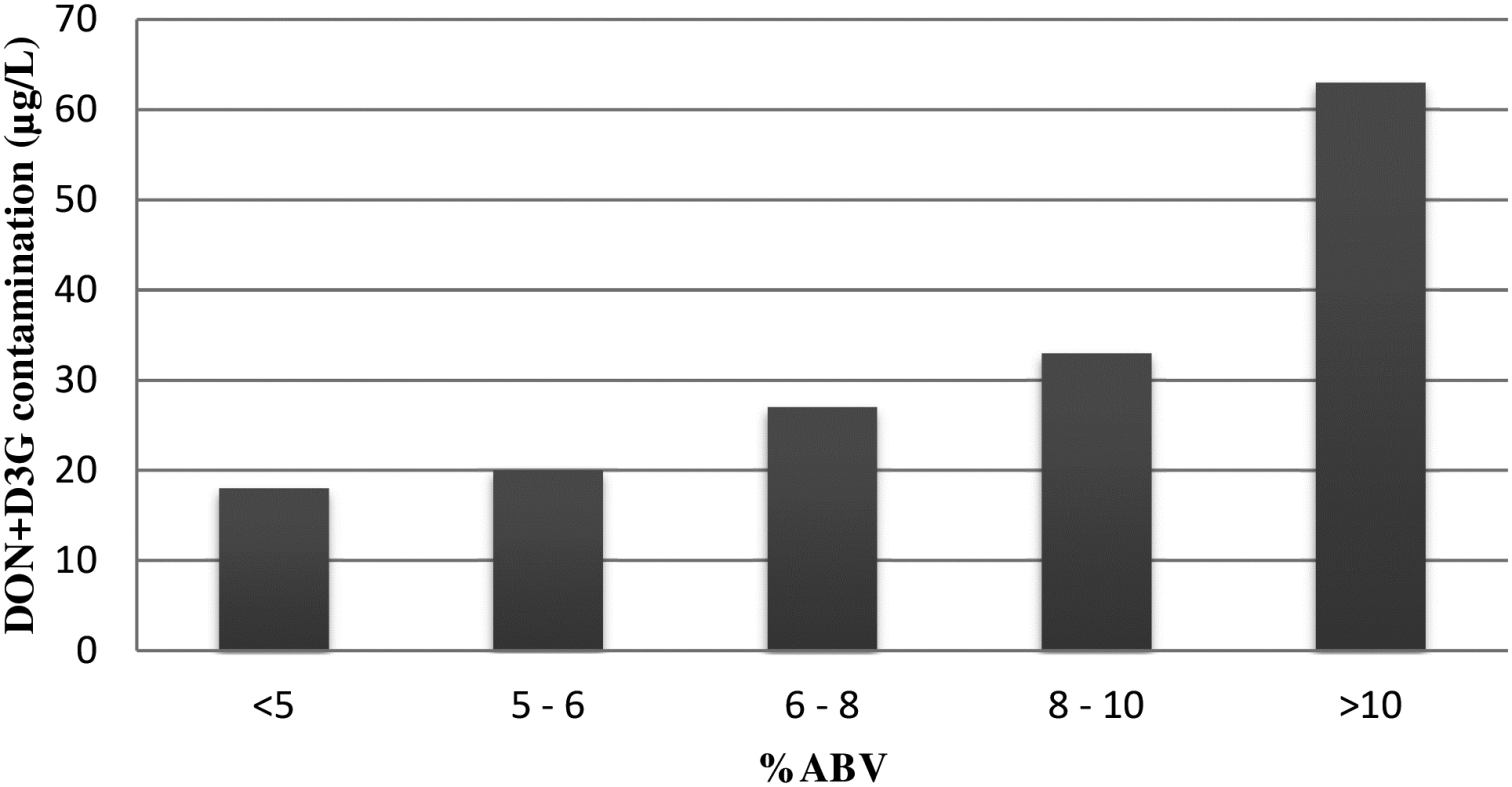
Correlation of DON+D3G contaminations (μg/L) relative to the %ABV based on the 6-plex screening results.

The 6-plex immunoassay reports results for the sum of DON and D3G (S5 Table). This is beneficial since previous research seems to indicate [66–69] that D3G is of toxic relevance. Recently a request was made to EFSA for a scientific opinion on the risks for animal and human health related to the presence of deoxynivalenol, its metabolites and masked deoxynivalenol (D3G) in food and feed [70]. Therefore, it is plausible that D3G will be added to the total DON group (of DON and its acetylated derivatives) for risk assessment. The majority of the beers screened (60%) had contamination levels below 10 μg/L of DON+D3G, while beers with contaminations above 100 μg/L occurred less frequent (6%) (Fig 3). From the 406 beers that have DON+D3G contaminations above 10 μg/L, 73% were craft beers and these had a higher average contaminations (63 μg/L) compared to industrial produced beers (39 μg/L). The popular craft beer style imperial stout did not follow this trend. Only 17% of all imperial stouts screened had DON+D3G contamination levels below 10 μg/L, while 29% had DON+D3G contaminations above 100 μg/L (Fig 3). The highest overall DON+D3G contaminations were present in imperial stout, eisbock and stout (475, 308 and 169 μg/L, respectively) beers. The highest average group DON+D3G contaminations, based on beer style, were imperial stout, eisbock and African traditional with 86, 81 and 65 μg/L respectively, while the saison, pale lager and non/low alcohol beer styles had the lowest average contaminations (19, 23 and 23 μg/L, respectively) (S7 Table). The highest contamination incidences were found in eisbock, imperial stout and dark lager (83, 83 and 68%, respectively) (S7 Table), while the lowest contamination incidences were found in the sour ales, saison and pale lager beer styles (7, 8 and 13% respectively (S7 Table). The screening results revealed a clear correlation between the alcohol content (%ABV) and the DON+D3G contamination (Fig 4). The same positive correlation was reported in previous mycotoxin beer surveys [40, 43, 44]. For high %ABV beers, a higher input of grains is needed to deliver the fermentable sugars and with that comes a higher risk of mycotoxin contamination. LC-MS/MS data showed that, within the selected 100 beers for confirmation, 26 beers were contaminated with only DON above the corresponding LOD and 13 beers with only D3G. In contrast, in 38 beers both DON and D3G were detected. In 19 beers the concentration of D3G was higher than that of DON (Table 9). Varga et al [44] previously reported molar D3G/DON ratios (corrected for molecular mass) between 0.11 and 1.25 with an average of 0.56. In our survey, the molar D3G/DON ratios ranged from 0.10 to 2.60 with an average of 0.79. The highest ratio observed was for a pale lager from Poland. Generally, in this survey, beers having a D3G/DON ratio higher than 0.60, are almost all craft beers. DON was also present in African traditional beers, but in these beers no D3G contaminations were observed. The absence of D3G in these beers, may indicate that sorghum malt, often used in these traditional beer styles [71], does not have the potential to conjugate DON to D3G. Like the screening assay, LC-MS/MS analysis showed that beer sample 183 had the highest contamination for both DON and D3G with contaminations of 412 and 619 μg/L respectively. Since these concentrations were more than 2 times higher than the standard addition, we decided to reanalyze this particular beer sample following a 10 times dilution. Reanalysis showed that it contained 309 μg/L of DON and 535 μg/L of D3G. These concentrations reconfirmed that this imperial stout had the most extreme contamination in the entire survey. Recently, Piacentini et al [36] surveyed Brazilian craft beers using liquid chromatography with fluorescence detection and found beers with high DON contaminations (17 samples, range 127–501 μg/L). Unfortunately the surveyed beer styles were not further defined than ales and lagers. Previously, DON concentrations as high as 501 μg/L had only been reported in African traditional beers [34, 45]. The LC-MS/MS method used was not able to distinguish between 3ADON and 15ADON (ADONs) and therefore the ADON contaminations found should be considered as the sum of both (Table 9). In 5 African traditional beers ADONs were detected and in 3 of those also NIV was present. All these mycotoxin levels were below the LOQ (10 μg/L for ADONs and 5 μg/L for NIV). ADONs were also detected in one pale ale (Poland) and 2 pale lagers (Italy), again all below the LOQ. In one Dutch pale ale, NIV was detected at a concentration of 21 μg/L. These results indicate that NIV and ADONs are not frequent contaminants of beers, with the exception of African traditional beers (5 out of 14). Previously, Kostelanska et al [43] found ADONs concentrations as high as 25 μg/L and described them as common contaminants present in 50% of 176 beers that were analysed. On the other hand, Bertuzzi et al [29] could not find any ADONs in 106 beer samples analysed while Varga et al [44] did not find any 3ADON in 374 beer samples surveyed.

**Table 9:** Beer samples with confirmed trichothecene B contaminations (μg/L).

Combined Style	Sample Number	Craft	Country	%ABV	Screening 6-plex immunoassay (n = 3)			Confirmatory analysis LC-MS/MS (n = 1)			
					Average	Range	SD	DON	D3G	ADONs	NIV
African Traditional	272	yes	South Africa	?	nd	nd	nd	10	<LOD	<LOD	<LOD
African Traditional	407	yes	South Africa	4	nd	nd	nd	16	<LOD	<LOD	<LOD
African Traditional	416	yes	South Africa	?	nd	nd	nd	68	<LOD	<LOD	<LOD
African Traditional	417	yes	South Africa	?	107	94–118	12	139	<LOD	<LOQ	<LOD
African Traditional	420	yes	South Africa	?	107	96–118	11	133	<LOD	<LOQ	9
African Traditional	421	yes	South Africa	?	nd	nd	nd	<LOQ	<LOD	<LOD	<LOD
African Traditional	423	yes	South Africa	?	10	4–22	10	10	<LOD	<LOD	<LOD
African Traditional	427	yes	South Africa	?	91	83–104	12	140	<LOD	<LOQ	9
African Traditional	428	yes	South Africa	?	43	36–51	8	99	<LOD	<LOQ	8
African Traditional	430	yes	South Africa	?	57	46–75	15	121	<LOD	<LOQ	<LOD
Bock	238	no	Poland	10	97	90–102	6	64	97	<LOD	<LOD
Bock	325	yes	Norway	8.5	53	47–59	6	40	23	<LOD	<LOD
Dark Lager	124	no	Czech Republic	3.8	106	102–112	5	41	68^1^	<LOD	<LOD
Dark Lager	132	no	Czech Republic	4.7	17	8–33	14	24	36	<LOD	<LOD
Double India Pale Ale	330	yes	Norway	10	78	73–80	4	67	48	<LOD	<LOD
Eisbock	351	yes	Belgium	39	41	36–48	6	32	32	<LOD	<LOD
Fruit/Vegetable/Spice	57	no	Germany	2.5	99	93–103	5	<LOQ	<LOD	<LOD	<LOD
Imperial Stout	11	yes	Norway	15.5	140	138–143	3	104	56^1^	<LOD	<LOD
Imperial Stout	183	yes	USA	15	475	429–516	45	412^2^	619^1^	<LOD	<LOD
Imperial Stout	259	yes	Norway	11	22	15–28	7	23	11	<LOD	<LOD
Imperial Stout	317	yes	Norway	9	62	60–66	3	116	69^1^	<LOD	<LOD
Imperial Stout	356	yes	Netherlands	11	45	34–55	11	28	15	<LOD	<LOD
Imperial Stout	581	yes	USA	9.6	167	166–169	2	146	88^2^	<LOD	<LOD
Imperial Stout	589	yes	USA	9.6	85	76–99	12	43	22	<LOD	<LOD
Imperial Stout	596	yes	USA	13	280	251–322	37	73	113^1^	<LOD	<LOD
Imperial Stout	607	yes	Denmark	10.9	152	131–170	20	14	27	<LOD	<LOD
Imperial Stout	631	yes	Norway	14	125	108–141	17	182	43	<LOD	<LOD
Imperial Stout	644	yes	Netherlands	11	21	16–26	5	<LOD	13	<LOD	<LOD
Imperial Stout	674	yes	USA	10.5	56	53–61	4	26	61^1^	<LOD	<LOD
Imperial Stout	678	yes	Denmark	13	34	31–38	3	<LOD	20	<LOD	<LOD
Imperial Stout	748	yes	USA	11	101	97–105	4	32	45	<LOD	<LOD
Imperial Stout	764	yes	USA	11	21	17–25	3	<LOD	49	<LOD	<LOD
Imperial Stout	767	yes	USA	8	2	1–3	1	<LOD	9	<LOD	<LOD
Imperial Stout	771	no	Poland	8	21	19–23	2	<LOD	39	<LOD	<LOD
Imperial Stout	780	yes	Canada	8.5	54	45–81	8	38	81^1^	<LOD	<LOD
India Pale Ale	62	yes	USA	7.1	16	7–23	9	<LOQ	12	<LOD	<LOD
India Pale Ale	449	yes	USA	7.5	5	3–10	6	16	18	<LOD	<LOD
India Pale Ale	479	yes	Belgium	7	84	80–87	4	64	12	<LOD	<LOD
Non/Low Alcohol	121	no	Czech Republic	0	36	25–60	20	16	16	<LOD	<LOD
Pale Ale	54	yes	Belgium	8	4	1–9	4	<LOQ	<LOD	<LOD	<LOD
Pale Ale	97	yes	Netherlands	5.5	92	85–99	7	40	82^1^	<LOD	21
Pale Ale	176	yes	France	6.5	nd	nd	nd	<LOD	8	<LOD	<LOD
Pale Ale	217	no	Poland	5.7	26	18–32	7	20	6	<LOQ	<LOD
Pale Ale	380	yes	Norway	6	8	2–14	7	9	<LOD	<LOD	<LOD
Pale Lager	55	no	Germany	5.2	17	13–20	3	12	22	<LOD	<LOD
Pale Lager	208	no	Poland	5	31	28–36	4	15	20	<LOD	<LOD
Pale Lager	399	no	Spain	5.5	nd	nd	nd	<LOQ	<LOD	<LOD	<LOD
Pale Lager	488	no	Italy	4.7	nd	nd	nd	<LOD	9	<LOQ	<LOD
Pale Lager	493	no	Italy	4.7	nd	nd	nd	<LOD	6	<LOQ	<LOD
Pale Lager	698	no	Poland	5.2	70	65–74	5	13	53^1^	<LOD	<LOD
Smoked	369	yes	Netherlands	11	94	70–101	17	23	14	<LOD	<LOD
Sour Ale	119	yes	Belgium	5	nd	nd	nd	13	<LOD	<LOD	<LOD
Sour Ale	310	yes	Belgium	8	25	22–27	3	29	21	<LOD	<LOD
Sour Ale	343	yes	Belgium	6	nd	nd	nd	14	<LOD	<LOD	<LOD
Sour Ale	597	yes	Italy	9	14	4–40	22	<LOD	7	<LOD	<LOD
Stout	138	no	Czech Republic	10.5	49	31–70	19	<LOQ	42	<LOD	<LOD
Stout	160	yes	Sweden	7.5	135	116–157	21	26	30	<LOD	<LOD
Stout	647	yes	USA	6.4	41	28–50	11	30	<LOD	<LOD	<LOD
Stout	707	yes	Denmark	7	66	58–75	8	<LOD	52^1^	<LOD	<LOD
Strong Dark Ale	159	yes	Belgium	8	19	0–47	nd	10	18	<LOD	<LOD
Strong Dark Ale	189	no	Belgium	10	nd	nd	nd	<LOQ	<LOD	<LOD	<LOD
Strong Dark Ale	444	yes	Belgium	10.2	40	34–43	5	25	35	<LOD	<LOD
Strong Pale Ale	5	yes	Denmark	10	50	47–54	3	26	<LOD	<LOD	<LOD
Strong Pale Ale	17	yes	Denmark	12	79	53–94	23	25	41	<LOD	<LOD
Strong Pale Ale	18	yes	Netherlands	10.5	25	18–36	9	<LOD	21	<LOD	<LOD
Strong Pale Ale	84	no	Belgium	9	nd	nd	nd	<LOQ	<LOD	<LOD	<LOD
Strong Pale Ale	150	yes	USA	11.5	76	61–89	14	<LOD	25	<LOD	<LOD
Strong Pale Ale	353	yes	England	11	nd	nd	nd	<LOQ	<LOD	<LOD	<LOD
Strong Pale Ale	382	yes	Belgium	10	32	27–38	6	32	<LOD	<LOD	<LOD
Strong Pale Ale	508	yes	England	10.2	42	36–50	7	23	21	<LOD	<LOD
Strong Pale Ale	768	yes	Norway	10	7	4–11	3	<LOD	20	<LOD	<LOD
Strong Pale Lager	460	no	Austria	14	41	32–48	8	12	17	<LOD	<LOD
Wheat	113	no	Netherlands	5	36	29–44	7	<LOQ	<LOD	<LOD	<LOD
Wheat	228	no	Germany	5.6	3	2–4	2	<LOQ	<LOD	<LOD	<LOD
Wheat	454	no	Germany	5	17	7–26	9	10	<LOD	<LOD	<LOD
Wheat	540	no	Belgium	4.9	nd	nd	nd	<LOQ	<LOD	<LOD	<LOD
Wheat	550	no	Netherlands	5	26	12–36	13	<LOQ	4	<LOD	<LOD

n = number of replicates, %ABV = percentage alcohol by volume, nd = not detected,

^1^ Value is above the upper quantification limit,

^?^ = %ABV unknown, LOD = limit of detection, LOQ = limit of quantification, DON (deoxynivalenol), D3G (deoxynivalenol-3-β-D-glucopyranoside) and ADONs (sum of 3-acetyl-DON and 15-acetyl-DON), NIV (nivalenol)

Taking into account that the DON mAb used in the 6-plex screening assay has 60% cross-reaction to D3G, we compared the 6-plex and the LC-MS/MS data. In 23 beers, both in 6-plex and LC-MS/MS, no DON+D3G was detected. From the 77 beers confirmed positive for DON and/or D3G (Table 9) by LC-MS/MS, 14 beers were negative in the 6-plex immunoassay. Concentrations for DON+D3G in these samples were generally low with the exception of African traditional beer sample 416 (68 μg/L) suggesting a beer specific interference in the immunoassay. For 27 beers, the 6-plex values for DON+D3G were below those found by LC-MS/MS analysis with an average factor of 0.7. For imperial stouts (9 beers) this factor was the same. For 32 beers, the 6-plex values for DON+D3G were above those found by LC-MS/MS. In average, the immunoassay values were a factor 2.1 higher. This value excludes beer 57, a fruit/beer mix, which showed a 24 times overestimation. After reanalysis, with both the 6-plex immunoassay and LC-MS/MS, this beer still showed the same overestimation. The addition of grapefruit juice to this beer seems to be responsible for high matrix interference in the 6-plex assay. For the imperial stouts (9 beers) within the group of 32 beers, the average 6-plex values for DON+D3G were a factor 2.3 higher when compared to LC-MS/MS. This was mainly attributed to a few extremes (Table 9).

Imperial stouts show the highest DON+D3G contaminations in our survey, and since both USA and European imperial stouts were well presented, a geographical comparison was made. In total, 52 imperial stouts from the USA and 74 imperial stouts from Europe were screened. The mean DON+D3G contaminations (based on beers with contaminations higher than 10 μg/L) were 93 μg/L and 64 μg/L respectively. This suggests that USA imperial stouts have higher DON+D3G contaminations compared to European ones. This may be attributed to malt usage. Imperial stout is a high gravity style and is mostly pitch-black because of the specific taste-defining malts used. These malts are often, but not limited to, brown malt, caramel malt, chocolate malt and roast malt. This may suggest that these colored malts are responsible for the high DON+D3G contributions, since the strong pale lagers (e.g. barley wines) seem to suffer less of high DON+D3G contaminations compared to the imperial stouts. When comparing identical styles divided by lighter and darker colors (pale vs. dark) (S3 Fig), dark lagers clearly have higher DON+D3G contaminations compared to pale lagers. Dark ales tend to have higher contaminations (higher than 50 μg/L) compared to pale ales, but pale ales have more contaminations higher than 25 μg/L. Comparison of strong pale lagers and strong dark lagers shows minor differences. These comparisons show that for some beer styles higher contaminations can be associated to beer color.

The DON+D3G contaminations in 27 beers from this survey, are equal to, or exceed the TDI of 1μg/kg BW for DON (140 μg/L for a person of 70 kg BW drinking one 0.5 L bottle of beer per day). Personal risk, related to exceeding the TDI for DON, based on the DON+D3G contaminations for selected beers in this survey, is presented in Table 10. Additionally, the consumption of multiple bottles and the likely additional exposure via the daily diet (bread, pasta, breakfast cereals) further increases the risk. Unlike FBs, AFs and OTA, DON is not grouped as a (possible) carcinogenic mycotoxin. Its intake causes symptoms like vomiting, nausea, growth retardation, reproductive disorders and suppression of the immune system in humans and animals [72]. More recently DON is also believed to be active at the central nervous system level (brain) causing modified neurochemistry and neuronal activity [73].

**Table 10:** Personal risk based on the tolerable daily intake (TDI) and the DON+D3G contamination in selected beers.

Body Weight (kg)	DON+D3G concentration (LC-MS/MS)											
	Dark lager (132)^#^ Czech Republic 60 μg/L			Pale ale (97)^#^ Netherlands 122 μg/L			Imperial stout (631)^#^ Norway 225 μg/L			Imperial stout (183)^#^ USA 1031 μg/L		
	0.33 L	0.5 L	1.0 L	0.33 L	0.5 L	1.0 L	0.33 L	0.5 L	1.0 L	0.33 L	0.5 L	1.0 L
50	-	-	+	-	+	+	+	+	+	+	+	+
70*	-	-	-	-	-	+	+	+	+	+	+	+
100	-	-	-	-	-	+	-	+	+	+	+	+

- below or equals TDI,

+ above TDI,

^#^ Beers from the survey with DON+D3G contaminations as determined by LC-MS/MS

* default average BW (body weight) set by EFSA for the European adult population,

DON (deoxynivalenol), D3G (deoxynivalenol-3-β-D-glucopyranoside)

## Conclusion

To our best knowledge, this survey is the largest ever performed for the occurrence of mycotoxins in beer. It is for certain the most extensive screening for mycotoxins in craft beers to date. The applied mycotoxin 6-plex screening method facilitated fast and easy screening of 1000 global beer samples, whilst the developed beer-dedicated LC-MS/MS method proved to be very useful for quantitative confirmatory analysis. The effectiveness of the 6-plex mycotoxin immunoassay screening approach, without any sample clean-up, was hampered by matrix interferences. This occurred particularly at low concentrations and certain beer styles. It caused false suspect samples for AFB_1_, FBs, T-2/HT-2 mainly in dark beer styles. For type B trichothecenes the chosen approach lead to over- and underestimations, particularly in a few imperial stouts. Therefore, a blank reference beer for imperial stouts is desired in the 6-plex assay. It will help to improve mycotoxin determination in this complex beer style. But still, even then large matrix background variations can be expected. Older recipes just contain malts while newer recipes show the addition of coffee, cacao and other adjuncts. For a further reduction of over- and underestimations, a suitable clean-up procedure may be considered for future 6-plex screening of mycotoxins in beer. Additionally, averaging data from replicates in the LC-MS/MS standard addition method may lead to a better quantitative comparison. Furthermore, the use of logarithmic dose-response curves in the 6-plex immunoassay, compared to a narrow linear range for the standard additions used in LC-MS/MS, will always contribute to a less accurate quantification in the 6-plex immunoassay.

Until now, there are no Maximum Levels set for the occurrence of mycotoxins in beer. We agree with the conclusion drawn previously by Varga et al [44] about their extensive DON survey in beer, stating that setting maximum levels for DON and its metabolites in beer helps to protect beer drinkers from consuming highly contaminated beers. Based on beer samples in the presented survey, exceeding the TDI for DON, future research should additionally focus on malts and/or grains used for high gravity beer styles like imperial stout. It seems that the darker malts and/or roasted malts in imperial stouts, combined with the high gravity, contribute to the high DON+D3G levels. Color comparison of similar styles, with pale and dark varieties, partially supports this hypothesis. Current malt certificates are often lacking information, therefore, it is suggested that malts used in high gravity beer styles should be analysed in more detail, especially for DON+D3G. With that detailed information brewers should be able to judge until what gravity the malts are safe to use. It is proposed that stricter maximum mycotoxin levels, or better specified levels, are applied for malts that are used for brewing high gravity beers. Furthermore, small craft breweries should consider the implementation of cheap, reliable, easy and fast on-site mycotoxin assays to control the purchased malts and adjuncts as well as their final products. In the end, on-site mycotoxin testing may not always be feasible for small starting breweries. Therefore they should be able to rely on the proper control of the purchased malts. With the craft beer market consistently expanding, and with many craft breweries producing imperial stouts, quality control management seems a necessary step.

## Supporting information

S1 Text LC-MS/MS conditions. (PDF)

S1 Fig Multi-mycotoxin dose-response curves in dark ale. (PDF)

S2 Fig A) Global beer samples surveyed by region B) European beer samples surveyed by country.
(PDF)

S3 Fig Comparison of DON+D3G contaminations in similar beer styles. (PDF)

S1 Table Mycotoxin specific MS/MS settings and standard addition levels. (PDF)

S2 Table Intra- and interday precision of the 6-plex assay. (PDF)

S3 Table Intra- and interday IC50s based on multi-mycotoxin dose-response curves. (PDF)

S4 Table Intra- and interday determinations of mycotoxin concentrations using the 6-plex assay. (PDF)

S5 Table 6-plex immunoassay screening data for A) African traditional beers B) bock beers C) dark ale beers D) dark lager beers E) double India pale ale beers F) eisbock beers G) fruit/vegetable/spice beers H) imperial stout beers I) India pale ale beers J) non/low alcohol beers K) pale ale beers L) pale lager beers M) saison beers N) smoked beers O) sour ale beers P) stout beers Q) strong dark ale beers R) strong pale ale beers S) strong pale lager T) wheat beers.
(PDF)

S6 Table Reassessment of Spanish breweries for FB contaminations by LC-MS/MS analysis. (PDF)

S7 Table DON+D3G contamination per beer style group. (PDF)
