# Supplementary material for: Mycotoxin profiling of 1000 beer samples with a special focus on craft beer
Source: PLoS One. 2017 Oct 5;12(10):e0185887. doi: 10.1371/journal.pone.0185887 (PMC5628871; doi:10.1371/journal.pone.0185887)
Supplement: S1 Text — (PDF) [file pone.0185887.s001.pdf]

LC-MS/MS conditions positive and negative mode

<u>LC conditions</u>

Injection volume: 5 µL

Column: Restek Ultra Aqueous C18, 100 x 2.1 mm ID, 3 µm particle size

Temperature: 35°C

Flow rate: 0.40 mL/min

Positive mode:

Eluent A: water containing 1% formic acid + 1 mM ammonium formate

Eluent B: methanol/water = 95/5 (*V/V*) containing 1% formic acid + 1 mM ammonium formate

Negative mode:

Eluent A: water containing 0.1% acetic acid + 5 mM ammonium acetate

Eluent B: methanol/water = 95/5 (V/V) containing 0.1% acetic acid + 5 mM ammonium acetate

Gradient:

Time (min)	%A	%B
0	100	0
1	100	0
2	50	50
3	50	50
8	0	100
10	0	100
10.5	100	0
15	100	0

MS conditions positive mode

MS system: 5500 QTrap (AB Sciex)

Generic settings:

Scan mode	ESI+
Scan type	MRM
Scheduled MRM	Yes
Polarity	positive
Target scan time	0.3 sec
Resolution Q1	unit
Resolution Q3	unit
MR pause	5 msec
Curtain gas (CUR)	40 psi
Collision gas (CAD)	medium
Temperature (TEM)	400 °C
Ion Source Gas 1 (GS1)	50 psi
Ion Source Gas 2 (GS2)	50 psi
IonSpray Voltage (IS)	4000 V
Exit Potential (EP)	10 V

MS conditions negative mode

MS system: 5500 QTrap (AB Sciex)

Generic settings:

Scan mode	ESI-
Scan type	MRM
Scheduled MRM	No
Polarity	negative
Dwell time	5 ms
Resolution Q1	unit
Resolution Q3	unit
Settling time	20 msec
MR pause	5 msec
Curtain gas (CUR)	40 psi
Collision gas (CAD)	medium
Temperature (TEM)	400 °C
Ion Source Gas 1 (GS1)	50 psi
Ion Source Gas 2 (GS2)	50 psi
IonSpray Voltage (IS)	-4000 V
Exit Potential (EP)	-10 V
