# Supplementary figures and images for: Mycotoxin profiling of 1000 beer samples with a special focus on craft beer
Source: PLoS One. 2017 Oct 5;12(10):e0185887. doi: 10.1371/journal.pone.0185887 (PMC5628871; doi:10.1371/journal.pone.0185887)

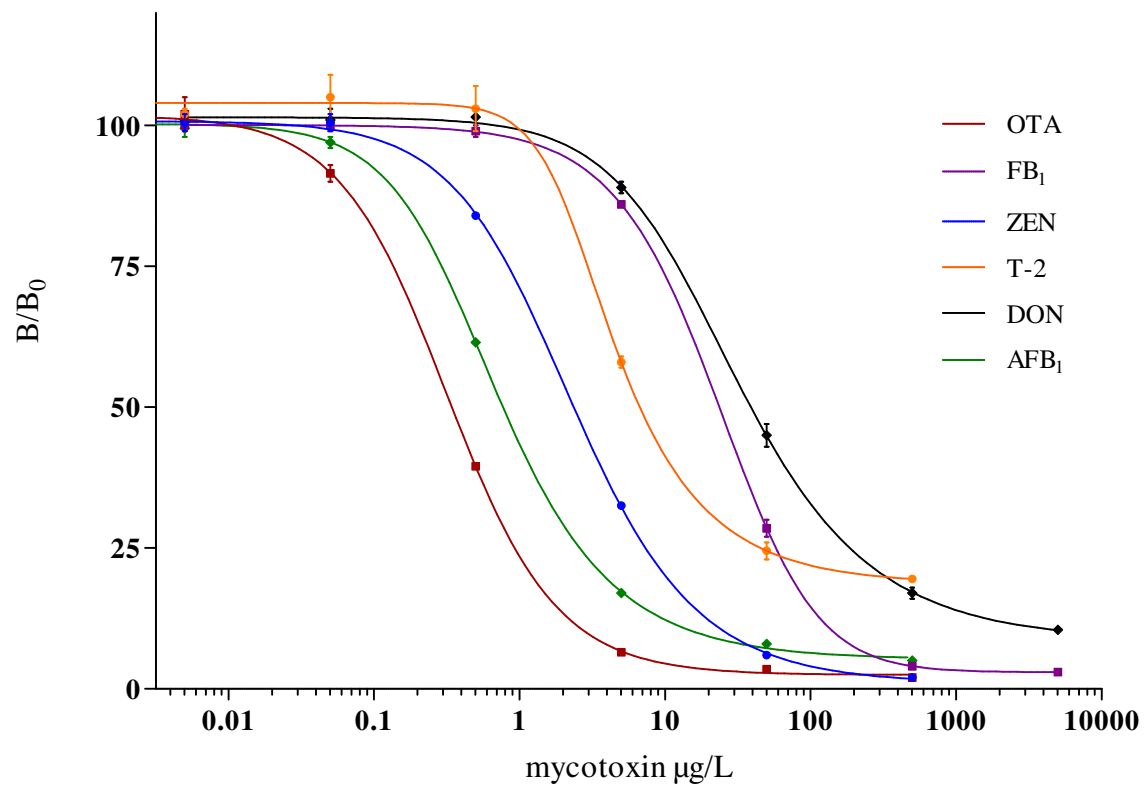

**S1 Fig.** Multi-mycotoxin dose-response curves in dark ale (n=2)

Supplement: S1 Fig — (PDF) [file pone.0185887.s002.pdf]

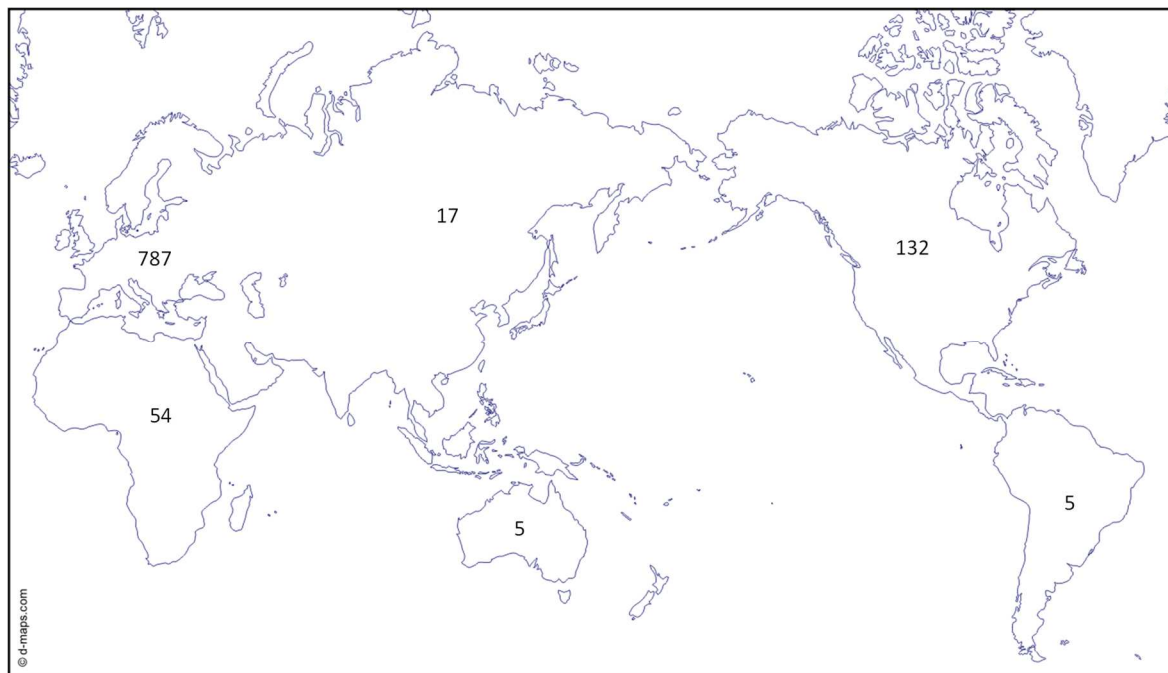

**S2 Fig. A** Global beer samples surveyed by region

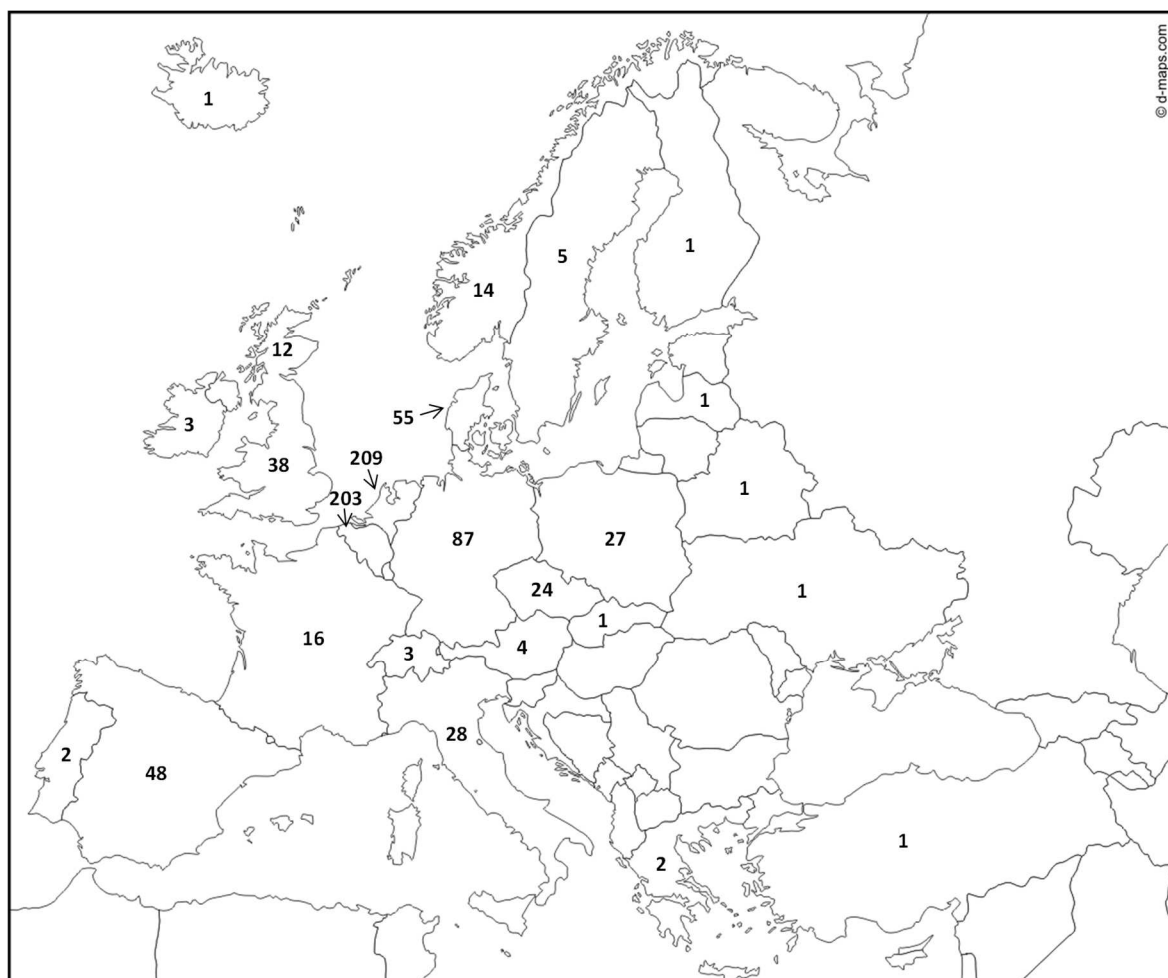

**S2 Fig. B** European beer samples surveyed by country

Supplement: S2 Fig — A) Global beer samples surveyed by region B) European beer samples surveyed by country. (PDF) [file pone.0185887.s003.pdf]
