# Supplementary material for: Mycotoxin profiling of 1000 beer samples with a special focus on craft beer
Source: PLoS One. 2017 Oct 5;12(10):e0185887. doi: 10.1371/journal.pone.0185887 (PMC5628871; doi:10.1371/journal.pone.0185887)
Supplement: S3 Fig — (PDF) [file pone.0185887.s004.pdf]

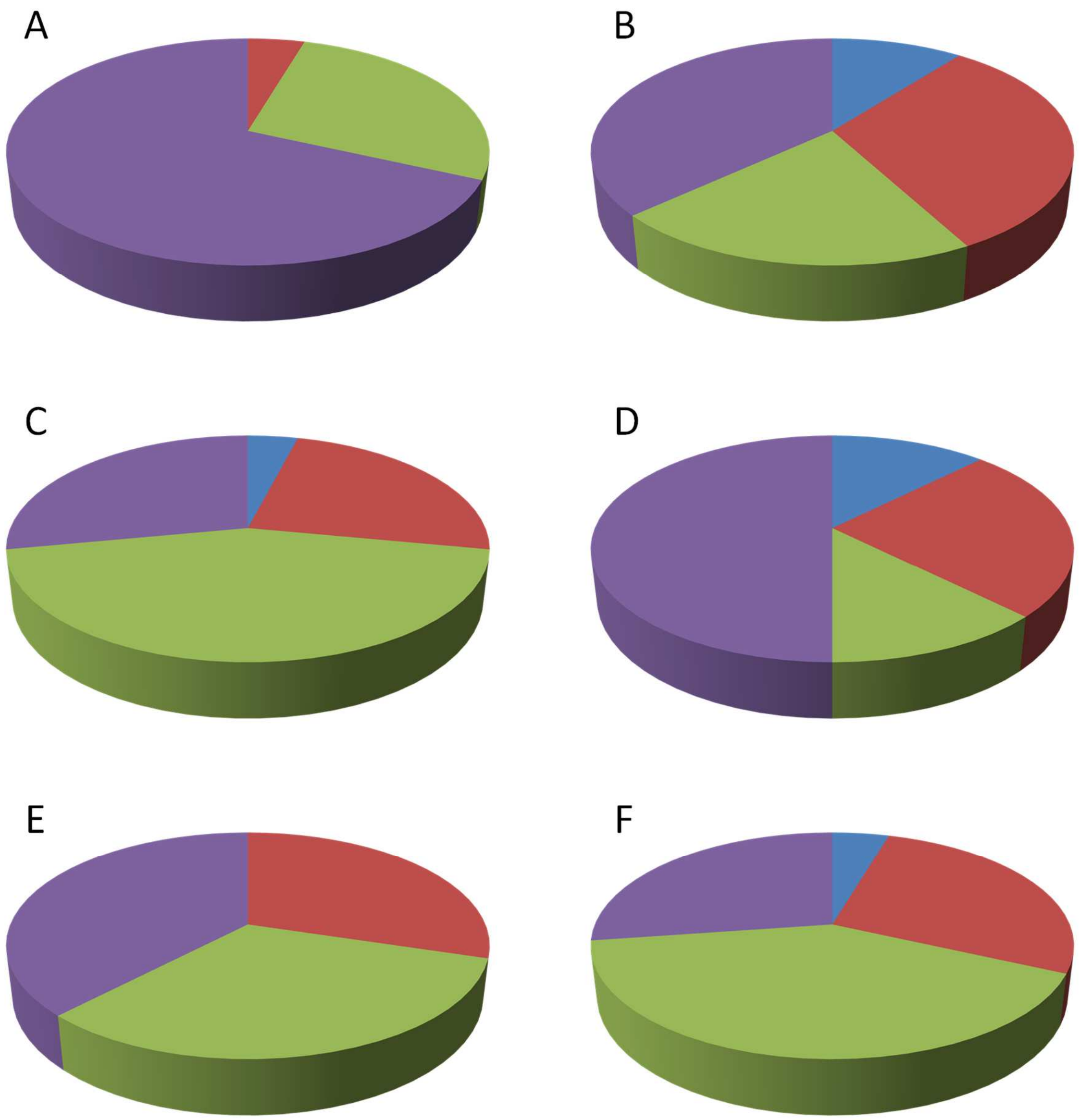

S3 Fig. Comparison of DON+D3G contaminations (■ ≥ 100, ■ ≥ 50, ■ ≥ 25 and ■ < 25 µg/L) in pale versus dark beers in similar beer styles; (A) pale lager, (B) dark lager, (C) pale ale, (D) dark ale, (E) strong pale lager and (F) strong dark lager. Measured by 6-plex immunoassay.
