# Supplementary material for: Mycotoxin profiling of 1000 beer samples with a special focus on craft beer
Source: PLoS One. 2017 Oct 5;12(10):e0185887. doi: 10.1371/journal.pone.0185887 (PMC5628871; doi:10.1371/journal.pone.0185887)
Supplement: S1 Table — (PDF) [file pone.0185887.s005.pdf]

S1 Table A. Mycotoxin specific MS/MS settings and standard addition (spike) levels for positive ionization mode

**Mycotoxin**	**Q1 (*m/z*)**	**Q3 (*m/z*)**	**RT (min)**	**DP (V)**	**EP (V)**	**CE (V)**	**CXP (V)**	**Spike (µg/L)**	**Detection range (µg/L)**
DON	297.1	248.9	4.9	51	10	15	16	100	200 - 10
		231.0		51	10	17	16		
3/15 ADON	355.9	137.1	5.9	66	10	22	10	100	200 - 10
		321.1		66	10	17	20		
AFB_1	313.0	285.2	7.7	101	10	33	16	0.5	1 - 0.05
		128.1		101	10	91	10		
AFB_2	315.1	287.2	7.6	91	10	37	18	0.5	1 - 0.05
		259.2		91	10	43	18		
AFG_1	329.0	243.2	7.2	81	10	39	14	0.25	0.5 – 0.025
		200.0		81	10	59	12		
AFG_2	331.1	313.2	7.0	106	10	35	18	0.5	1 - 0.05
		245.2		106	10	43	14		
AFM_1	328.9	272.9	6.8	61	10	33	18	0.5	1 - 0.05
		229.0		61	10	55	16		
OTA	404.0	239.0	9.00	86	10	37	16	3	6 - 0.3
		102.0		86	10	105	14		
OTB	370.1	204.9	8.5	56	10	29	14	3	6 - 0.3
		186.9		56	10	47	12		
FB_1	722.5	352.3	8.2	116	10	55	12	10	20 - 1
		334.4		116	10	57	4		
FB_2	706.4	336.3	7.6	121	10	59	8	10	20 - 1
		318.5		121	10	51	2		
FB_3	706.3	336.3	8.6	121	10	59	8	10	20 - 1
		318.5		121	10	51	2		
T-2	484.3	215.2	8.3	81	10	29	18	5	10 - 0.5
		185.1		81	10	31	11		
HT-2	441.9	263.1	7.8	86	10	19	6	10	20 - 1
		215.1		86	10	17	16		
T-2 triol	383.2	351.1	7.1	61	10	25	24	50	100 - 5
		308.9		61	10	23	20		
T-2 tetraol	316.3	281.1	4.5	51	10	13	8	100	200 - 10
		215.2		51	10	13	14		
α ZELG	483.1	321.2	7.8	61	10	13	4	5	10 - 0.5
		303.0		61	10	17	18		
β ZELG	500.1	321.0	7.0	71	10	19	22	5	10 - 0.5
		303.1		71	10	27	20		
Z14G	498	319.1	7.6	21	10	19	22	5	10 - 0.5
		301.0		21	10	31	20		

Q1 = Precursor ion

Q3 = Product ions

RT = Retention time

DP = Declustering potential

EP = Entrance Potential

CE = Collision Energy

CXP = Collision cell exit potential

S1 Table B. Mycotoxin specific MS/MS settings and standard addition (spike) levels for negative ionization mode

**Mycotoxin**	**Q1 (*m/z*)**	**Q3 (*m/z*)**	**RT (min)**	**DP (V)**	**EP (V)**	**CE (V)**	**CXP (V)**	**Spike (µg/L)**	**Detection range (µg/L)**
ZEN	317.3	175.0	9.5	-125	-10	-32	-15	3	6 - 0.3
		130.9		-125	-10	-38	-15		
α ZEL	319.2	160.0	9.5	-110	-10	-44	-13	20	40 - 2
		130.0		-110	-10	-50	-20		
β ZEL	319.3	160.0	9.1	-110	-10	-44	-13	20	40 - 2
		130.0		-110	-10	-50	-20		
Z14S	397.2	316.9	7.8	-55	-10	-30	-21	5	10 - 0.5
		175.0		-55	-10	-46	-11		
NIV	371.1	281.0	5.0	-55	-10	-20	-19	50	100 - 5
		311.0		-55	-10	-14	-21		
D3G	517.2	457.1	5.4	-45	-10	-18	-13	25	50 - 2.5
		247.0		-45	-10	-32	-17		

Q1 = Precursor ion

Q3 = Product ions

RT = Retention time

DP = Declustering potential

EP = Entrance Potential

CE = Collision Energy

CXP = Collision cell exit potential
