# Supplementary material for: Mycotoxin profiling of 1000 beer samples with a special focus on craft beer
Source: PLoS One. 2017 Oct 5;12(10):e0185887. doi: 10.1371/journal.pone.0185887 (PMC5628871; doi:10.1371/journal.pone.0185887)
Supplement: S2 Table — (PDF) [file pone.0185887.s006.pdf]

S2 Table Intra- and interday precision of the 6-plex assay (%RSD) for fortified samples (in dark ale[a]

**Precision**	**n**	**AFB₁** (0.5 µg/L)	**DON** (100 µg/L)	**FB₁** (100 µg/L)	**OTA** (2 µg/L)	**T-2** (50 µg/L)	**ZEN** (50 µg/L)
Intraday 1	11	7.5	4.3	6.7	3.7	10.9	5.9
Intraday 2	6	3.6	8.1	8.3	10.6	16.7	5.8
Interday	17	6.5	6.5	7.7	7.4	18.4	8.5

[a] n = the total number of fortified dark ale samples
