# Supplementary material for: Mycotoxin profiling of 1000 beer samples with a special focus on craft beer
Source: PLoS One. 2017 Oct 5;12(10):e0185887. doi: 10.1371/journal.pone.0185887 (PMC5628871; doi:10.1371/journal.pone.0185887)
Supplement: S3 Table — (PDF) [file pone.0185887.s007.pdf]

S3 Table Intra- and interday (intraday 1+2) IC50s based on multi-mycotoxin dose-response curves in dark ale (n=2) [a]

	**Experiment**	**Mycotoxin concentration (µg/L)**					
		**AFB_1**	**DON**	**FB_1**	**OTA**	**T-2**	**ZEN**
Intraday 1	1	0.7	31	23	0.3	4.5	2.3
	2	0.7	31	17	0.3	4.9	2.2
	3	0.6	29	11	0.2	4.7	2.2
Intraday 2	1	0.6	24	10	0.3	3.6	1.6
	2	0.7	28	9	0.3	4.1	1.6
	3	0.6	20	8	0.3	3.3	1.5
Median	-	0.7	28	10	0.3	4.3	1.9

[a] n = the total number of replicates for each dose-response curve
