# Supplementary material for: Mycotoxin profiling of 1000 beer samples with a special focus on craft beer
Source: PLoS One. 2017 Oct 5;12(10):e0185887. doi: 10.1371/journal.pone.0185887 (PMC5628871; doi:10.1371/journal.pone.0185887)
Supplement: S4 Table — (PDF) [file pone.0185887.s008.pdf]

S4 Table Intra- and interday determinations of mycotoxin concentrations in a multi-fortified dark ale using the 6-plex assay[a]

**Mycotoxin**	**Mycotoxin concentration (µg/L)**						
	**Spike**	**Intraday 1 (n=11)**		**Intraday 2 (n=6)**		**Interday (n=17)**	
		**Average**	**Median**	**Average**	**Median**	**Average**	**Median**
AFB_1	0.5	0.7	0.6	0.7	0.7	0.7	0.6
DON	100	126	122	116	125	122	124
FB_1	100	286	275	385	414	321	282
OTA	2	3.3	3.3	2.9	2.9	3.2	3.2
T-2	50	48	48	54	54	50	48
ZEN	50	61	58	69	69	64	64

[a] n = the total number of fortified dark ale samples
