# Supplementary material for: Mycotoxin profiling of 1000 beer samples with a special focus on craft beer
Source: PLoS One. 2017 Oct 5;12(10):e0185887. doi: 10.1371/journal.pone.0185887 (PMC5628871; doi:10.1371/journal.pone.0185887)
Supplement: S5 Table — 6-plex immunoassay screening data for A) African traditional beers B) bock beers C) dark ale beers D) dark lager beers E) double India pale ale beers F) eisbock beers G) fruit/vegetable/spice beers H) imperial stout beers I) India pale ale beers J) non/low alcohol beers K) pale ale beers L) pale lager beers M) saison beers N) smoked beers O) sour ale beers P) stout beers Q) strong dark ale beers R) strong pale ale beers S) strong pale lager T) wheat beers. (PDF) [file pone.0185887.s009.pdf]

S5 Table. A 6-plex immunoassay screening data African traditional beers

Sample Number	Designated Style	Craft	Country	%ABV	Mycotoxins (µg/L)					
					AFB_1	DON	ZEN	T-2	OTA	FB_1
272	Maize homebrew	Yes	South Africa	nd	-	-	1.2	9	0.3	27
273	Maize homebrew	Yes	South Africa	nd	-	-	1.2	-	-	-
274	Maize homebrew	Yes	South Africa	nd	-	41	-	-	0.2	18
278	Traditional Ale	No	Zimbabwe	6.0	-	-	-	-	-	376
362	Mqombothi	Yes	South Africa	9.4	-	12	-	-	0.2	9
407	Mqombothi	Yes	South Africa	4.0	-	-	-	-	-	30
409	Sorghum	Yes	South Africa	nd	-	1	-	-	-	17
410	Sorghum	Yes	South Africa	nd	-	-	-	-	-	17
411	Sorghum	Yes	South Africa	nd	-	58	-	-	-	8
412	Sorghum	Yes	South Africa	nd	-	30	-	-	-	12
413	Sorghum	Yes	South Africa	nd	-	31	-	-	-	17
414	Sorghum	Yes	South Africa	nd	-	95	-	-	-	12
415	Sorghum	Yes	South Africa	nd	-	7	-	-	-	17
416	Sorghum	Yes	South Africa	nd	-	-	-	-	-	5
417	Mqombothi	Yes	South Africa	nd	-	107	-	-	-	11
418	Mqombothi	Yes	South Africa	nd	-	112	-	-	-	8
419	Mqombothi	Yes	South Africa	nd	-	-	-	-	-	12
420	Mqombothi	Yes	South Africa	nd	-	107	-	-	-	10
421	Mqombothi	Yes	South Africa	nd	-	-	-	-	-	24
422	Mqombothi	Yes	South Africa	nd	-	12	-	-	-	9
423	Mqombothi	Yes	South Africa	nd	-	10	-	-	-	81
424	Mqombothi	Yes	South Africa	nd	-	-	-	-	-	9
425	Mqombothi	Yes	South Africa	nd	-	100	-	-	-	8
426	Mqombothi	Yes	South Africa	nd	-	56	-	-	-	16
427	Mqombothi	Yes	South Africa	nd	-	91	-	-	-	15
428	Mqombothi	Yes	South Africa	nd	-	43	-	-	-	15
429	Mqombothi	Yes	South Africa	nd	-	-	-	-	-	25
430	Mqombothi	Yes	South Africa	nd	-	57	-	-	-	16
451	Traditional Ale	Yes	South Africa	nd	-	-	-	1	-	28
452	Traditional Ale	Yes	South Africa	nd	-	-	-	-	-	33
453	Traditional Ale	Yes	South Africa	nd	-	-	-	-	-	95

- = no mycotoxins detected

S5 Table. B 6-plex immunoassay screening data bock beers

Sample number	Designated Style	Craft	Country	%ABV	Mycotoxins (µg/L)					
					AFB$_1$	DON	ZEN	T-2	OTA	FB$_1$
29	Bock	Yes	Netherlands	7.0	-	50	-	-	0.3	4
49	Heller Bock	No	Netherlands	8.5	-	5	-	-	0.3	-
169	DoppelBock	No	Germany	7.8	-	68	-	-	0.3	-
210	Bock	No	Netherlands	4.5	-		-	-	0.1	1
220	DoppelBock	No	Germany	7.1	-	56	-	-	0.3	4
224	Bock	Yes	Netherlands	6.5	-		-	-	0.4	-
238	DoppelBock	No	Poland	10.0	-	97	-	-	-	-
249	Dunkler Bock	Yes	United States	5.5	-	29	0.9	-	0.3	-
316	Dunkler Bock	Yes	Denmark	6.5	-	41	-	-	0.3	10
325	DoppelBock	Yes	Norway	8.5	0.3	53	-	13	0.8	37
436	Dunkler Bock	Yes	Netherlands	6.5	-	14	-	10	-	53
456	Heller Bock	No	Germany	7.0	-	23	1.0	5	0.1	2
494	Heller Bock	No	Italy	7.1	-	28	-	-	0.2	6
502	DoppelBock	No	Germany	7.4	0.2	28	-	-	0.2	1
506	Dunkler Bock	No	Netherlands	6.5	0.4	21	-	1	0.2	1
510	Dunkler Bock	No	Netherlands	6.5	-	10	-		0.2	
512	Dunkler Bock	Yes	Netherlands	6.5	0.7	24	-	1	0.2	1
513	Dunkler Bock	Yes	Netherlands	6.2	0.2	-	-	-	0.2	-
514	Dunkler Bock	Yes	Belgium	6.5	-	1	-	-	0.1	-
515	Dunkler Bock	No	Netherlands	7.0	-	13	-	-	-	-
516	DoppelBock	No	Netherlands	7.5	-	9	-	-	-	-
518	DoppelBock	No	Netherlands	7.5	-	-	-	-	0.1	-
519	Dunkler Bock	No	Germany	7.2	-	-	-	1	0.1	-
520	Dunkler Bock	No	Netherlands	7.0	-	27	-	-	0.2	-
521	Dunkler Bock	Yes	Netherlands	6.5	0.8	24	-	-	0.1	1
523	Dunkler Bock	Yes	Netherlands	6.5	1.2	34	-	-		148
532	Helles Bock	Yes	Netherlands	6.0	-	-	0.8	8	0.2	21
535	Bock	Yes	Netherlands	6.0		21	0.9	6	0.3	35
570	Dunkler Bock	Yes	Netherlands	7.5	1.3	11	-	3	0.1	30
659	Dunkler Bock	Yes	Netherlands	7.5	-	-	-	4	0.3	1
711	Bock	No	Belgium	6.5	-	-	-	-	0.2	-
846	Heller Bock	No	Germany	5.8	-	-	0.2	-	0.6	1
859	Dunkler Bock	Yes	USA	5.5	-	-	-	-	-	-
868	Dunkler Bock	No	Germany	5.8	-	-	-	-	0.1	-
870	Specialty Grain	Yes	Netherlands	7.0	-	-	-	-	0.1	-
901	DoppelBock	Yes	Nederland	11.5	-	38	-	-	0.4	-
902	Dunkler Bock	Yes	Nederland	6.5	0.2	-	-	-	0.3	-
921	Heller Bock	No	Germany	8.0	1.1	-	0.2	-	-	-

- = no mycotoxins detected

S5 Table. C 6-plex immunoassay screening data dark ale beers

Sample number	Designated Style	Craft	Country	%ABV	Mycotoxins (µg/L)					
					AFB_1	DON	ZEN	T-2	OTA	FB_1
2	Belgian Strong Dark Ale	No	Belgium	8.5	-	36	-	-		6
38	Abbey Dubbel	Yes	Belgium	7.0	-		-	-	0.3	-
53	Double	Yes	Netherlands	6.5	-	20	-	-		4
116	Belgian Ale	Yes	Belgium	7.0	-	46	-	-	0.5	10
167	Belgian Strong Ale	No	Belgium	8.0	-		4.1	-	0.3	-
200	Old Ale	Yes	England	6.0	-	134	0.7	-	0.4	-
216	Abbey Dubbel	Yes	Poland	7.0	-	40	-	-	0.3	3
314	Brown Ale	Yes	United States	5.5	-	99	-	2	0.3	15
321	Old Ale	Yes	England	7.3	-	44	-	-	0.3	10
338	Brown Ale	Yes	Japan	6.2	-	63	-	-	0.3	13
341	Old Ale	Yes	Scotland	8.0	-	121	-	-		22
361	Mild Ale	Yes	Norway	4.5	-	-	-	3	0.4	14
381	Brown Ale	Yes	Norway	4.5	-	67	-	3	0.7	17
434	Abbey Dubbel	No	Belgium	8.0	-	-	-	3	-	-
439	Brown Ale	No	England	4.7	-	-	-	11	-	23
461	Belgian Strong Ale	No	Belgium	8.5	-	25	1.1	3	-	26
464	Abbey Dubbel	No	Belgium	6.7	-	-	0.2	4	-	4
467	Abbey Dubbel	Yes	Netherlands	6.5	-	10	2.6	5	-	17
507	Belgian Strong Ale	Yes	Belgium	7.5	-	-	-	-	-	-
524	Belgian Strong Ale	Yes	Netherlands	7.0	-	7	-	4	0.2	45
544	Abbey dubbel	No	Belgium	6.3	-	15	-	8	0.3	42
545	Brown Ale	No	Netherlands	11.0	-	-	-	6	0.1	46
557	Abbey dubbel	No	Belgium	8.0	0.3	-	-	2	0.2	4
572	Abbey dubbel	Yes	Netherlands	7.0	1.3	10	-	2	0.2	32
697	Abbey Dubbel	No	Belgium	7.0	-	-	-	2	0.2	-
738	Dunckler	No	Czech republic	7.5	-	20	-	4	0.2	7
739	Brown Ale	Yes	Japan	6.2	-	-	-	8	0.3	1
741	Brown Ale	Yes	USA	6.8	-	-	-	1	0.2	3
750	Brown Ale	Yes	Denmark	7.0	-	1	-	3	0.4	4
779	Brown Ale	Yes	Denmark	5.8	-	5	-		0.3	5
790	Specialty Grain	Yes	Netherlands	7.5	-	58	-	-	0.7	4
838	Abey dubbel	No	Belgium	6.5	-	-	0.1	1	0.5	4
839	Abey dubbel	No	Netherlands	7.5	-	-	-	-	0.5	-
899	Abbey Dubbel	Yes	Nederland	6.5	0.1	-	-		0.3	-
918	Belgian Strong Dark Ale	Yes	Belgium	8.0	1.1	-	-	2	-	1
924	Dunkel Lager	No	Poland	7.5	-	-	-	-	-	9
961	Dark Ale	Yes	Belgium	8.2	-	1	-	-	-	-

- = no mycotoxins detected

S5 Table. D 6-plex immunoassay screening data dark lager beers

Sample number	Designated Style	Craft	Country	%ABV	Mycotoxins (µg/L)					
					**AFB$_1$**	**DON**	**ZEN**	**T-2**	**OTA**	**FB$_1$**
33	Schwarzbier	No	Germany	5.0	-	41	-	-	0.4	9
99	Schwarzbier	No	Germany	4.8	-	18	-	-	0.5	20
123	Dark beer	No	Czech Republic	3.6	-	93	0.2	-	0.5	18
124	Dark beer	No	Czech Republic	3.8	0.8	106	-	4	0.3	10
128	Dark Lager	No	Czech Republic	4.4	-	8	-	3	0.4	7
129	Dark Lager	No	Czech Republic	4.5	-	81	0.2	3	0.3	9
130	Dark Lager	No	Czech Republic	4.5	-	9	-	3	0.4	16
131	Semi-Dark Lager	No	Czech Republic	4.6	-	17	-	4	0.2	15
132	Dark Lager	No	Czech Republic	4.7	-	17	0.1	6	0.4	37
134	Dark Lager	No	Czech Republic	4.8	-	119	-	4	0.4	26
135	Dark Lager	No	Czech Republic	5.2	-	57	-	2	0.4	16
136	Semi-Dark Lager	No	Czech Republic	5.3	-	57	-	1	0.4	5
137	Dark beer	No	Czech Republic	6.3	-	11	-	1	0.3	6
154	Dunkel/ Vienna	No	Slovakia	4.5	-	67	-	-	0.3	15
161	Dark Lager	No	Greece	5.5	-	20	-	-	0.3	5
193	Schwarzbier	No	Czech Republic	3.8	-	33	-	-	0.4	3
196	Schwarzbier	No	Czech Republic	3.7	-	-	0.2	-	0.3	3
387	Dunkel Lager	No	Mexico	6.0	-	-	-	-	0.3	14
391	Dunkel	No	Spain	6.2	-	-	-	1	0.3	24
396	Schwarzbier	No	Spain	4.8	-	23	-	1	0.3	28
401	Dunkel	No	Spain	5.5	-	45	0.6	-	0.4	33
554	Dunkel Lager	Yes	Netherlands	5.0	-	11	-	3	0.3	18
623	Dunkel	No	Ukraine	4.1	-	3	-	5	0.1	3
803	Schwarzbier	No	Netherlands	5.2	3.7	-	-	8	-	20
807	Schwarzbier	No	Czech Republic	3.8	-	-	-	-	-	-
880	Dunkel Lager	No	Poland	4.1	-	48	0.2	3	0.5	4
890	Dunckel Lager	No	Spain	5.4	-	-	-	-	0.2	3
1026	Dunkel	No	Spain	5.5	-	23	-	-	0.1	19

- = no mycotoxins detected

S5 Table E. 6-plex immunoassay screening data double India pale ale beers

Sample Number	Designated Style	Craft	Country	%ABV	Mycotoxins (µg/L)					
					AFB_1	DON	ZEN	T-2	OTA	FB_1
212	American Double / Imperial India Pale Ale	Yes	United Sates	8.0	-	-	-	-	0.3	-
315	Imperial/Double India Pale Ale	Yes	Denmark	13.0	-	55	0.1	-	0.2	13
330	Imperial India Pale Ale	Yes	Norway	10.0	0.1	78	-	-	0.7	30
335	Imperial/Double India Pale Ale	Yes	United States	10.0	-	52	-	-	0.3	4
348	Imperial India Pale Ale	Yes	Belgium	9.0	-	54	-	-	-	3
362	Imperial IPA	Yes	Denmark	9.4	-	12	-	-	0.2	9
366	Black India Pale Ale	Yes	Netherlands	8.0	-	97	-	3	0.2	11
527	India Pale Ale	Yes	Netherlands	9.2	1.0	20	1.4	-	0.1	18
580	Imperial/ Double IPA	Yes	USA	8.0	0.6	9	-	-	0.2	9
592	Imperial India Pale Ale	Yes	USA	9.5	0.3	23	-	-	0.2	18
617	Imperial/ Double IPA	Yes	Belgium	9.0	-	8	-	1	0.3	2
622	India Pale Ale	Yes	Scotland	8.5	-	-	-	-	0.3	-
658	India Pale Ale	Yes	USA	8.3	0.6	5	-	-	0.3	-
672	Imperial India Pale Ale	Yes	USA	9.0	-	2	-	-	0.2	4
688	Imperial India Pale Ale	Yes	Scotland	7.5	-	-	-	-	0.2	1
715	Imperial India Pale Ale	Yes	France	8.0	-	3	-	3	0.3	1
726	Imperial India Pale Ale	Yes	USA	8.2	-	-	1.6	4	0.3	1
754	Imperial India Pale Ale	Yes	USA	8.5	-	-	-	1	0.1	1
863	India Pale Ale	Yes	Germany	8.0	-	-	-	-	0.1	-
877	Imperial India Pale Ale	Yes	USA	8.7	-	-	-	-	-	-
885	Black IPA	Yes	Netherlands	8.0	-	12	-	-	0.5	1
904	Imperial India Pale Ale	Yes	Belgium	8.0	2.3	-	0.8	-	-	8
907	Imperial India Pale Ale	Yes	Germany	8.6	0.8	-	-	-	-	5
909	Imperial India Pale Ale	Yes	Nederland	7.9	3	-	0.8	-	-	4
951	Double IPA	Yes	Nederland	8.2	-	-	-	-	-	-
957	Imperial India Pale Ale	Yes	USA	8.0	-	-	-	-	-	-
967	Imperial India Pale Ale	Yes	Switzerland	9.0	-	-	-	-	0.2	-
979	Imperial India Pale Ale	Yes	USA	9.2	-	-	-	2	-	-
998	Imperial India Pale Ale	Yes	Italy	9.5	-	-	-	-	-	-
999	Imperial India Pale Ale	Yes	France	8.0	-	-	-	-	-	-

- = no mycotoxins detected

S5 Table. F 6-plex immunoassay screening data eisbock beers

Number	Designated Style	Craft	Country	%ABV	Mycotoxins (µg/L)					
					AFB_1	DON	ZEN	T-2	OTA	FB_1
351	Imperial Stout	Yes	Belgium	39.0	-	41	-	-	-	-
517	EisBock	Yes	Netherlands	9.3	1.0	14	-	-	0.2	-
599	Imperial Stout	Yes	Belgium	26.0	2.9	308	-	-	0.2	60
834	Weizen Bock	No	Germany	12.0	-	27	-	-	0.5	7
1022	EisBock	Yes	Netherlands	25.0	-	14	-	-	-	-
1044	IPA EisBock	Yes	Scotland	35.0	-	1	-	-	-	-

- = no mycotoxins detected

S5 Table. G 6-plex immunoassay screening data fruit/vegetable/spice beers

Sample number	Designated Style	Craft	Country	%ABV	Mycotoxins (µg/L)					
					AFB_1	DON	ZEN	T-2	OTA	FB_1
13	Fruit / Vegetable Beer	No	Netherlands	4.8	-	2	-	-	-	5
34	Herbed / Spiced Beer	Yes	Netherlands	8.5	-	28	-	-	0.2	-
56	Herbed / Spiced Beer	Yes	Belgium	7.5	-	-	-	-	0.2	-
57	Fruit / Vegetable Beer	No	Germany	2.5	-	99	-	-	-	15
60	Fruit / Vegetable Beer	No	Netherlands	4.0	-	-	-	-	-	8
95	Fruit / Vegetable Beer	Yes	Netherlands	7.0	-	19	-	-	0.4	17
155	Fruit / Vegetable Beer	Yes	England	5.1	-	46	-	-	-	7
187	Fruit / Vegetable Beer	Yes	Poland	5.5	-	70	0.3	-	0.4	3
195	Fruit / Vegetable Beer	No	Germany	2.5	-	47	-	-	0.4	2
225	Fruit / Vegetable Beer	No	Belgium	5.0	-	-	0.4	-	2.3	4
242	Fruit / Vegetable Beer	No	Germany	2.5	-	127	-	-	-	-
323	American Pale Ale	Yes	Norway	5.5	-	63	-	-	0.3	9
403	Fruit Ale	No	South Africa	10.0	-	-	-	-	-	2
405	Fruit Ale	Yes	South Africa	nd	-	-	-	-	-	-
406	Fruit Ale	Yes	South Africa	nd	-	-	-	-	-	-
408	Fruit Ale	Yes	South Africa	nd	-	-	-	-	-	1
477	Fruit / Vegetable Beer	No	Netherlands	4.0	-	-	-	1	-	14
503	Spice/Herb/Vegetable	Yes	USA	8.0	-	3	-	1	0.2	
552	Fruit Ale	No	Belgium	4.3	0.2	7	-	5	-	17
582	Herbed / Spiced Beer	Yes	USA	15.9	0.9	51	-	-	-	5
613	Fruit / Vegetable Beer	Yes	USA	8.6	0.1	47	-	1	0.3	2
630	Spice/Herb/Vegetable	No	Poland	6.1	-	12	-	1	0.4	3
710	Herbed / Spiced Beer	Yes	Netherlands	7.5	-	-	-	3	0.2	
794	Specialty Grain/herbed	Yes	Netherlands	5.6	-	63	-	-	0.3	5
799	Spice/Herb/Vegetable	Yes	Netherlands	7.0	2.7	-	-	-	-	1
814	Traditional Ale	Yes	Scotland	5.0	0.1	-	-	1	-	2
832	Spice/Herb/Vegetable	Yes	Netherlands	9.0	-	4	-	1	0.5	1
837	Traditional Ale	Yes	Scotland	5.0	-	-	0.1	-	0.3	-
856	Belgian Strong Ale	Yes	Belgium	8.0	-	-	-	-	0.1	-
866	Spice/Herb/Vegetable	No	Germany	4.5	-	-	-	-		-
871	Spice/Herb/Vegetable	Yes	Belgium	7.0	-	-	-	-	0.1	-
963	spiced Belgian Ale	Yes	Belgium	5.5	-	-	-	-	0.2	-
982	Spice/Herb/Vegetable	Yes	Spain	4.5	-	-	-	-	0.2	-
986	Mead	Yes	Netherlands	9.0	-	-	-	-	-	-
987	Fruit / Vegetable Beer	Yes	Denmark	14.0	-	-	-	-	-	-
989	Spice/Herb/Vegetable	Yes	Sweden	5.9	-	2	-	-	-	-
997	Spice/Herb/Vegetable	Yes	Netherlands	4.2	-	-	-	-	0.2	-

- = no mycotoxins detected

S5 Table. H 6-plex immunoassay screening data imperial stout beers

Sample Number	Designated Style	Craft	Country	%ABV	Mycotoxins (µg/L)					
					AFB_1	DON	ZEN	T-2	OTA	FB_1
10	Russian Imperial Stout	Yes	Netherlands	10.2	-	144	-	-	0.4	16
11	Russian Imperial Stout	Yes	Norway	15.5	-	140	-	-	0.3	16
20	Russian Imperial Stout	Yes	Belgium	13.0	-	135	-	-	0.3	15
32	Russian Imperial Stout	Yes	Netherlands	11.0	-	114	-	-	0.2	12
87	Russian Imperial Stout	Yes	United States	11.0	-	150	-	-	0.5	50
88	Russian Imperial Stout	Yes	United States	12.0	-	131	-	-	0.6	35
117	Russian Imperial Stout	Yes	Belgium	10.0	-	90	-	-	0.4	8
153	Russian Imperial Stout	Yes	United States	9.5	-	140	-	-	0.4	42
173	Imperial Stout	Yes	Belgium	13.0	-	118	-	-	0.4	17
181	Imperial Stout	Yes	Netherlands	10.0	-	76	-	-	0.3	17
183	Russian Imperial Stout	Yes	United States	15.0	2.2	475	-	2	0.3	44
201	Russian Imperial Stout	Yes	United States	11.0	-	52	0.3	-	0.4	10
211	Imperial Stout	Yes	United States	9.5	-	149	-	-	0.4	14
247	American Porter	Yes	United States	8.2	-	29	0.4	-	0.3	3
252	Russian Imperial Stout	Yes	United States	13.0	-	114	-	-	0.2	4
259	Russian Imperial Stout	Yes	Norway	11.0	-	22	-	-	-	2
292	Russian Imperial Stout	Yes	Denmark	10.4	-	21	-	-	0.3	29
306	Imperial Stout	Yes	USA	11.0	-	142	0.1	-	0.2	31
307	Imperial Stout	Yes	Scotland	17.2	-	136	-	-	-	36
317	Imperial Stout	Yes	Norway	9.0	-	62	0.3	5	0.3	8
318	Foreign Stout	Yes	Belgium	9.0	-	86	-	-	0.4	19
320	Imperial Stout	Yes	Netherlands	11.4	-	111	-	-	0.2	16
329	Imperial Stout	Yes	England	10.3	-	167	-	-	0.2	30
332	Imperial Stout	Yes	Netherlands	13.0	-	140	-	-	0.2	26
344	Imperial/Strong Porter	Yes	Netherlands	11.0	-	78	-	-	-	12
346	Imperial/Strong Porter	Yes	Denmark	8.3	-	22	-	1	0.3	8
347	Imperial/Strong Porter	Yes	England	10.5	-	97	-	-	-	20
352	Imperial Stout	Yes	England	9.5	-	91	-	-	0.3	20
354	Imperial Stout	Yes	Belgium	13.0	-	95	-	-	0.2	19
356	Imperial Stout	Yes	Netherlands	11.0	1.5	45	-	57	0.2	33
365	Imperial Stout	Yes	Netherlands	10.0	-	58	-	-	0.2	12
367	Imperial Stout	Yes	Denmark	10.5	-	136	-	-	0.1	20
370	Imperial Porter	Yes	Netherlands	11.0	-	120	-	-	-	12
376	Imperial Stout	Yes	United States	10.8	-	122	-	2	0.4	16
389	Imperial Stout	Yes	Netherlands	10.0	-	85	-	-	0.3	33
438	Imperial/Strong Porter	Yes	Netherlands	11.0	0.4	82	-	3	-	108
446	Imperial Stout	Yes	United States	8.2	0.8	46	-	8	-	123
468	Imperial Stout	Yes	Canada	9.5	1.2	114	-	6	0.4	63
501	Imperial Stout	Yes	USA	8.8	1.9	95	0.1	-	0.3	5
505	Imperial Stout	Yes	Netherlands	10.7	1.2	47	-	-	0.3	1
569	Imperial Stout	Yes	USA	12.5	1.1	65	-	-	0.3	31

Sample number	Designated Style	Craft	Country	%ABV	Mycotoxins (µg/L)					
					AFB_1	DON	ZEN	T-2	OTA	FB_1
576	Imperial Stout	Yes	USA	9.6	2.0	39	0.8	-	0.1	53
581	Imperial Stout	Yes	USA	9.6	1.6	167	-	1	0.1	54
585	Imperial Stout	Yes	USA	13.0	1.1	89	-	-	0.1	64
587	Imperial Stout	Yes	USA	19.5	1.2	36	-	-	-	50
588	Imperial Stout	Yes	USA	13.0	2.4	158	-	2	0.4	76
589	Imperial Stout	Yes	USA	9.6	1.7	85	-	1	0.2	63
593	Imperial Stout	Yes	USA	9.6	3.1	175	-	1	0.2	48
594	Imperial Stout	Yes	USA	13.0	2.7	134	-	2	0.3	56
596	Imperial Stout	Yes	USA	13.0	2.8	280	-	-	0.2	62
607	Imperial Stout	Yes	Denmark	10.9	-	152	-	-	-	39
612	Imperial Stout	Yes	USA	9.0	1.3	89	-	1	0.3	12
624	Imperial Stout	Yes	Netherlands	10.7	0.1	20	-	-	-	3
625	Imperial Stout	Yes	Netherlands	10.0	1.1	21	-	-	1.0	4
626	Imperial Stout	Yes	Belgium	11.0	0.4	54	-	-	0.1	8
631	Imperial Stout	Yes	Norway	14.0	-	125	-	-	-	10
638	Imperial Stout	Yes	USA	10.6	1.4	58	0.4	7	0.2	24
644	Imperial Stout	Yes	Netherlands	11.0	0.6	21	-	-	0.1	9
654	Imperial Stout	Yes	USA	10.2	1.0	6	-	4	0.2	14
664	Imperial Stout	Yes	Scotland	12.0	-	17	-	-	-	2
666	Imperial Stout	Yes	Netherlands	12.5	-	15	-	1	0.3	2
669	Baltic Porter	No	Poland	9.0	0.5	44	-	1	0.2	8
674	Imperial Stout	Yes	USA	10.5	0.4	56	-	-	-	9
676	Baltic Porter	Yes	USA	9.0	-	20	-	-	0.1	6
678	Imperial Stout	Yes	Denmark	13.0	-	34	-	-	0.2	9
687	Imperial Stout	Yes	Denmark	10.1	-	34	-	-	0.1	9
692	Imperial Stout	Yes	Belgium	12.0	-	18	-	-	-	5
717	Russian Imperial Stout	Yes	USA	11.0	0.3	99	-	-	0.1	11
742	Imperial Stout	Yes	Netherlands	10.0	0.6	17	-	-	0.1	9
745	Imperial Stout	Yes	Canada	9.5	2.0	56	-	3	0.4	34
746	Imperial Stout	Yes	Denmark	10.3	0.7	64	-	1	0.4	27
748	Imperial Stout	Yes	USA	11.0	1.5	101	-		0.1	32
751	Imperial Stout	Yes	Denmark	10.0	-	79	-	2	0.3	23
755	Imperial Stout	Yes	Denmark	10.4	-	2	-	2	0.3	11
761	Stout	Yes	Denmark	10.1	-	33	-	4	0.4	18
764	Imperial Stout	Yes	USA	11.0	-	21	-	-	0.2	8
765	Imperial Stout	Yes	USA	8.3	2.1	-	-	-	-	3
766	Foreign Stout	Yes	USA	8.8	2.3	14	-	-	0.3	10
767	Imperial Stout	Yes	USA	8.0		2	-	-	0.3	7
770	Russian Imperial Stout	Yes	Denmark	10.3	2.2	31	-	-	-	8
771	Baltic Porter	No	Poland	8.0	-	21	-	-	0.3	4
778	Imperial Stout	Yes	Denmark	12.0	1.4	82	-	-	0.2	21
780	Imperial Stout	Yes	Canada	8.5	0.3	54	-	-	0.3	13

Sample number	Designated Style	Craft	Country	%ABV	Mycotoxins (µg/L)					
					AFB_1	DON	ZEN	T-2	OTA	FB_1
781	Imperial Stout	Yes	Netherlands	11.0	-	32	-	-		6
795	Stout	Yes	Netherlands	9.0	-	98	-	-	0.7	8
816	Russian Imperial Stout	Yes	Denmark	10.0	-	-	-	-	-	-
818	Russian Imperial Stout	Yes	USA	15.0	-	-	-	-	0.3	4
820	spiced Imperial Stout	Yes	USA	11.0	-	-	-	-	0.2	11
822	spiced Imperial Stout	Yes	USA	11.0	-	102	-	-		12
823	spiced Imperial Stout	Yes	USA	11.0	-	168	-	-		9
824	spiced Imperial Stout	Yes	USA	11.0	-	129	-	-	0.3	20
825	Russian Imperial Stout	Yes	England	15.0	-	13	-	2	0.3	9
830	Imperial Stout	Yes	USA	11.1	-	-	-	1	0.5	14
843	Imperial Stout	Yes	Netherlands	9.0	-	33	0.1	-	0.5	4
848	Imperial/ Strong Porter	Yes	USA	11.0	-	16	-	-	0.5	1
853	Americal Double	Yes	USA	9.5	-	32	-	-	0.3	2
854	Imperial Stout	Yes	Netherlands	14.0	-	8	-	-	0.4	1
865	Imperial Stout	Yes	USA	9.5	-	29	-	-	0.1	-
867	Imperial Strong Porter	Yes	USA	11.0	-	74	-	-	-	1
876	Imperial Stout	Yes	USA	19.5	-	-	-	-	0.1	-
882	Baltic Porter	No	Poland	9.0	-	42	-	-	0.4	22
889	Imperial Stout	Yes	Netherlands	9.8	-	25	-	-	-	-
892	Imperial Stout	Yes	Netherlands	11.0	0.5	62	-	-	0.4	5
893	Stout	Yes	Netherlands	9.5		41	-	-	0.3	3
897	Imperial Porter	Yes	USA	9.2	0.2	90	-	-	0.4	2
900	Imperial Stout	Yes	Nederland	11.0	-	154	-	-	0.3	2
911	Russian Imperial Stout	Yes	USA	9.4	0.6	177	0.9	3	-	22
919	Imperial Stout	Yes	Canada	8.0	2.2	-	-	2	-	16
955	Imperial Stout	Yes	Netherlands	12.6	-	5	-	-	-	-
964	Imperial Stout	Yes	USA	13.9	-	24	-	-	-	-
965	Imperial Stout	Yes	USA	10.5	-	30	-	-	0.3	3
969	Imperial Stout	Yes	Belgium	10.0	-	2	-	-	-	-
971	Imperial Stout	Yes	USA	9.5	-	1	-	-	-	1
978	Imperial Stout	Yes	Denmark	10.0	-	144	-	13	0.3	43
980	Imperial Stout	Yes	Belgium	10.0	-	-	-	-	-	-
988	Imperial Stout	Yes	Denmark	17.5	-	-	-	-	-	-
990	Imperial Stout	Yes	Netherlands	9.8	-	-	-	-	0.2	
992	Imperial Stout	Yes	Denmark	12.1	-	-	-	-	-	-
993	Imperial Stout	Yes	Sweden	10.0	-	-	-	-	0.2	-
996	Stout	Yes	Italy	12.0	-	-	-	-	-	-
1000	Imperial Stout	Yes	USA	12.7	-	-	-	-	0.3	-
1010	Stout	Yes	Netherlands	8.0	-	67	-	-	-	-
1013	Imperial Stout	Yes	England	10.0	-	5	-	-	0.3	-
1034	Imperial Stout	Yes	USA	12.0	-	220	-	-	0.1	16
1039	Imperial Stout	Yes	Netherlands	10.8	-	74	-	-	0.1	-

Sample number	Designated Style	Craft	Country	%ABV	Mycotoxins (µg/L)					
					AFB_1	DON	ZEN	T-2	OTA	FB_1
1047	Imperial Stout	Yes	USA	12.0	-	82	-	-	0.1	-

- = no mycotoxins detected

S5 Table. I 6-plex immunoassay screening data India pale ale beers

Sample Number	Designated Style	Craft	Country	%ABV	Mycotoxins (µg/L)					
					**AFB$_1$**	**DON**	**ZEN**	**T-2**	**OTA**	**FB$_1$**
4	Extra Special / Strong Bitter	No	England	5.4	-	9	-	-	0.3	-
21	India Pale Ale	Yes	Netherlands	6.2	-	32	-	-	0.5	12
62	India Pale Ale	Yes	United States	7.1	0.8	16	-	-	0.3	13
89	India Pale Ale	Yes	United States	7.0	-	4	-	-	0.5	4
112	India Pale Ale	Yes	Netherlands	7.0	-	21	-	-	0.4	4
145	IPA	No	Belgium	7.5	-	7	-	-	0.6	3
148	India Pale Ale	No	England	5.3	-	-	0.4	-	0.4	-
151	India Pale Ale	Yes	Denmark	6.9	-	109	-	-	0.4	24
177	India Pale Ale	Yes	Belgium	7.0	-	146	-	-	0.4	6
192	Bitter	No	England	3.8	-	28	-	-	0.4	-
240	Black IPA	Yes	Netherlands	7.2	-	20	-	-	0.3	2
293	Bitter	Yes	Netherlands	4.5	-	26	0.6	-	0.3	5
294	India Pale Ale	Yes	England	5.0	-	14	1.5	-	0.3	1
300	India Pale Ale	Yes	England	6.0	-	-	-	6	0.3	2
372	Bitter	Yes	Sweden	5.7	-	69		3	0.4	11
375	Bitter	Yes	England	3.9	-	8	0.2	1	0.3	5
385	India Pale Ale	Yes	England	5.9	-	7	-	1	-	4
449	India Pale Ale	Yes	United States	7.5	0.6	5	-	2	-	-
479	India Pale Ale	Yes	Belgium	7.0	0.4	84	-	-	-	-
482	Belgian Strong Ale	Yes	Netherlands	6.7	1.4	11	-	-	-	6
504	Bitter	Yes	USA	5.6	0.4	-	-	-	-	-
534	India Pale Ale	Yes	Netherlands	4.5	0.5	-	0.8	5	0.4	22
565	Bitter	Yes	Netherlands	4.5	0.3	8	-	1	0.3	10
571	Black IPA	Yes	USA	6.9	1.1	42	-	1	0.3	67
618	India Pale Ale	Yes	France	7.0	-	17	0.1	2	0.2	1
683	India Pale Ale	Yes	USA	6.9	-	-	-	-	0.3	1
719	India Pale Ale	Yes	Denmark	6.8	-	-	1.7	6	0.3	1
732	Bitter ESB	Yes	Denmark	4.8	-	-	-	10	0.4	1
762	India Pale Ale	Yes	Japan	7.5	-	-	-	2	0.3	3
772	India Pale Ale	Yes	France	7.0	-	-	-	-	0.3	-
789	India Pale Ale	Yes	Netherlands	6.6	-	69	-	-	0.6	5
796	Bitter	No	England	3.5	-	-	-	1	-	0
800	Bitter	Yes	Netherlands	4.5	2.6	-	-	-	-	7
845	India Pale Ale	Yes	Netherlands	7.0	-	-	0.1	-	0.5	-
850	India Pale Ale	Yes	Netherlands	6.2	-	-	-	-	0.3	-
860	IPA	Yes	Germany	5.1	-	-	-	-	-	-
869	India Pale Ale	Yes	Germany	6.1	-	-	-	-	-	-
883	India Pale Ale	Yes	Netherlands	6.0	-	-	-	-	0.3	-
906	IPA	Yes	Germany	4.9	1.6	-	-	-	0.2	5
922	India Pale Ale	Yes	Nederland	6.8	2.6	-	-	2	-	2
950	India Pale Ale	Yes	Poland	6.2	-	9	-	-	-	-

Sample number	Designated Style	Craft	Country	%ABV	Mycotoxins (µg/L)					
					AFB_1	DON	ZEN	T-2	OTA	FB_1
952	White IPA	Yes	Poland	5.6	-	6	-	-	0.3	-
991	India Pale Ale	Yes	Spain	5.4	-	-	-	-	0.2	-

- = no mycotoxins detected

S5 Table. J 6-plex immunoassay screening data non/low alcohol beers

Sample Number	Designated Style	Craft	Country	%ABV	Mycotoxins (µg/L)					
					AFB_1	DON	ZEN	T-2	OTA	FB_1
47	Non - Alcoholic	No	Netherlands	0.0	-	-	-	-	0.4	
52	Non - Alcoholic	Yes	Germany	0.0	-	11	-	-	0.3	
61	Non - Alcoholic	No	Netherlands	0.0	-	-	0.1	-	0.7	
65	Non - Alcoholic	No	Germany	0.0	-	-	-	-	0.3	
67	Non - Alcoholic	No	Belgium	0.0	-	-	-	-	0.3	1
68	Non - Alcoholic	No	Germany	0.0	-	-	-	-	0.3	1
72	Non - Alcoholic	No	Belgium	0.5	-	-	0.4	-	0.3	
85	Non - Alcoholic	No	Germany	0.4	-	5	0.2	1	0.5	3
121	Non - Alcoholic	No	Czech Republic	0.0	-	36	0.5	4	0.2	3
122	Non - Alcoholic	No	Czech Republic	0.0	-	4	0.4	-	0.5	3
158	Non - Alcoholic	No	Netherlands	0.0	-	-	0.6	2	0.3	-
234	Non - Alcoholic	No	Germany	0.0	-	-	-	-	0.3	-
235	Non - Alcoholic	No	Germany	0.0	-	-	-	-	0.3	-
237	Non - Alcoholic	No	Germany	0.5	-	14	-	-	0.3	-
243	Non - Alcoholic	No	Germany	0.0	-	-	-	-	0.3	-
244	Non - Alcoholic	No	Germany	0.5	-	-	0.2	-	0.2	-
248	Non - Alcoholic	No	Germany	0.3	-	-	0.1	6	0.3	1
253	Non - Alcoholic	No	Germany	0.0	-	-	-	-	0.4	-
255	Non - Alcoholic	No	Germany	0.0	-	-	-	-	0.3	-
257	Non - Alcoholic	No	Germany	0.0	-	-	-	-	0.3	-
258	Non - Alcoholic	No	Germany	0.0	-	-	-	-	0.3	-
283	Low Alcohol	No	Namibia	2.4	-	-	-	3	0.3	-
302	Non - Alcoholic	No	Netherlands	0.0	-	-	-	6	0.3	5
308	Non - Alcoholic	No	Germany	0.1	-	-	-	6	0.3	8
393	Low-Alcoholic	No	Spain	2.4	-	-	-	3	0.3	30
394	Non - Alcoholic	No	Spain	0.0	-	-	-	3	0.3	26
395	Non - Alcoholic	No	Spain	0.0	-	-	-	-	-	28
398	Non - Alcoholic	No	Spain	0.0	-	-	-	-	0.3	34
466	Non - Alcoholic	No	Germany	0.0	-	-	0.4	14	0.2	20
500	Non - Alcoholic	No	Italy	0.1	-	-	-	2	0.1	-
675	Low Alcohol	Yes	Belgium	2.0	-	4	-	2	0.3	6
686	Low Alcohol	Yes	Denmark	0.6	-	33	-	3	0.2	2
931	Low Alcohol	No	Spain	1.0	-	-	-	6	0.2	5
937	Low Alcohol	No	Spain	0.8	-	-	5.7	5	0.2	10
940	Pale Ale	No	Spain	0.0	-	-	-	-	0.3	-
944	Low Alcohol	No	Spain	0.0	-	-	-	-	-	11

- = no mycotoxins detected

S5 Table. K 6-plex immunoassay screening data pale ale beers

Sample Number	Designated Style	Craft	Country	%ABV	Mycotoxins (µg/L)					
					AFB_1	DON	ZEN	T-2	OTA	FB_1
3	Belgian Pale Ale	No	Belgium	4.5	-	-	-	-	0.3	6
8	Belgian Pale Ale	Yes	Netherlands	4.7	-	-	-	1	0.4	8
23	Pilsner	Yes	Netherlands	4.7	-	4	-	-	0.3	-
35	Belgian Ale	Yes	Netherlands	7.5	-	51	-	-	0.2	-
50	Abbey Tripel	Yes	Netherlands	8.0	-	-	-	-	0.1	-
51	Abbey Tripel	Yes	Belgium	8.0	-	7	-	-	0.3	-
54	Abbey Tripel	Yes	Belgium	8.0	1.2	4	-	5	-	-
59	Belgian Ale	Yes	Netherlands	4.5	-	-	-	-	-	-
75	Abbey Tripel	Yes	Netherlands	8.0	-	10	-	-	0.2	-
78	Blond	Yes	Netherlands	6.0	-	-	-	-	0.3	-
79	Blond	No	Belgium	8.0	-	-	-	-	0.3	-
81	Ale	Yes	England	5.0	-	-	-	-	0.1	2
94	Belgian Pale Ale	No	Belgium	5.4	-	12	-	-	0.3	
97	Belgian Ale	Yes	Netherlands	5.5	0.7	92	-	1	0.2	22
114	Belgian Ale	Yes	Netherlands	6.5	-	35	-	-	0.3	8
115	English Pale Ale	No	England	4.7	-	5	-	-	0.5	4
142	Irish red Ale	No	Ireland	5.0	-	-	0.7	2	0.3	5
162	Abbey Tripel	No	Belgium	7.8	-	26	-	-	0.3	-
166	Belgian Ale	No	Belgium	7.0	-	-	-	-	0.3	-
176	Belgian Ale	Yes	France	6.5	1.4	-	-	1	0.3	-
179	Pale Ale	No	Germany	5.6	-	14	-	-	0.5	3
180	Pale Ale	Yes	England	5.0	-	-	-	-	0.4	3
186	Belgian Pale Ale	Yes	Belgium	5.8	-	-	-	-	0.2	1
194	American Strong Ale	Yes	United States	7.2	-	147	-	-	-	2
197	Belgian Ale	Yes	Netherlands	5.5	-	38	0.3	-	0.4	-
217	Pale Ale	No	Poland	5.7	-	26	-	-	-	7
226	Pale Ale	No	France	5.8	-	-	-	-	3.1	-
232	Abbey Tripel	Yes	Netherlands	8.0	-	36	-	-	0.2	-
251	Belgian Pale Ale	Yes	Belgium	6.3	-	-	-	-	-	-
299	Irish Red Ale	No	Ireland	4.3	-	-	1.8	2	0.3	2
304	Pale Ale	No	Finland	5.2	-		-	1	0.2	
312	American Pale Ale	Yes	Norway	4.5	-	30	-	3	0.3	11
345	Belgian Ale	Yes	Belgium	6.2	-	-	-			
349	Belgian Ale	Yes	Denmark	8.7	-	10	-	35	0.4	9
373	Pale Ale	Yes	Sweden	4.9	-	51	-	4	0.4	10
374	Weizen Bock	Yes	Netherlands	6.9	-	6	-	4	0.1	1
378	Blond Ale	Yes	Belgium	8.0	-	8	-			3
379	Pale Ale	Yes	England	5.2	-	56	0.2	1	0.3	13
380	Belgian Ale	Yes	Norway	6.0	-	8	-	7	0.4	2
432	Belgian Strong Ale	No	Belgium	8.5	-	5	-	6	-	19
437	Abbey Tripel	Yes	Belgium	8.0	0.6	-	-	4	0.1	-

Sample number	Designated Style	Craft	Country	%ABV	Mycotoxins (µg/L)					
					AFB_1	DON	ZEN	T-2	OTA	FB_1
440	Abbey Tripel	Yes	Belgium	8.0	-	1	-	5	-	-
443	Belgian Strong Ale	Yes	United States	7.8	-	-	-	5	-	23
457	Belgian Ale	No	Netherlands	6.2	-	-	0.4	5	-	3
490	Belgian Ale	Yes	Belgium	7.6	-	24			0.2	16
511	Belgian Ale	Yes	Belgium	7.0	-	-		-	-	-
530	Blond	No	Netherlands	6.0	-	-	1.4	7	0.3	22
537	Belgian Pale Ale	No	Belgium	6.0	-	-	0.5	7	0.1	14
556	Blond	No	Netherlands	6.2	-	1	-	4	0.1	4
559	Blond	No	Belgium	7.5	0.4	4	0.1	4	0.2	7
564	Blond	No	Belgium	6.6	-	4	-	1	0.2	11
573	Belgian Ale	Yes	Belgium	4.8	0.5	30	-	4	0.2	16
574	English Strong Ale	No	England	6.3	0.9	11	0.7	-	0.2	-
583	Belgian Ale	Yes	Belgium	9.0	1.8	83		1	0.2	93
590	Abbey Tripel	Yes	Belgium	8.0	-	11	-	1		11
598	Blond	Yes	Belgium	5.5	-	8	-	5	0.1	1
604	Pale Ale	No	Belgium	7.0	-	-	-	3	-	-
605	Kölsch	No	Germany	4.8	2.1	22	-	5	0.6	9
616	English Strong Ale	No	England	8.5	-	20	-	-	0.2	2
628	Specialty Grain	No	Belgium	8.2	-	44	-	1	0.1	-
648	Abbey Tripel	Yes	Belgium	8.0	-	12	-	6	-	10
649	American Strong Ale	Yes	USA	7.0	-	4	-	2	-	10
651	Belgian Ale	Yes	Italy	5.5	-	-	-	7	0.1	11
652	Belgian Ale	Yes	Italy	6.5	-	-	-	8	0.2	10
656	Belgium Ale	Yes	Belgium	5.0	0.3	-	-	8	0.1	12
657	Amber Ale	Yes	Netherlands	5.0	-	2	-	5	0.4	4
663	English Strong Ale	No	Scotland	7.2	-	-	-	4	0.3	1
667	Pale Ale	Yes	France	8.0	-	-	-	1	0.0	
671	Belgian Ale	No	Belgium	4.5	0.4	3	-	3	0.4	1
695	Amber Ale	Yes	Netherlands	6.4	-	3	-	5	0.4	2
700	Belgium Ale	No	Belgium	7.0	-	-	-	-	0.2	-
716	Belgium Strong Ale	No	Belgium	8.0	-	-	-	-	0.2	-
720	Belgian Triple	Yes	Denmark	8.5	-	-	0.3	5	0.3	1
733	American Pale Ale	Yes	Denmark	5.0	-	-	0.9	10	0.3	
756	Belgian Ale	Yes	Belgium	6.0	-	-		3	0.2	
786	Amber Ale	No	Netherlands	5.3	-	46	0.3	1	1.1	14
788	Pale Ale	No	Belgium	5.0	-	28	0.7	-	2.0	
791	Strong Pale Ale	No	England	6.5	-	58	0.4	-	1.7	3
793	Pale Ale	Yes	Netherlands	5.6	-	61	0.6	-	0.6	5
797	Traditional Ale	Yes	Netherlands	6.0	-	-	-	3	-	0
798	Belgian Ale	Yes	Netherlands	7.4	2.7	-	-	7	-	5
801	Belgian Ale	Yes	Netherlands	6.0	-	-	-	4	-	13
810	Belgian Ale	Yes	Norway	6.0	-	16	-	-	-	-
811	Belgian Ale	Yes	Netherlands	7.2	-	-	-	-	-	-

Sample number	Designated Style	Craft	Country	%ABV	Mycotoxins (µg/L)					
					AFB_1	DON	ZEN	T-2	OTA	FB_1
862	Rye	Yes	Netherlands	5.3	-	-	-	-	0.1	-
884	Belgian Ale	Yes	Belgium	5.0	0.3	34	0.6	8	0.6	5
894	Belgian Pale Ale	No	Belgium	6.0	-	-	-	-	0.2	-
905	Pale Ale	Yes	Belgium	7.6	2.2	-	-	-	-	1
913	Belgian Ale	Yes	Belgium	6.2	2.2	-	0.6	2	0.1	-
958	Belgian Ale	Yes	Nederland	5.6	-	-	-	-	0.2	-
959	Specialty Grain	Yes	Belgium	8.5	-	-	-	-	-	-
981	Pale Ale	Yes	Scotland	3.8	-	7	-	-	0.2	-
1009	American Pale Ale	Yes	Denmark	6.0	-	6	-	-	0.3	-
1030	English Pale Ale	No	Spain	4.8	-		-	-	0.1	-
1036	Belgian Pale Ale	Yes	Belgium	4.5	-	13	-	-	-	-

- = no mycotoxins detected

S5 Table. L 6-plex immunoassay screening data pale lager beers

Sample Number	Designated Style	Craft	Country	%ABV	Mycotoxins (µg/L)					
					AFB$_1$	DON	ZEN	T-2	OTA	FB$_1$
6	Adjunct Lager	No	Mexico	4.6	-	-	-	-	0.3	-
7	Pilsner	No	Germany	6.5	-	9	-	-	0.2	-
12	Pale Lager	No	Spain	5.2	-	-	-	-	0.4	15
24	Pilsner	No	Germany	4.9	-	4	-	-	0.4	1
31	Helles Lager	No	Germany	4.9	-	4	-	-	0.3	-
39	Adjunct Lager	No	Mexico	4.6	-	-	-	-	0.4	-
40	Pale Lager	No	Netherlands	5.0	-	-	-	-	0.4	-
42	Premium Lager	No	China	4.5	-	-	-	-	0.4	-
43	Pilsner	Yes	Netherlands	5.0	-	-	-	-	0.3	-
44	Pilsner	No	Netherlands	5.0	-	-	-	-	0.2	-
45	Pilsner	No	Turkey	5.0	-	-	-	-	0.4	-
46	Pilsner	No	Netherlands	5.3	-	-	-	-	0.3	-
48	Specialty/Gluten Free	No	Germany	5.0	-	4	-	3	-	20
55	Helles Lager	No	Germany	5.2	-	17	-	1	-	15
64	Pilsner	No	Netherlands	5.0	-	-	-	-	-	-
66	Pilsner	No	Belgium	5.2	-	-	-	-	0.2	-
70	Pilsner	No	Czech Republic	4.4	-	42	-	-	0.3	1
71	Pilsner	No	Germany	5.1	-	-	-	-	0.3	-
73	Pilsner	No	Czech Republic	5.0	-	14	0.1	-	0.2	-
83	Pilsner	No	Netherlands	5.0	-	-	-	1	0.4	-
91	Pale Lager	No	Spain	5.4	-	-	-	-	-	-
93	Pilsner	No	Germany	4.9	-	12	-	-	0.5	4
96	Adjunct Lager	No	United States	5.0	-	-	-	-	0.4	-
103	Pilsner	No	Netherlands	5.0	-	-	-	-	0.4	-
105	Pilsner	No	Netherlands	5.0	-	-	-	-	0.3	-
106	Pilsner	No	Belgium	5.0	-	-	-	-	0.4	-
107	Pilsner	No	Germany	5.0	-	-	-	-	0.3	-
108	Pilsner	No	Netherlands	5.0	-	-	-	-	0.5	7
109	Pilsner	No	Netherlands	5.0	-	-	-	1	0.5	6
110	Pilsner	No	Germany	4.9	-	-	-	-	0.5	5
111	Pilsner	No	Greece	5.0	-	10	-	-	0.4	4
118	Pilsner	No	Netherlands	5.0	-	-	0.6	-	0.5	-
120	Pilsner	No	Netherlands	5.0	-	-	-	-	0.4	-
125	Pale Lager	No	Czech Republic	4.0	-	20	0.4	7	0.3	12
126	Pale Lager	No	Czech Republic	4.0	-	-	0.2	6	0.3	6
127	Pale Lager	No	Czech Republic	5.0	-	-	-	4	0.3	2
133	Pale Lager	No	Czech Republic	4.8	-	18	-	5	0.4	12
139	Pale Lager	No	Czech	5.0	-	24	-	-	0.3	2
163	Pilsner	No	Netherlands	5.0	-	-	-	-	0.4	-
190	Premium Lager	No	Poland	5.5	-	-	-	-	0.3	-

Sample number	Designated Style	Craft	Country	%ABV	Mycotoxins (µg/L)					
					AFB_1	DON	ZEN	T-2	OTA	FB_1
191	Premium Lager	Yes	England	5.0	-	-	-	-	0.4	-
199	Premium Lager	No	Poland	5.6	-	-	-	-	0.5	-
208	Pale Lager	No	Poland	5.0	-	31	-	-	-	9
209	Pilsner	No	Netherlands	5.0	-	-	0.8	-	0.3	2
214	Pilsner	No	Germany	4.8	-	-	-	-	0.3	1
215	Pale Lager	No	Poland	5.2	-	1	0.1	-	0.5	36
218	Premium Lager	No	Poland	5.2	-	-	-	-	0.4	1
221	Pilsner	No	France	5.5	-	-	-	-	0.2	-
222	Pilsner	No	France	4.2	-	-	**0.9**	-	0.3	-
223	Pilsner	No	Netherlands	5.0	-	-	**1.3**	-	0.3	-
239	Pilsner	No	Netherlands	5.0	-	-	-	-	0.3	-
241	Helles Lager	No	Germany	4.7	-	-	-	-	0.3	-
246	Pilsner	No	Germany	4.9	-	-	-	-	0.3	-
250	Zwickel/Keller/Landbier	No	Germany	4.9	-	-	-	-	0.3	-
260	Pale Lager	No	China	4.3	-	-	-	-	0.3	-
261	Pale Lager	No	China	4.0	-	-	-	-	0.3	-
262	Pale Lager	No	China	5.0	-	-	-	-	0.3	-
263	Pale Lager	No	China	5.0	-	-	-	-	0.3	-
264	Adjunct Lager	No	Australia	3.5	-	-	-	-	0.3	-
265	Euro Pale Lager	No	Australia	3.5	-	-	-	-	0.3	-
266	Adjunct Lager	No	Australia	4.6	-	-	-	-	0.3	-
269	Pale Lager	No	Japan	5.5	-	-	-	-	-	-
270	Pale Lager	No	Japan	5.5	-	-	1.6	-	-	-
271	Pale Lager	No	Japan	5.0	-	-	0.1	-	-	-
276	Pale Lager	No	Kenya	4.2	-	-	-	-	0.3	-
277	Pale Lager	No	Kenya	5.0	-	-	-	-	0.2	-
279	Pilsner	No	South Africa	5.0	-	-	-	2	0.3	-
280	Specialty Grain	No	Zimbabwe	5.0	-	-	-	2	0.3	11
281	Pale Lager	No	Namibia	4.0	-	-	-	2	0.2	-
282	Premium Lager	No	Namibia	4.0	-	-	-	-	0.2	-
284	Lager	No	South Africa	5.0	-	-	-	2	0.3	-
285	Lager	No	South Africa	4.0	-	22	-	-	0.3	-
287	Pale Lager	No	South Africa	5.0	-	18	-	-	0.3	-
288	Pale Lager	No	South Africa	5.0	-	6	-	-	0.3	11
289	Pale Lager	No	South Africa	5.5	-	-	-	-	0.3	-
290	Pilsner	No	South Africa	4.5	-	4	1.6	1	0.3	-
298	Pale Lager	No	Jamaica	4.7	-	-	-	4	0.3	7
336	California Common	Yes	United States	5.5	-	23	-	1	0.3	8
342	Pilsner	Yes	United States	4.9	-	14	-	1	0.3	4
386	Pale Lager	No	Spain	4.8	-	-	-	6	0.3	7
388	Pale Lager	No	Spain	5.4	-	-	-	-	0.3	22
390	Pale Lager	No	Spain	4.8	-	-	-	2	0.4	23
392	Pale Lager	No	Peru	5.0	-	-	-	-	0.3	21

Sample number	Designated Style	Craft	Country	%ABV	Mycotoxins (µg/L)					
					AFB_1	DON	ZEN	T-2	OTA	FB_1
397	Imperial Pilsner	No	Spain	6.5	-	18	-	-	0.3	35
399	Adjunct Lager	No	Spain	5.5	-	-	-	-	0.3	36
404	Pale Lager	No	South Africa	5.0	-	-	0.4	-	-	3
447	Pale Lager	No	Spain	4.8	-	-	-	9	-	51
455	Zwickel/Keller/Landbier	No	Netherlands	5.0	-	-	1.7	8	0.1	15
459	Specialty Grain	No	Germany	5.0	-	15	-	8	0.2	3
465	Pale Lager	No	Mexico	4.5	-	-	-	9	0.1	18
475	California Common	Yes	Denmark	3.5	-	-	-	8	0.4	21
476	Speciality Grain	Yes	Netherlands	6.5	-	6	-	3	-	21
483	Pale Lager	No	Italy	4.8	-	-	-	3	0.2	33
484	Pilsner	No	Italy	5.0	-	-	-	2	0.2	29
485	Pale Lager	No	Italy	3.5	-	8	-	2	0.3	45
486	Pale Lager	No	Italy	4.8	-	-	-	3	0.3	8
487	Pale Lager	No	Italy	5.0	-	-	-	-	0.2	-
488	Pale Lager	No	Italy	4.7	-	-	-	-	0.2	71
489	Pale Lager	No	Italy	4.7	-	-	-	1	0.2	36
491	Pale Lager	No	Italy	3.5	-	34	-	-	0.2	64
492	Premium Lager	No	Italy	5.1	-	6	-	-	-	40
493	Pale Lager	No	Italy	4.7	-	-	-	-	-	59
495	Pale Lager	No	Italy	4.6	-	11	-	-	0.1	45
496	Pale Lager	No	Italy	4.8	-		-	-	-	10
497	Pale Lager	No	Italy	4.7	-	7	-	-	0.1	55
498	Pale Lager	No	Italy	4.5	-	-	-	-	-	56
499	Pale Lager	No	Italy	4.5	-	20	-	-	0.2	52
525	Pale Lager	No	India	4.8	-	-	-	7	0.3	25
526	Pilsner	No	Belgium	5.2	-	-	1.2	4	0.3	38
528	Pilsner	No	Netherlands	5.0	0.6	-	-	5	0.2	28
529	Pilsner	No	Netherlands	5.0	-	-	1.2	9	0.4	29
531	Pilsner	No	Netherlands	7.5	-	-	0.8	8	0.3	39
536	Pilsner	No	Netherlands	5.0	-	-	-	7	0.3	23
538	Pilsner	No	Netherlands	5.0	-	-	-	16	0.5	64
539	Pilsner	No	Netherlands	5.0	-	-	1.7	9	0.4	56
541	Pilsner	No	Netherlands	5.1	-	-	0.8	1	-	33
542	Pilsner	Yes	Netherlands	5.0	-	-	-	8	0.5	39
546	Pilsner	No	Netherlands	5.1	-	-	-	9	0.4	53
547	Pilsner	No	Netherlands	5.0	-	-	-	6	0.2	7
548	Pilsner	No	Germany	5.0	-	-	0.4	5	0.2	6
549	Pilsner	No	Netherlands	5.0	0.18	-	0.4	4	0.2	14
558	Pilsner	No	Netherlands	5.3	-	4	-	6	0.4	20
560	Imperial Pilsner	No	Netherlands	5.5	0.19		-	3	0.1	4
567	Pilsner	No	Netherlands	5.0	-	6	-	3	0.1	15
575	Pilsner	No	Germany	4.9	0.17	6	-	5	0.3	13

578	Adjunct Lager	No	Australia	5.0	-	-	-	6	0.2	15
627	Pilsner	No	Germany	4.8	-	-	-	3	0.3	1
643	Pilsner	No	Germany	4.9	0.12	-	-	9	0.3	11
698	Lager	No	Poland	5.2	-	70	-	-	0.2	5
704	Pilsner	No	Germany	4.9	-	-	-	3	0.2	-
706	Kellerbier	No	Germany	5.3	-	-	-	5	0.3	-
709	Pilsner	No	Germany	4.9	-	-	-	5	0.3	-
712	Adjunct Lager	Yes	USA	4.5	-	26	-	-	-	3
721	Pale Lager	No	Japan	5.0	-	-	0.5	7	0.2	-
728	Pale Lager	No	Japan	5.0	-	-	0.1	9	0.2	-
736	Lager	Yes	Denmark	5.6	-	-	-	7	0.3	-
774	Pale Lager	No	South Africa	4.5	-	-	-	-	0.1	-
775	Pale Lager	No	South Africa	5.0	-	-	-	-	0.1	3
776	Pale Lager	No	South Africa	5.5	-	-	-	-	0.1	-
777	Pale Lager	No	South Africa	3.5	-	-	-	-	0.2	2
785	Specialty Grain	No	Germany	4.5	-	42	1.2	3	1.1	13
875	Specialty Grain	Yes	Netherlands	5.0	-	-	-	-	0.1	-
895	Specialty Grain	No	Germany	5.0	-	-	-	-	-	-
925	Pale Lager	No	Spain	4.8	0.59	-	-	-	-	17
926	Pale Lager	No	Spain	5.4	-	-	-	-	-	4
927	Pale Lager	No	Spain	4.8	-	-	-	-	-	23
928	Pale Lager	No	Spain	4.2	-	-	-	1	0.2	3
930	Pale Lager	No	Spain	4.8	-	-	-	-	-	72
932	Pale Lager	No	Spain	5.5	-	-	-	1	-	3
933	American Style Lager	No	Spain	5.0	-	-	3.3	-	-	51
934	Pilsner	No	Spain	5.5	-	-	3.6	-	-	10
935	Pale Lager	No	Spain	4.8	-	-	5.6	-	-	11
936	Imperial Pilsner	No	Spain	6.4	-	-	-	-	-	24
938	American Style Lager	No	Spain	5.0	-	-	-	-	0.2	20
939	Pale Lager	No	Spain	4.8	-	-	-	-	0.3	-
941	Pale Lager	No	Spain	5.8	-	-	-	-	0.2	26
942	Pale Lager	No	Spain	6.2	-	-	-	-	0.2	-
943	American Style Lager	No	Spain	4.8	-	-	-	-	0.2	-
945	Pale Lager	No	Spain	5.4	-	-	-	-	-	6
946	Specialty Grain	No	Spain	5.4	-	-	-	-	-	-
947	Pale Lager	No	Spain	4.0	-	-	-	-	-	-
968	Pilsner	Yes	Nederland	5.0	-	-	-	-	0.2	-
1028	Pale Lager	No	Spain	4.2	-	-	-	-	0.1	-
1029	Pale Lager	No	Spain	5.4	-	-	-	-	0.1	6
1040	Adjunct Lager	No	Portugal	5.6	-	-	-	-	0.1	-
1051	Adjunct Lager	No	Portugal	5.1	-	-	-	-	0.1	-

- = no mycotoxins detected

S5 Table. M 6-plex immunoassay screening data saison beers

Sample Number	Designated Style	Craft	Country	%ABV	Mycotoxins (µg/L)					
					AFB_1	DON	ZEN	T-2	OTA	FB_1
144	Saison	No	Belgium	6.0	-	-	-	-	0.4	7
149	Biere de garde	Yes	France	6.9	-	-	-	-	0.3	-
227	Biere de garde	No	France	7.5	-	-	0.4	-	0.2	-
696	Saison	No	Belgium	6.5	-	-	0.4	2	0.3	-
735	Bière de Garde	Yes	USA	8.8	-	-	0.2	-	-	-
758	Saison	Yes	Canada	9.0	-	19	-	1	0.2	10
763	Saison	Yes	Japan	5.5	-	-	-	3	0.4	5
858	Saison	Yes	Belgium	6.0	-	-	-	-	-	-
861	Saison/Specialty Grain	Yes	USA	6.0	-	-	-	-	0.1	-
864	Saison	Yes	USA	10.4	-	-	-	-	-	-
878	Saison	Yes	Netherlands	8.5	-	-	-	-	0.4	1
908	Saison	Yes	Belgium	6.9	1.3	-	1.0	-	-	5
954	Saison	Yes	Latvia	4.5	-	-	-	-	-	-

- = no mycotoxins detected

S5 Table. N 6-plex immunoassay screening data smoked beers

Sample Number	Designated Style	Craft	Country	%ABV	Mycotoxins (µg/L)					
					AFB_1	DON	ZEN	T-2	OTA	FB_1
16	Smoked beer	Yes	Netherlands	7.5	-	11	-	-	0.1	-
178	Smoked beer	No	Germany	5.1	-	44	-	-	0.5	12
236	Smoked Bock	Yes	Belgium	6.7	-	62	-	-	-	2
369	Smoked	Yes	Netherlands	11.0	3.0	94	-	2	0.4	8
377	Smoked	Yes	Denmark	6.1	-	47	0.1	2	0.3	6
474	Smoked Ale	Yes	Belgium	6.5	0.1	9	-	8	-	30
543	Smoked	Yes	Netherlands	8.2	0.7	42	-	-	-	39
684	Smoked Barley-Wine Style Ale	Yes	Norway	11.0	-	4	-	-	0.3	2
694	Smoked	Yes	USA	7.5	-	1	-	1	0.1	2
827	Smoked	No	Germany	6.9	-	18	-	-	0.5	2
828	Smoked	No	Germany	5.1	0.1	-	-	1	0.4	6
829	Smoked	Yes	Netherlands	6.5	-	128	-	-	0.3	-
831	Smoked	No	Germany	5.2	-	-	-	-	0.3	3
833	Smoked	Yes	Netherlands	6.5	-	-	-	-	0.5	-
841	Smoked	Yes	Belgium	6.7	-	-	-	-	0.3	-
975	Smoked	Yes	USA	6.5	-	7	-	-	0.2	-

- = no mycotoxins detected

S5 Table. O 6-plex immunoassay screening data sour ale beers

**Sample Number**	**Designated Style**	**Craft**	**Country**	**%ABV**	**Mycotoxins (µg/L)**					
					**AFB_1**	**DON**	**ZEN**	**T-2**	**OTA**	**FB_1**
14	Geuze	Yes	Belgium	5.0						17
19	Geuze	Yes	Belgium	5.0	-	-	-	-		1
30	Geuze	Yes	Belgium	5.0	-	-	-	-		3
37	Geuze	No	Belgium	5.2	-	-	-	-		1
41	Flanders Oud Bruin	Yes	Belgium	8.0	-	1	-	-		
63	Geuze	Yes	Belgium	5.0	-	-	-	-		10
86	Sour/Lambic	Yes	United States	10.0	-	-	-	-	0.1	10
90	Geuze	Yes	Belgium	6.0	-	-	-	-		2
119	Geuze	Yes	Belgium	5.0	-	-	-	-		
140	Geuze	Yes	Belgium	6.0	-	-	-	-		2
143	Geuze	Yes	Belgium	6.0	-	-	-	-		
147	Geuze	Yes	Belgium	6.0	-	-	-	-	0.5	14
152	Geuze	Yes	Belgium	6.5	-	-	-	-	0.6	
156	Geuze	Yes	Belgium	6.0	-	-	-	-	-	
170	Sour Ale/Wild Ale	Yes	France	7.0	-	-	-	-	-	
174	Geuze	Yes	Belgium	6.0	-	-	-	-	-	15
188	Belgain Strong Ale	Yes	Swiss	11.0	-	-	-	-	-	-
233	Sour Ale/Wild Ale	No	Belgium	7.3	-	-	-	-	-	-
291	Geuze	Yes	Belgium	5.0	-	-	0.1	13	-	17
295	Flanders Oud Bruin	Yes	Belgium	13.0	-	-	1.3	-	-	-
296	Geuze	Yes	Belgium		-	-		-	-	25
301	Geuze	Yes	Belgium	7.0	-	116	0.4	-	-	38
303	Sour Ale/Wild Ale	Yes	Denmark	10.0	-		1.2	-	-	-
310	Geuze	Yes	Belgium	8.0	-	25	-	-	-	-
313	Lambic	Yes	Belgium	8.0	-	-	-	-	-	8
319	Geuze	Yes	Belgium	5.0	-	-	-	-	-	32
322	Geuze	Yes	Belgium	nd	-	-	-	-	-	-
327	Lambic	Yes	Italy	8.0	-	-	-	-	-	5
328	Lambic	Yes	Italy	6.2	-	21	-	-	-	8
334	Geuze	Yes	Belgium	6.0	-	8	-	-	-	3
340	Lambic	Yes	Belgium	8.0	-	-	-	-	0.3	28
343	Lambic	Yes	Belgium	6.0	-	-	-	-	-	
358	Lambic	Yes	Italy	6.2	-	-	-	-	-	2
359	Lambic	Yes	Denmark	7.7	-	-	-	-	-	11
364	Lambic	Yes	Italy	6.7	-	-	-	-	-	5
383	Lambic	Yes	Denmark	5.0	-	-	-	-	-	9
433	Lambic	Yes	Belgium	5.0	-	-	-	-	-	54
445	Sour Ale/Wild Ale	Yes	Denmark	8.5	-	-	-	-	-	-
522	Sour Ale/Wild Ale	Yes	Italy	6.2	-	-	-	-	-	-
551	Sour Ale	No	Belgium	6.0	-	-	0.2		-	7
562	Sour Ale	No	Belgium	6.2	-	-	-	2	-	19

Sample number	Designated Style	Craft	Country	%ABV	Mycotoxins (µg/L)					
					AFB_1	DON	ZEN	T-2	OTA	FB_1
566	Lambic	No	Belgium	5.2	-	8	-	-	-	11
579	Sour Ale/Wild Ale	Yes	USA	7.0	-	-	0.3	-	-	-
591	Lambic	Yes	Belgium	5.0	-	-	-	-	-	-
597	Lambic	Yes	Italy	9.0	-	14	-	4	-	15
601	Geuze	Yes	Belgium	5.0	-	-	-	-	-	-
610	Lambic	Yes	Belgium	6.0	-	-	-	-	-	10
615	Lambic	Yes	Belgium	6.0	-	-	-	-	-	-
635	Geuze	No	Belgium	5.0	-	-	-	-	-	-
636	Geuze	Yes	Belgium	6.0	-	-	-	-	-	-
640	Sour Ale/Wild Ale	Yes	USA	6.8	-	-	0.1	10	-	10
646	Lambic	Yes	Belgium	5.0	-	-	-	4	-	8
650	Faro	Yes	Belgium	8.0	-	-	-	-	-	-
653	Geuze	Yes	Belgium	6.0	-	-	-	-	-	6
670	Lambic	Yes	Belgium		-	-	-	-	-	-
677	Lambic	Yes	Denmark	8.0	-	-	-	-	-	10
679	Gose	Yes	USA	4.4	-	-	-	-	-	1
680	Wild Ale	Yes	Belgium	7.0	-	-	-	-	-	1
685	Sour Ale/Wild Ale	Yes	Belgium	10.0	-	15	-	-	-	-
690	Lambic	Yes	Belgium	5.0	-	-	-	-	-	2
702	Lambic	Yes	Belgium	8.0	-	-	-	-	-	-
705	Geuze	Yes	Belgium	6.0	-	-	-	2	0.3	-
708	Geuze	Yes	Belgium	8.0	-	8	-	-	-	2
725	Lambic	Yes	Belgium	5.0	-	-	0.8	31	0.3	18
731	Lambic	Yes	Belgium	6.0	-	-	-	-	-	1
743	Sour Ale/Wild Ale	Yes	Netherlands	4.6	-	-	-	4	0.1	-
749	Berliner weisse	Yes	Denmark	3.4	-	-	-	-	-	-
773	Lambic	No	Belgium	3.2	-	-	-	-	-	-
782	Lambic	No	Belgium	4.0	-	36		8		19
787	Geuze	Yes	Belgium	4.5	-	40	-	-	0.7	13
817	Lambic	Yes	Belgium	6.0	-	62	-	-	1.2	-
819	Geuze	Yes	Belgium	6.0	-	133	-	-	1.6	-
821	Geuze	Yes	Belgium	5.2	-	141	-	-	0.9	-
847	Geuze	Yes	Belgium	5.5	-	-	-	-	-	-
852	Geuze	Yes	Belgium	6.0	-	-	-	-	-	-
857	Geuze	Yes	Belgium	5.0	-	-	-	-	-	-
879	Lambic	No	Belgium	6.5	-	-	-	-	-	-
886	Sour red/Brown	No	Belgium	7.0	-	-	-	-	0.9	-
887	Sour red/Brown	Yes	Belgium	13.0	-	-	-	-	-	-
953	American Wild Ale	Yes	USA	7.5	-	-	-	-	-	-
960	Grodziskie/gose/lichtenhainer	Yes	Poland	2.6	-	-	-	-	0.3	-
970	American Wild Ale	Yes	USA	6.8	-	-	-	-	-	-

- = no mycotoxins detected

S5 Table. P 6-plex immunoassay screening data stout beers

Sample Number	Designated Style	Craft	Country	%ABV	Mycotoxins (µg/L)					
					AFB_1	DON	ZEN	T-2	OTA	FB_1
1	Wild Porter	Yes	Belgium	7.0	-	69	-	-	-	12
36	Stout	No	Ireland	4.1	-	34	-	-	0.3	11
77	Porter	No	England	5.4	-	45	-	-	0.5	20
138	Porter	No	Czech Republic	10.5	-	49	-	-	-	-
157	Oatmeal Stout	Yes	United States	5.7	-	100	-	2	0.3	28
160	Imperial Stout	Yes	Sweden	7.5	-	135	-	4	0.2	50
198	Imperial Stout	Yes	Netherlands	7.5	-	-	0.3	-	0.3	3
229	Imperial Stout	Yes	England	7.0	-	48	0.5	-	0.3	5
254	Sweet Stout	Yes	Netherlands	5.5	-	-	0.4	-	-	-
267	Milk Stout	No	Trinidad & Tobago	4.9	-	-	1.0	-	-	-
268	Foreign Stout	No	Malaysia	6.8	-	-	-	-	-	-
275	Foreign Stout	No	Nigeria	6.5	-	-	-	-	0.2	19
286	Milk Stout	No	South Africa	6.0	-	3	-	-	0.3	3
297	Porter	Yes	Germany	8.0	-	93	1.2	-		29
326	Imperial Stout	Yes	Italy	7.0	-	169		-	0.3	34
350	Porter	Yes	Denmark	6.6	-	108	3.0	-	0.2	27
357	Stout	Yes	England	5.5	-	38	-	3	0.3	13
431	Imperial Stout	Yes	England	7.0	0.2	49	-	6	0.2	96
469	Imperial Stout	Yes	Netherlands	7.5	-	12	-	2	0.2	8
473	Porter	Yes	United States	5.7	0.1		-	11	0.3	15
563	Imperial Stout	Yes	Netherlands	7.5	0.9	27	-	1	0.2	19
595	Porter	Yes	USA	6.7	-		-	3	0.3	9
600	Foreign Stout	No	England	7.4	1.9	70	-	2	0.2	57
632	Stout	Yes	England	5.0	-	14	-	1	0.3	7
634	Porter	Yes	USA	6.4	0.4	71	-	2	-	11
637	Stout	No	England	5.0	0.6	2	-	8	0.3	13
641	Porter	Yes	USA	6.4	-	75	0.3	-	-	7
647	Porter	Yes	USA	6.4	-	41	-	-	-	1
660	Porter	Yes	USA	5.9	0.2	-	-	6	0.4	2
661	Baltic Porter	No	Russia	7.0	-	1	-	5	0.4	4
681	Baltic Porter	Yes	USA	6.4	-	16	-	-	0.3	7
693	Stout	Yes	Denmark	7.7	-	3	-	-	-	1
699	Foreign Stout	No	Australia	6.3		24		1	0.2	5
707	Stout	Yes	Denmark	7.0	-	66	-		0.1	3
718	Porter	Yes	Denmark	6.8	-	12	-	6	0.4	4
722	Porter	Yes	USA	10.5	1.0	57	1.0	4	0.4	12
753	Porter	Yes	France	8.0	-	10	-	5	0.4	7
757	Stout	Yes	USA	7.1	-	43	-	3	0.4	21
802	Stout	Yes	Netherlands	7.4	2.8	124	-	4	-	29
812	Porter	Yes	England	4.5	-	-	-	-	-	-
813	Porter	Yes	USA	3.0	-	22	-	-	-	-

Sample number	Designated Style	Craft	Country	%ABV	Mycotoxins (µg/L)					
					AFB_1	DON	ZEN	T-2	OTA	FB_1
815	Foreign Stout	No	Netherlands	7.2	-	-	-	1	-	5
826	Porter	Yes	USA	6.8	0.1	-	-	1	0.5	-
851	Stout	Yes	Austria	4.4	-	-	-		0.5	3
855	Stout	No	England	2.8	-	-	-	-	-	-
881	Dry Stout	Yes	Belgium	7.0	-	12	-	2	0.5	4
903	Oatmeal Stout	Yes	USA	6.9	-	88	-	-	0.3	3
914	Porter	Yes	Iceland	6.0	1.2	-	-	2	0.2	20
916	Stout	Yes	Germany	3.3	-	-	-	7	0.2	24
917	Baltic Porter	No	Belarus	6.8	0.7	-	-	1	-	3
949	Foreign Stout	Yes	Poland	6.2	-	-	-	-	0.3	-
973	Imperial Stout	Yes	Netherlands	7.5	-	-	-	-	-	-
976	Stout	No	Belgium	7.7	-	-	-	-	-	-
977	Stout	No	Belgium	6.5	-	-	-	2	-	-
995	Stout	Yes	Scotland	6.8	-	24	-	-	-	-

- = no mycotoxins detected

S5 Table. Q 6-plex immunoassay screening data strong dark ale beers

Sample Number	Designated Style	Craft	Country	%ABV	Mycotoxins (µg/L)					
					AFB$_1$	DON	ZEN	T-2	OTA	FB$_1$
22	Double	No	Belgium	8.0	-	13	-	-	0.3	-
25	Belgian Strong Dark Ale	Yes	Belgium	9.2	-	15	-	-	0.3	-
26	Quadrupel	No	Belgium	11.0	-	8	-	-	0.2	-
27	Quadrupel	No	Belgium	11.0	-	32	-	-	0.2	-
146	Quadrupel	Yes	Belgium	11.3	-	13	-	-	-	-
159	Abbey Dubbel	Yes	Belgium	8.0	-	19	-	3	0.2	2
171	Belgian Strong Dark Ale	Yes	United States	10.0	-	63	-	-	0.5	10
172	Quadrupel	No	Netherlands	10.0	-	33	-	-	0.3	-
189	Quadrupel	No	Belgium	10.0	-	-	-	-	-	-
203	Quadrupel	Yes	Belgium	10.0	-	-	-	-	0.2	1
204	Belgian Strong Ale	Yes	Belgium	9.0	-	-	-	-	0.3	-
219	Belgian Strong Ale	Yes	Netherlands	9.5	-	73	0.6	-	0.3	2
256	Belgian Strong Dark Ale	Yes	Belgium	9.5	-	-	-	1	0.2	-
324	Black & Tan	Yes	Netherlands	10.0	-	95	-	-	0.2	12
331	Quadrupel	Yes	Netherlands	10.0	-	22	-	-	-	9
333	Belgian Strong Ale	No	Belgium	11.0	-	27	-	-	-	6
435	Belgian Strong Ale	No	Belgium	10.0	-	-	-	8	-	-
444	Quadrupel	Yes	Belgium	10.2	-	40	-	3	-	-
450	Belgian Strong Ale	Yes	Belgium	10.0	0.3	19	-	5	-	14
462	Belgian Strong Ale	No	Belgium	10.0	-	-	-	-	-	-
471	Quadrupel	No	Belgium	11.0	-	37	-	1	-	4
561	Belgian Strong Ale	No	Belgium	9.0	-	9	-	1	0.1	5
568	Quadrupel	No	Belgium	11.0		3	-	-	0.0	8
577	Weizen Bock	Yes	Netherlands	9.0	1.7	23	-	2	0.3	52
584	Quadrupel	Yes	USA	11.8	0.3	15	-	-	-	35
586	Belgian Strong Ale	Yes	Netherlands	10.0	1.3	28	-	-	-	26
608	Belgian Strong Ale	No	Belgium	9.0	2.1	46	-	2	-	16
609	Belgian Strong Ale	No	Belgium	9.0	2.5	52	2.6	2	-	13
611	Belgian Strong Ale	No	Belgium	9.0	-	117	-	2	-	9
620	Belgian Strong Ale	Yes	Belgium	10.0	0.4	58	-	-	0.1	2
621	Belgian Strong Ale	Yes	Belgium	10.0	-	53	-	-	0.2	1
633	Belgium Strong Ale	Yes	Belgium	10.0	-	26	-	-	0.2	2
740	Quadrupel	Yes	Canada	10.5	-	2	-	7	0.3	1
744	Quadrupel	Yes	Belgium	11.0	-	3	-	2	0.2	
752	Quadrupel	Yes	Belgium	12.0	-	5	-	4	0.1	2
784	Quadrupel	No	Belgium	10.5	-	44	-	-	0.7	14
842	Belgium Strong Ale	Yes	Belgium	9.0	-	15	-	-	0.6	-
873	Quadrupel	Yes	Austria	9.7	-	-	-	-	0.1	-
912	Belgian Strong Dark Ale	Yes	Nederland	9.0	-	-	0.7	-	-	4
956	Strong Dark Ale	No	Belgium	13.0	-	-	-	-	-	-
966	Abbey Dubbel	Yes	Belgium	8.0	-	-	-	-	0.2	-

Sample number	Designated Style	Craft	Country	%ABV	Mycotoxins (µg/L)					
					AFB_1	DON	ZEN	T-2	OTA	FB_1
974	Abbey Dubbel	Yes	Netherlands	8.5	-	-	-	-	0.2	-
983	Strong Dark Ale	Yes	Belgium	10.2	-	-	-	-	-	-
984	Quadrupel	Yes	Denmark	10.0	-	-	-	-	-	-

- = no mycotoxins detected

S5R Table. R 6-plex immunoassay screening data strong pale ale beers

Sample Number	Designated Style	Craft	Country	%ABV	Mycotoxins (µg/L)					
					AFB$_1$	DON	ZEN	T-2	OTA	FB$_1$
5	Barley Wine	Yes	Denmark	10.0	-	50	-	-	0.2	17
17	Barley Wine	Yes	Denmark	12.0	-	79	-	-	-	-
18	Barley Wine	Yes	Netherlands	10.5	-	25	-	-	-	-
28	Barley Wine	Yes	United States	11.0	-	94	-	-	0.3	4
69	Belgian Strong Ale	No	Belgium	9.5	-	-	-	-	0.2	-
84	Abbey Tripel	No	Belgium	9.0	-	-	-	-	-	-
101	Belgian Strong Blond Ale	No	France	11.8	-	-	-	-	0.3	-
150	Barley Wine	Yes	United States	11.5	-	76	-	-	-	1
164	Belgian Strong Dark Ale	Yes	Belgium	9.0	-	81	-	-	-	10
165	Abbey Tripel	Yes	Belgium	9.5	-		-	-	0.3	-
168	Abbey Tripel	Yes	Netherlands	9.0	-	27	-	-	0.3	7
175	Barley Wine	Yes	United States	11.0	-	58	-	-	0.3	15
185	Barley Wine	Yes	England	11.5	-	40	0.9	-	0.2	2
205	Strong Pale Ale	No	Poland	7.0	-	38	0.1	-	-	-
206	Belgian Strong Ale	No	Germany/United States	10.0	-		0.3	-	0.4	-
230	Abbey Tripel	Yes	Netherlands	9.0	-	1	-	-	0.4	1
305	Abbey Tripel	No	Belgium	9.0	-	12	0.5	-	-	-
309	Barley Wine	Yes	Denmark	10.4	-	28	1.3	-	-	10
311	Barley Wine	Yes	United States	9.6	-	43	-	-	0.2	8
337	Barley Wine	Yes	Denmark	10.5	-	76	-	-	-	10
339	Barley Wine	Yes	England	10.3	-	26	-	-	0.3	8
353	Barley Wine	Yes	England	11.0	-	-	-	2	0.6	3
355	Barley Wine	Yes	Netherlands	10.0	-	37	-	-	-	5
363	Belgian Ale	Yes	Belgium	10.0	-	51	-	-	0.2	6
368	Belgian Ale	Yes	Denmark	9.2	-	88	-	1	0.2	11
382	Abbey Tripel	Yes	Belgium	10.0	-	32	-	3	0.3	1
384	Barley Wine	Yes	Netherlands	10.3	-	74	-	1	0.4	10
463	Abbey Tripel	Yes	Netherlands	9.5	0.1	-	-	9	0.1	19
470	Barley Wine	Yes	United States	11.0		18	-	2		15
472	American Strong Ale	Yes	Denmark	9.3	0.3	17	-	8	0.3	39
478	Barley Wine	Yes	Denmark	18.5	0.5	30	-	-	-	9
480	Barley Wine	Yes	Netherlands	9.0	0.6	16	-	-	-	9
481	Belgian Strong Ale	Yes	Netherlands	9.0	1.2	13	-	-	-	16
508	Barley Wine	Yes	England	10.2	0.4	42	-	-	-	-
509	Abbey Tripel	Yes	Netherlands	9.5	0.6	17	-	-	-	-
553	Abbey Tripel	No	Belgium	7.5	-	-	0.1	3	0.2	4
602	Belgian Strong Ale	No	Belgium	12.0	-	-	-	1	-	-
603	Strong Pale Ale	No	Belgium	10.0	-	-	-	2	-	-
606	Strong Pale Ale	No	Belgium	10.5	-	-	-	-	-	-
629	Abbey Tripel	Yes	Denmark	9.3	-	16	-	-	0.1	1
645	Barley Wine	Yes	USA	13.5	-	56	-	-	0.1	9

Sample number	Designated Style	Craft	Country	%ABV	Mycotoxins (µg/L)					
					AFB_1	DON	ZEN	T-2	OTA	FB_1
668	Abbey Tripel	No	Belgium	10.0	-	-	-	1	-	-
673	Belgian Strong Ale	No	Belgium	9.0	-	1	-	-		3
703	Barley Wine	Yes	USA	13.4	-	28	-	-	0.2	2
724	American Strong Ale	Yes	USA	10.5	-	16	-	4	0.3	5
737	Barley Wine	Yes	Denmark	10.0	-	10	-	2	0.1	1
759	Barley Wine	Yes	Netherlands	15.2	-	-	-	-	0.2	-
760	Barley Wine	Yes	USA	13.0	-	13	-	-	0.1	3
768	Barley Wine	Yes	Norway	10.0	-	7	-	-	0.1	2
769	American Barley Wine	Yes	USA	11.0	-	-	-	-	0.2	-
806	Strong Pale Ale	Yes	Belgium	10.0	2.6	-	-	-		3
809	Barley Wine	Yes	USA	10.2	-	45	-	-	0.1	-
836	Belgian, Strong Ale	No	France	9.0	-	11	-	-	0.3	1
840	Abbey Tripel	Yes	Belgium	8.5	-	-	-	-	0.5	-
844	Barley Wine	Yes	Denmark	11.0	-	18	0.1	-	0.4	4
849	Barley Wine	Yes	USA	11.5	-	10	-	-	0.6	3
872	American Strong Ale	Yes	USA	10.1	-	-	-	-	-	-
888	Abbey Tripel	Yes	Netherlands	7.8	-	-	-	-	-	-
898	Strong Pale Ale	Yes	Nederland	9.0	0.2	-	-	-	0.3	-
910	Barley Wine	Yes	Nederland	10.0	2.4	2	-	-	-	-
915	Barley Wine	Yes	Nederland	10.0	-	-	-	-	-	5
920	American Strong Ale	Yes	USA	11.6	-	-	0.2	-	-	-
923	Barley Wine	Yes	Nederland	12.0	-	12	-	-	-	2
972	Strong Pale Ale	Yes	Belgium	9.5	-	-	-	-	-	-
985	Barley Wine	Yes	Netherlands	15.2	-	1	-	-	-	1
994	Barley Wine	Yes	France	10.5	-	30	-	-	0.3	3
1012	English Barley Wine	Yes	Netherlands	12.0	-	9	-	-	-	-
1042	Scotch Ale	Yes	Scotland	8.5	-	7	-	-	0.1	-

- = no mycotoxins detected

S5 Table. S 6-plex immunoassay screening data strong pale lager

Number	Designated Style	Craft	Country	%ABV	Mycotoxins (µg/L)					
					AFB_1	DON	ZEN	T-2	OTA	FB_1
402	Strong Pale Lager/Imperial Pilsner	No	Spain	6.4		2		2	0.3	27
442	Strong Pale Lager/Imperial Pilsner	No	Netherlands	11.6		38		2		
460	Strong Pale Lager/Imperial Pilsner	No	Austria	14.0	0.4	41			0.2	23
614	Strong Pale Lager/Imperial Pilsner	No	Spain	6.4						48
642	Strong Pale Lager/Imperial Pilsner	No	Austria	14.0		10				7
805	Strong Pale Lager/Imperial Pilsner	No	Poland	6.2	2.9					11
808	Vintage Strong Pale Lager	No	Switzerland	14.0						
929	Strong Pale Lager/Imperial Pilsner	No	Spain	7.2						1

- = no mycotoxins detected

S5 Table. T 6-plex immunoassay screening data wheat beers

Sample Number	Designated Style	Craft	Country	%ABV	Mycotoxins (µg/L)					
					AFB$_1$	DON	ZEN	T-2	OTA	FB$_1$
454	Dunkel Weizen	No	Germany	5.0	-	17	-	7		25
9	Weizen	No	Germany	5.5	-	39	-	-	0.2	7
15	Weizen	No	Germany	5.6	-	6	-	-	0.3	3
58	Weizen	Yes	Netherlands	6.0	-	19	-	-	-	-
74	Weizen	No	Germany	5.5	-	-	-	-	-	1
76	Weizen	Yes	Germany	5.0	-	-	-	-	0.3	2
80	Weizen	No	Germany	5.4	-	-	-	-	-	9
82	White beer	Yes	Netherlands	5.5	-	11	-	-	0.2	2
92	Weizen	No	Germany	5.9	-	1	-	-	0.2	6
98	WeizenBock	Yes	Netherlands	6.4	-	8	-	-	0.3	12
100	Weizen	No	Germany	5.1	-	-	-	-	0.2	6
102	Witbier	No	Netherlands	5.5	-	-	-	-	0.3	-
104	Witbier	No	Netherlands	5.0	-	-	-	-	-	-
113	Belgian White	No	Netherlands	5.0	0.3	36	-	-	0.1	19
141	Witbier	No	Belgium	4.9	-	41	-	-	0.3	
182	Dunkel Weizen	No	Germany	5.2	-	44	-	-	0.3	20
202	Dunkel Weizen	Yes	Poland	5.0	-	48	-	-	0.3	2
207	Weizen	Yes	Poland	5.0	-	9	-	-	0.4	1
213	Belgian White	Yes	Netherlands	5.0	-	-	-	-	0.3	2
228	Dunkel Weizen	No	Germany	5.6	-	3	-	-	0.8	4
231	WeizenBock	No	Germany	8.2	-	30	-	-	0.2	4
245	Weizen	No	Germany	5.3	-	-	0.9	-	0.3	-
360	Wheatbeer	Yes	Netherlands	6.0	-	1	-	-	0.3	10
371	Wheatbeer	Yes	Netherlands	6.0	-	19	-	-	0.1	2
400	Wheat Ale	No	Spain	5.2	-	-	-	-	0.3	23
441	German HefeWeizen	No	Germany	5.6	-	-	-	8	-	30
448	Weizen Bock	Yes	Germany	7.3	0.1	-	-	9	-	55
458	Weizen Bock	No	Germany	7.7	-	125	-	-	-	34
533	White beer	No	Netherlands	5.0	1.2	9	0.7	8	0.2	36
540	White beer	No	Belgium	4.9	-	-	0.7	11	0.3	23
550	White beer	No	Netherlands	5.0	0.7	26	-	4	0.1	12
555	White beer	Yes	Netherlands	8.0	-	7	-	2	0.2	12
689	Weizen Bock	No	Germany	7.3	-	-	-	-	0.2	2
691	German HefeWeizen	No	Germany	5.6	-	-	-	-	0.1	1
713	German HefeWeizen	Yes	Netherlands	5.0	-	-	-	4	0.2	-
714	German HefeWeizen	No	Germany	5.6	-	-	-	2	0.3	-
729	Weizen	Yes	Denmark	4.9	-	-	0.1	6	0.1	-
783	Belgian White	No	USA	5.0	-	106	0.6	1	0.9	14
804	German HefeWeizen	No	Poland	5.2	3.3	-	-	2	-	20
874	Weizen Bock	No	Germany	8.2	-	-	-	-	-	-
948	Wheat Ale	Yes	Poland	5.0	-	-	-	-	0.3	-

Sample number	Designated Style	Craft	Country	%ABV	Mycotoxins (µg/L)					
					AFB_1	DON	ZEN	T-2	OTA	FB_1
962	Belgian White	No	Netherlands	5.6	-	-	-	-	-	-

- = no mycotoxins detected
