# Supplementary material for: Mycotoxin profiling of 1000 beer samples with a special focus on craft beer
Source: PLoS One. 2017 Oct 5;12(10):e0185887. doi: 10.1371/journal.pone.0185887 (PMC5628871; doi:10.1371/journal.pone.0185887)
Supplement: S6 Table — (PDF) [file pone.0185887.s010.pdf]

S6 Table Reassessment of two Spanish breweries for FB contaminations in beer (µg/L) by LC-MS/MS analysis

**Sample Number**	**Brewery**	**Beer**	**FB_1**	**FB_2**	**FB_3**	**Sum FBs**
936	Brewery 1	beer 1	12	3	2	17
980	Brewery 1	beer 2	7	5	2	14
930	Brewery 2	beer 1	26	7	3	37
934	Brewery 2	beer 2	3	3	1	7
941	Brewery 2	beer 3	56	7	5	69
944	Brewery 2	beer 4	22	15	4	41
