# Supplementary material for: Mycotoxin profiling of 1000 beer samples with a special focus on craft beer
Source: PLoS One. 2017 Oct 5;12(10):e0185887. doi: 10.1371/journal.pone.0185887 (PMC5628871; doi:10.1371/journal.pone.0185887)
Supplement: S7 Table — (PDF) [file pone.0185887.s011.pdf]

S7 Table DON+D3G contamination per beer style group

Beer Style Group	DON+D3G contamination 6-plex immunoassay			Suspects (from total)	Percentage (%)
	Average (μg/L)	Median (μg/L)	Range (μg/L)		
Non/low alcohol	23	23	11 - 36	12 (36)	33
Pale Lager	23	19	10 - 70	22 (166)	13
Strong Pale Lager	30	38	10 - 41	3 (7)	43
Pale Ale	38	30	11 - 147	27 (94)	29
Strong Pale Ale	36	28	10 - 139	41 (70)	59
India Pale Ale	47	26	11 - 146	15 (43)	35
Double India Pale Ale	45	52	12 - 97	9 (40)	23
Dark lager	49	45	11 - 119	19 (28)	68
Dark Ale	41	22	10 -121	8 (26)	31
Strong Dark Ale	40	32	13 - 117	24 (45)	53
Stout	57	48	12 - 267	30 (55)	54
Imperial Stout	86	79	14 - 475	105 (126)	83
Sour ales	30	21	14 - 116	6 (82)	7
Fruit/Vegetable/Spice	56	49	12 - 127	12 (37)	32
Saison	19	nd	nd	1 (13)	8
Smoked	46	44	11 - 219	7 (16)	44
Wheat	40	36	11 - 125	14 (42)	33
Bock	33	28	10 - 97	20 (38)	53
Eisbock	81	27	14 - 308	5 (6)	83
African Traditional	65	57	10 - 141	15 (30)	50
